# EPHB2 drives EMT-linked vasculogenic mimicry and cervical cancer progression via an ERK–ETV4 transcriptional program

**DOI:** 10.1186/s12967-026-08300-0

**Published:** 2026-05-16

**Authors:** Wenzhi Jiao, Shanshan Liu, Qingyang Huai, Ziyue Zhang, Qinyuan He, Xiaoqin Yang, Minmin Yu

**Affiliations:** 1 Department of Gynecology, The Second Hospital of Nanjing, Nanjing University of Chinese Medicine, Nanjing, Jiangsu 210003 China; 2 School of Life Sciences, Suzhou Medical College of Soochow University, Suzhou, Jiangsu 215123 China; 3 School of Biology and Basic Medical Sciences, Suzhou Medical College of Soochow University, Suzhou, Jiangsu 215123 China

**Keywords:** EPHB2, Cervical cancer, Vasculogenic mimicry, EMT (Epithelial–mesenchymal transition), Therapeutic target

## Abstract

**Background:**

Vasculogenic mimicry (VM) and epithelial–mesenchymal transition (EMT) contribute to cervical cancer invasiveness and treatment resistance, yet the upstream regulators coordinating these plasticity programs remain incompletely defined. Ephrin receptor B2 (EPHB2) promotes malignant phenotypes in multiple tumor types; however, its functional role and mechanistic basis in cervical cancer, particularly in VM-associated plasticity, remain unclear.

**Methods:**

EPHB2 dysregulation was screened in public transcriptomic cohorts (GSE168244, tumor vs. normal; GSE138080, precancer vs. normal) and validated at the protein level in an institutional immunohistochemistry (IHC) cohort. Its clinical relevance was assessed in TCGA-CESC with GTEx normal cervix controls for tumor–normal comparison and survival analysis. Single-cell RNA-seq data (GSE168652) were used to map EPHB2 expression and characterize epithelial functional states. Gain- and loss-of-function assays in HeLa and SiHa cells evaluated proliferation, clonogenicity, migration, apoptosis, EMT marker expression, and Matrigel-based capillary-like network formation. In vivo validation was performed in SiHa xenografts with stable EPHB2 knockdown. Mechanistic analyses integrated two independent RNA-seq comparisons after EPHB2 silencing with pathway enrichment, qRT–PCR and Western blot validation, and functional interrogation of ETV4.

**Results:**

EPHB2 was consistently upregulated in cervical cancer across multiple cohorts, including precancerous lesions, and showed increased protein abundance in tumor tissues. In TCGA-CESC, high EPHB2 expression was associated with poorer overall survival and enhanced EMT activity. Single-cell analysis linked EPHB2-positive/high epithelial cells to EMT/VM-related epithelial states. Functionally, EPHB2 silencing inhibited proliferation, colony formation, migration, and capillary-like network formation, increased apoptosis, and shifted EMT markers toward an epithelial phenotype, whereas EPHB2 overexpression produced reciprocal effects. Stable EPHB2 knockdown further suppressed xenograft growth and altered angiogenesis/VM- and EMT-related features in vivo. Mechanistically, two independent RNA-seq datasets converged on “Transcriptional misregulation in cancer” as the only shared tumor-relevant enriched pathway. Subsequent validation supported an EPHB2–ERK–ETV4 module accompanied by coordinated changes in DUSP6 and CXCL8. ETV4 depletion impaired proliferation and VM-like network formation and attenuated the growth advantage conferred by EPHB2 overexpression.

**Conclusions:**

EPHB2 promotes cervical cancer progression by sustaining EMT-linked plasticity and VM-like behavior, at least in part through an ERK–ETV4-centered transcriptional program. These findings identify EPHB2 as a candidate biomarker of aggressive cervical cancer and a potential therapeutic entry point for further investigation.

**Supplementary Information:**

The online version contains supplementary material available at 10.1186/s12967-026-08300-0.

## Introduction

Cervical cancer remains a major global health concern, ranking as the fourth most common malignancy among women and a leading cause of cancer-related mortality in low- and middle-income countries. According to GLOBOCAN 2020, more than 600,000 new cases and 340,000 deaths were reported worldwide, underscoring a substantial disease burden despite the implementation of human papillomavirus (HPV) vaccination and screening programs [1, 2]. Although early-stage cervical cancer can be effectively managed with surgery and chemoradiotherapy, outcomes for patients with advanced or recurrent disease remain poor, with a 5-year overall survival rate of less than 20% [3]. Tumor recurrence and therapeutic resistance are frequently associated with molecular alterations that promote angiogenesis-independent vascularization, epithelial–mesenchymal transition (EMT), and immune evasion, highlighting the need to identify novel biomarkers and therapeutic targets [4, 5].

Ephrin receptor B2 (EPHB2), a member of the Eph receptor tyrosine kinase family, plays pivotal roles in developmental processes, including axon guidance, angiogenesis, and cell–cell communication [6, 7]. Aberrant EPHB2 signaling has been reported in multiple cancers, including colorectal carcinoma, glioblastoma, and hepatocellular carcinoma, where it contributes to tumor progression, metastasis, and poor clinical prognosis [8–10]. However, its role in cervical cancer remains poorly understood. Recent evidence suggests that EPHB2 may influence tumor cell plasticity and invasive behavior, raising the possibility that it serves as a regulator of malignant phenotypes, including vasculogenic mimicry (VM) and EMT [11, 12].

VM, first described by Maniotis et al. in aggressive melanoma, refers to the ability of tumor cells to form perfusable, matrix-rich vascular channels independent of endothelial cells [13]. Unlike classical angiogenesis, VM represents a distinct vascularization strategy that enables tumors to secure blood supply under anti-angiogenic pressure, thereby promoting hypoxia resistance and metastatic dissemination [14]. In cervical cancer, VM has been associated with higher histological grade, lymph node metastasis, and shorter survival [15]. Mechanistically, VM is closely linked to EMT, a reversible program in which epithelial tumor cells acquire mesenchymal-like features and enhanced migratory capacity. Key EMT transcription factors, including Snail, Slug, and Twist, have been implicated in VM regulation, suggesting that overlapping molecular networks underlie these processes [16–18]. However, the upstream regulators coordinating VM and EMT in cervical cancer remain unclear.

High-throughput transcriptomic and single-cell RNA sequencing (scRNA-seq) analyses have begun to reveal the complexity of tumor ecosystems and signaling crosstalk. Our preliminary integrative analyses of TCGA–GTEx transcriptomic data and scRNA-seq datasets identified EPHB2 as a candidate molecule of interest in cervical cancer, characterized by elevated expression in tumor tissues, enrichment in malignant epithelial cells, and associations with EMT-related features and VM-associated signatures. Pathway enrichment highlighted “Transcriptional misregulation in cancer”, a pathway encompassing aberrant transcription factors, chromatin remodelers, and epigenetic regulators implicated in cancer cell dedifferentiation, stemness, and therapy resistance [19–21]. These findings suggested a potential link between EPHB2 and transcriptional alterations underlying EMT/VM-related malignant phenotypes. However, this relationship has not been functionally validated in cervical cancer.

Notably, the “Transcriptional misregulation in cancer” pathway has attracted increasing attention as a druggable vulnerability in oncology. Dysregulated transcription factors and chromatin regulators, once considered “undruggable,” are now being targeted through small molecules and epigenetic inhibitors, offering new therapeutic opportunities [22, 23]. In parallel, downstream oncogenic cascades such as RAS/ERK signaling, which may be activated in transcriptionally dysregulated tumor states, play important roles in sustaining EMT and VM phenotypes [24, 25]. Together, these observations provide a rationale for examining downstream pathway alterations associated with EPHB2 dysregulation in cervical cancer.

Beyond angiogenesis and EMT, the tumor immune microenvironment (TIME) represents another critical determinant of cervical cancer progression. Previous studies have shown that EMT and VM can modulate immune cell recruitment and contribute to an immunosuppressive milieu characterized by increased regulatory T cells (Tregs), tumor-associated macrophages (TAMs), and dysfunctional cytotoxic T lymphocytes [26–28]. In our preliminary transcriptomic analyses, EPHB2 expression was associated with selected immune-regulatory features, suggesting that immune-related programs may provide additional context for EPHB2-associated phenotypes in cervical cancer. Although not a central focus of the present study, these features help contextualize EPHB2-associated tumor biology.

In this study, we investigated the role of EPHB2 in cervical cancer progression using an integrated strategy combining bulk transcriptomic analysis, single-cell RNA-seq, in vitro functional assays, and in vivo validation. We evaluated EPHB2 expression, prognostic relevance, and associations with EMT- and VM-related features using TCGA–GTEx and scRNA-seq data. Gain- and loss-of-function experiments were performed to assess the effects of EPHB2 on proliferation, migration, apoptosis, EMT-associated molecular changes, and VM-like network formation. We further explored downstream transcriptional alterations associated with EPHB2 dysregulation, focusing on candidate signaling pathways identified from transcriptomic analyses and validated in xenograft models.

By integrating multi-omics analyses with in vitro and in vivo functional evidence, this study supports a role for EPHB2 in EMT- and VM-related phenotypes in cervical cancer and provides a mechanistic basis for further investigation of EPHB2-associated signaling programs.

## Materials and methods

### Public bulk transcriptomic data acquisition and analysis (TCGA–GTEx integration)

**Fig. 1:**
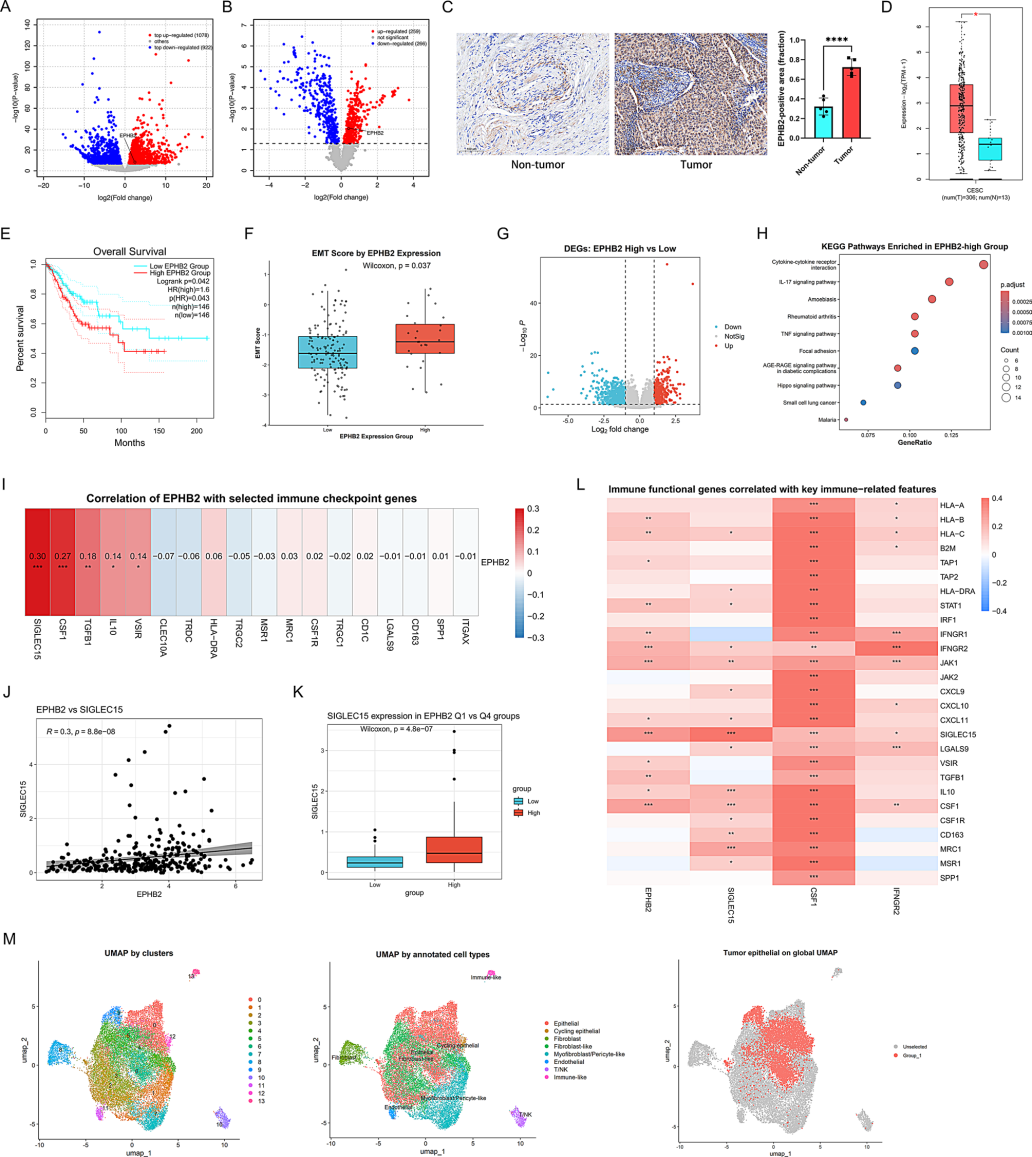

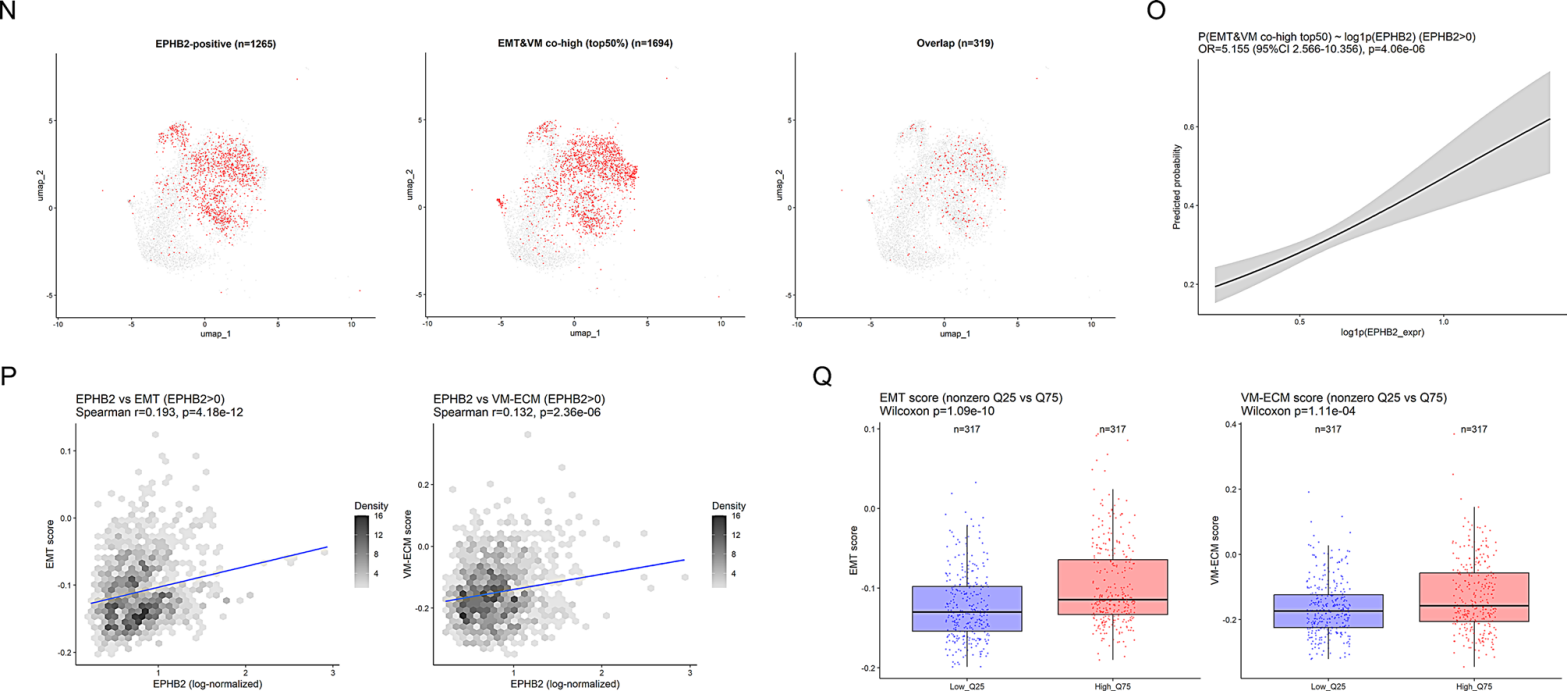
Multi-cohort transcriptomic profiling, clinical validation, immune-related analysis, and single-cell mapping identify EPHB2 upregulation and its association with immune-related features and EMT/VM-linked epithelial states in cervical cancer. (**A**) Volcano plot of differential gene expression between cervical cancer tissues and normal cervical tissues in GSE168244. The top 2000 most significant genes are highlighted, with EPHB2 indicated. (**B**) Volcano plot of the same top 2000 genes from (A) evaluated in an independent dataset (GSE138080) comparing precancerous lesions versus normal cervical tissues; EPHB2 remains differentially expressed and is highlighted. (**C**) Representative immunohistochemistry (IHC) images of EPHB2 in non-tumor and tumor cervical tissues, with quantification of the EPHB2-positive area fraction. Each dot represents one tissue section; the plotted value denotes the mean positive area fraction across randomly selected fields. Scale bars as indicated. (**D**) EPHB2 mRNA expression in tumor tissues from TCGA-CESC and normal cervical tissues from GTEx, obtained from the GEPIA2 web server. Expression is shown as log2(TPM + 1) (tumor *n* = 306; normal *n* = 13). (**E**) Kaplan–Meier overall survival analysis of TCGA-CESC patients stratified by EPHB2 expression (high vs. low). Hazard ratio (HR), 95% confidence interval (CI), and log-rank *P* value are shown in the panel. (**F**) Comparison of EMT scores between EPHB2-low and EPHB2-high TCGA-CESC groups. (**G**) Volcano plot of differentially expressed genes (DEGs) between EPHB2-high and EPHB2-low TCGA-CESC tumors; upregulated and downregulated genes are highlighted, with thresholds indicated by dashed lines. (**H**) KEGG pathway enrichment analysis of genes associated with high EPHB2 expression; dot size indicates gene count and color denotes pathway significance. (**I**) Correlation heatmap showing the association between EPHB2 and selected immune checkpoint/immunoregulatory genes in TCGA-CESC. (**J**) Scatter plot showing the correlation between EPHB2 and SIGLEC15 expression in TCGA-CESC. (**K**) Comparison of SIGLEC15 expression between the lowest (Q1) and highest (Q4) EPHB2 expression groups in TCGA-CESC. (**L**) Correlation heatmap of representative immune-related features (EPHB2, SIGLEC15, CSF1, and IFNGR2) with selected immune functional genes. (**M**) Single-cell RNA-seq analysis of GSE168652. Left, global UMAP clustering of all cells; middle, UMAP annotated by major cell types; right, localization of tumor epithelial cells on the global UMAP. (**N**) UMAP visualization of EPHB2-positive cells, EMT/VM co-high cells, and their overlap within the tumor epithelial compartment. (**O**) Logistic regression analysis showing the relationship between EPHB2 expression and the predicted probability of belonging to the EMT/VM co-high state. (**P**) Correlation analysis of EPHB2 expression with EMT score and VM-ECM score at the single-cell level. (**Q**) Comparison of EMT score and VM-ECM score between the low (Q25) and high (Q75) EPHB2 expression groups at the single-cell level. All statistical analyses are indicated in the corresponding panels; all *P* values are two-sided unless otherwise specified. Statistical analysis: Differential expression analyses in the GEO bulk transcriptomic datasets (**A**, **B**) were performed using GEO2R, whereas differential expression analysis between EPHB2-high and EPHB2-low TCGA-CESC tumors (**G**) was performed using the DESeq2 package. Volcano plots were generated to visualize ranked differentially expressed genes. Group comparisons in (**C**, **D**, **F**, **K**, **Q**) were conducted using two-sided Wilcoxon rank-sum tests unless otherwise indicated in the panel. Kaplan–Meier survival analysis (**E**) was evaluated using the log-rank (Mantel–Cox) test. KEGG enrichment analysis (H) was performed using over-representation analysis. Correlation analyses in (**I**, **J**, **L**, **P**) were performed using Spearman correlation unless otherwise specified in the panel. Logistic regression was used in (**O**) to model the probability of the EMT/VM co-high state as a function of EPHB2 expression

Bulk RNA-seq expression data and corresponding clinical information for cervical squamous cell carcinoma and endocervical adenocarcinoma (TCGA-CESC) were obtained from The Cancer Genome Atlas (TCGA). Normal cervical tissue expression profiles were derived from the Genotype-Tissue Expression (GTEx) project using a harmonized TCGA–GTEx resource to minimize cross-cohort processing differences. The comparative expression plot shown in Fig. 1D was generated using the GEPIA2 web server based on TCGA-CESC and GTEx normal cervix data, with gene expression analyzed as log2(TPM + 1). Differences in EPHB2 expression between tumor and normal tissues were assessed using a two-sided Wilcoxon rank-sum test. For survival analysis, TCGA-CESC patients were stratified into EPHB2-high and EPHB2-low groups using median expression as the cutoff, and overall survival was compared using the Kaplan–Meier method with the log-rank test. Hazard ratios (HRs) and 95% confidence intervals (CIs) were estimated using a univariate Cox proportional hazards model [29].

### Determination of optimal cutoff for downstream subgroup analysis

Patients in the TCGA-CESC cohort were stratified into EPHB2-high and EPHB2-low subgroups using the optimal expression cutoff determined by the surv_cutpoint function in the survminer R package, which identifies the threshold that best separates survival outcomes. This cutoff was applied specifically to downstream subgroup analyses, including EMT score comparison, differential expression analysis, and pathway enrichment.

### Differential expression and functional enrichment analysis

Within the TCGA-CESC cohort, patients were stratified into EPHB2-high and EPHB2-low groups using the optimal expression cutoff determined by the surv_cutpoint function in the survminer R package. Differentially expressed genes (DEGs) between these predefined groups were identified using the DESeq2 package, with thresholds of |log₂ fold change (FC)| > 1 and adjusted *P* < 0.05 after Benjamini–Hochberg correction [30]. Volcano plots and hierarchical clustering heatmaps were generated to visualize DEGs. Functional enrichment analyses, including Gene Ontology (GO) biological processes and Kyoto Encyclopedia of Genes and Genomes (KEGG) pathways, were performed using clusterProfiler [31]. Tumor progression-related pathways and biological programs, including “Transcriptional misregulation in cancer”, epithelial–mesenchymal transition (EMT), and vasculogenic mimicry (VM), were prioritized for biological interpretation and downstream validation.

### Immune-related transcriptomic analysis

Immune-related transcriptomic analyses were performed in the TCGA-CESC cohort. Associations between EPHB2 expression and selected immune checkpoint/immunoregulatory genes were evaluated using Spearman correlation, with correlation coefficients visualized as a heatmap. The relationship between EPHB2 and SIGLEC15 expression was examined using scatter plots and Spearman correlation analysis. SIGLEC15 expression was compared between the lowest (Q1) and highest (Q4) EPHB2 expression groups using the Wilcoxon rank-sum test. Representative immune-related features were then correlated with a panel of immune functional genes using Spearman correlation, and the results were displayed as a heatmap.

### Single-cell RNA-seq processing and epithelial-state analysis

Publicly available single-cell RNA-seq data from cervical cancer tissues were analyzed using Seurat (R package) [32]. Low-quality cells were excluded according to standard quality-control criteria, including low numbers of detected genes, excessive gene counts suggestive of multiplets, or high mitochondrial transcript percentages [33]. After quality control, data were normalized and scaled, highly variable genes were identified, and principal component analysis (PCA) was performed for dimensionality reduction. Graph-based clustering was conducted using the top principal components, and UMAP was used for visualization [34]. For samples derived from multiple individuals or batches, batch effects were assessed and corrected using the Seurat integration workflow.

Major cell types were annotated based on canonical marker genes. Tumor epithelial cells were identified and projected onto the global UMAP for subsequent analyses. To assess the association between EPHB2 expression and EMT/VM-related epithelial states, epithelial cells with detectable EPHB2 expression were defined as EPHB2-positive cells. EMT/VM co-high cells were defined as epithelial cells with both EMT and VM-ECM scores in the top 50% of their respective distributions. EPHB2-positive cells, EMT/VM co-high cells, and overlapping cells were visualized on UMAPs to examine their spatial distribution.

To quantify these associations, logistic regression was used to model the probability of belonging to the EMT/VM co-high state as a function of EPHB2 expression. Spearman correlation analysis was used to evaluate the relationships between EPHB2 expression and EMT or VM-ECM scores in epithelial cells with nonzero EPHB2 expression. For subgroup comparisons, epithelial cells were stratified by EPHB2 expression quartiles, and EMT and VM-ECM scores were compared between the low-expression (Q25) and high-expression (Q75) groups using the Wilcoxon rank-sum test.

### Cell lines and culture conditions

The human cervical cancer cell line HeLa (Catalog No. FH0314) and the human cervical squamous cell carcinoma cell line SiHa (Catalog No. FH0309) were purchased from FuHeng Cell Center (Shanghai, China) and used as cervical cancer cell models in this study. Both cell lines were authenticated by the supplier using short tandem repeat (STR) profiling and confirmed to be mycoplasma-free before experimental use, including in vivo implantation. Cells were maintained at low passage numbers and cultured under standard conditions (37 °C, 5% CO₂) in a humidified incubator. In all in vitro assays, the “control” group refers to the negative control condition within the same cell line, such as Si-NC or empty vector, rather than a nonmalignant cell type or a different cell line.

### Gene knockdown, overexpression, and MEK/ERK pathway inhibition

Transient and stable approaches were used to modulate EPHB2 and ETV4 expression in cervical cancer cell lines.

For transient gene silencing, small interfering RNAs (siRNAs) targeting EPHB2 or ETV4, together with a non-targeting negative control siRNA (Si-NC), were transfected into cells using a lipid-based transfection reagent according to the manufacturer’s instructions. Cells were harvested 48 h after transfection, and knockdown efficiency was evaluated at the mRNA and protein levels by quantitative real-time PCR (qRT-PCR) and Western blotting. The sense and antisense sequences of all siRNAs are listed in Table 1.

For stable EPHB2 knockdown, lentiviral short hairpin RNA (shRNA) constructs targeting different regions of the EPHB2 transcript, designated sh-1, sh-2, and sh-3, were generated and packaged into lentiviral particles. Cervical cancer cells were infected with the indicated lentiviruses and selected with puromycin to establish stable knockdown cell pools. The silencing efficiencies of individual shRNA constructs were compared by qRT-PCR, and the construct with the highest knockdown efficiency, sh-3, was selected for in vivo experiments. A non-targeting shRNA (sh-NC) was used as the negative control for lentiviral knockdown assays. The target and antisense sequences of the shRNA constructs are provided in Table 1.

For gain-of-function experiments, cells were transfected with an overexpression plasmid encoding full-length human EPHB2 (pcMV3-SV40-EGFP-2 A-puro-C3-Flag-EPHB2) or the corresponding empty vector control (pcMV3-SV40-EGFP-2 A-puro-CMV-NC) using the same transfection protocol. Overexpression efficiency was confirmed by qRT-PCR and Western blotting before functional analyses.

For MEK/ERK pathway inhibition, EPHB2-overexpressing HeLa cells were treated with the MEK inhibitor U0126 at 10 µM for 48 h before Western blot analysis, with DMSO-treated cells used as vehicle controls.

**Table 1:** Sequences of siRNAs and shRNAs targeting EPHB2 and ETV4 (5′→3′)

Target gene	RNA ID	Sense (5′→3′)	Antisense (5′→3′)	Length (nt)
EPHB2	Si-1	ACCUCGUCUACAACAUCAUTT	AUGAUGUUGUAGACGAGGUTT	21
EPHB2	Si-2	UGAACAGUAUCCAGGUGAUTT	AUCACCUGGAUACUGUUCATT	21
EPHB2	Si-3	AGAAGGAGCUCAGUGAGUATT	UACUCACUGAGCUCCUUCUTT	21
ETV4	Si-1	CCGAUACUAUUAUGAGAAATT	UUUCUCAUAAUAGUAUCGGTT	21
ETV4	Si-2	CUGCGUUGUCCCUGAGAAATT	UUUCUCAGGGACAACGCAGTT	21
ETV4	Si-3	GAAUGGAGUUCAAGCUCAUTT	AUGAGCUUGAACUCCAUUCTT	21
Negative control	Si-NC	UUCUCCGAACGUGUCACGUTT	ACGUGACACGUUCGGAGAATT	21
EPHB2	sh-1	GCTAGACAAGATGATCCGCAA	TTGCGGATCATCTTGTCTAGC	21
EPHB2	sh-2	GCTGTGATTTCCAGTGTCAAT	ATTGACACTGGAAATCACAGC	21
EPHB2	sh-3	CGGGAGTTTGCCAAGGAAATT	AATTTCCTTGGCAAACTCCCG	21
Negative control	sh-NC	non-targeting	non-targeting	-

### Quantitative real-time PCR (qRT-PCR)

Total RNA was extracted using TRIzol reagent, and reverse transcription was performed using a standard cDNA synthesis kit. qRT-PCR was conducted using SYBR Green chemistry on a real-time PCR system. Relative gene expression was calculated using the 2⁻ΔΔCt method, with β-actin (ACTB) serving as the internal control. The primer sequences used are listed in Table 2.

**Table 2:** Primer sequences used for qRT-PCR (5′→3′)

Gene	Forward primer (5′→3′)	Reverse primer (5′→3′)
EPHB2	AGAAACGCTAATGGACTCCACT	GTGCGGATCGTGTTCATGTT
ETV4	GAAACCTCTGCGACCATTCC	AGCAAGGCCACCAGAAATTG
ETV5	GCTGTCGTCTTGTAGCCATG	GGATTCTGATGGGTGGGTGA
DUSP6	GACCGAGACCCCAATAGTGC	GGGGGTGACGTTCAAGATGT
CXCL8	CAGTTTTGCCAAGGAGTGCT	ACTTCTCCACAACCCTCTGC
PI3K	AGCTGGTTTGGATCTTCGGA	CAGGTCATCCCCAGAGTTGT
KRAS	ACACAAAACAGGCTCAGGAC	TCACACAGCCAGGAGTCTTT
ERK1	TCAACACCACCTGCGACCTTAA	GTACCAGCGCGTAGCCACATA
ERK2	CTAACGTTCTGCACCGTGAC	AGAATGCAGCCTACAGACCA
β-actin (ACTB)	CACCATTGGCAATGAGCGGTTC	AGGTCTTTGCGGATGTCCACGT

### RNA sequencing and differential expression analysis after EPHB2 knockdown

HeLa cells were transfected with control siRNA (Si-NC) or two independent EPHB2 siRNAs (Si-2 and Si-3) and harvested 48 h post-transfection. Total RNA was extracted using TRIzol reagent according to the manufacturer’s instructions. Poly(A) + RNA libraries were constructed using the VAHTS Universal V6 RNA-seq Library Prep Kit for Illumina together with VAHTS mRNA Capture Beads (Human/Mouse/Rat), followed by purification with VAHTS DNA Clean Beads. Library quality control was performed using Agilent D1000 reagents. The RNA-seq data generated in this study have been deposited in the Gene Expression Omnibus (GEO) under accession number GSE326407.

Raw reads were processed to remove adapters and low-quality bases using trimadap and seqtk trimfq, and short fragments were filtered using lengthsort.pl. rRNA-derived reads were removed by alignment to rRNA reference sequences using Bowtie2. Read quality was assessed with FastQC. Clean reads were aligned to the human reference genome (GRCh38) using HISAT2, and SAM/BAM files were processed using SAMtools and Sambamba. Transcript abundance was quantified using StringTie. Differential expression analyses were performed using edgeR with three biological replicates per group (*n* = 3). Differentially expressed genes were identified using a predefined adaptive filtering strategy: genes with q-value ≤ 0.05 and |log₂ fold change| ≥ 1 were selected as DEGs; when fewer than 50 DEGs were obtained in a comparison, the statistical threshold was automatically adjusted to nominal P-value ≤ 0.05 while retaining the |log₂ fold change| ≥ 1 criterion. Accordingly, for the Si-2 vs. Si-NC and Si-3 vs. Si-NC comparisons, the final gene sets used for candidate screening and pathway enrichment were defined using P-value ≤ 0.05 and |log₂ fold change| ≥ 1. Key candidates were subsequently validated by qRT–PCR and immunoblotting.

KEGG pathway enrichment was performed using these final DEG sets, followed by Benjamini–Hochberg correction at the pathway-enrichment level. To prioritize robust downstream signals, enrichment results from the two independent knockdown datasets were compared, and pathways consistently appearing among the top 15 enriched terms in both comparisons were selected for mechanistic follow-up. For visualization, the top 15 enriched KEGG pathways ranked by adjusted P value were displayed for each comparison.

### Western blotting

Proteins were extracted using lysis buffer supplemented with protease and phosphatase inhibitors, quantified, and separated by SDS-PAGE. After transfer to PVDF membranes, blots were blocked and incubated with primary antibodies overnight at 4 °C, followed by HRP-conjugated secondary antibodies. Protein bands were visualized using enhanced chemiluminescence, and relative band intensities were quantified using image analysis software.

### Cell proliferation assay

Cell viability was measured using a colorimetric assay. Transfected cells were seeded in 96-well plates at 2 × 10³ cells/well, and absorbance at 450 nm was recorded at 24, 48, 72, and 96 h.

### Colony formation assay

For colony formation assays, cells were seeded in 6-well plates at 1,000 cells per well. For knockdown experiments, HeLa and SiHa cells were cultured for 10 and 14 days, respectively; for overexpression experiments, HeLa and SiHa cells were cultured for 7 and 10 days, respectively. At the endpoint, colonies were fixed with paraformaldehyde, stained with crystal violet, imaged, and quantified using image analysis software.

### Wound healing assay

Cells were seeded in 6-well plates and grown to near confluence. A linear scratch was created using a sterile pipette tip, and detached cells were removed by washing. For migration assessment, HeLa cells were imaged at 0 and 48 h, whereas SiHa cells were imaged at 0 and 60 h. Wound closure was quantified relative to the initial wound width using image analysis software.

### Transwell migration assay

Migration assays were performed using uncoated Transwell chambers. Cells were seeded in serum-free medium in the upper chamber and allowed to migrate toward chemoattractant-containing medium in the lower chamber. After incubation, migrated cells were fixed, stained, and counted.

### Flow cytometry analysis

Flow cytometry was performed specifically in EPHB2-knockdown cells to assess apoptosis following EPHB2 silencing.

For apoptosis analysis, cervical cancer cells transfected with EPHB2-targeting siRNAs or the corresponding negative control (Si-NC) were harvested 48 h post-transfection, washed twice with cold phosphate-buffered saline (PBS), and stained with Annexin V–FITC and propidium iodide (PI) according to the manufacturer’s instructions. Briefly, cells were resuspended in binding buffer and incubated with Annexin V–FITC and PI in the dark at room temperature for 15 min. Stained cells were then analyzed by flow cytometry, and the proportions of viable, early apoptotic, late apoptotic, and necrotic cells were quantified.

All flow cytometric analyses were performed under identical instrument settings, with at least 10,000 events acquired per sample. Flow cytometric data were analyzed using FlowJo software.

### TUNEL staining assay

A terminal deoxynucleotidyl transferase–mediated dUTP nick-end labeling (TUNEL) assay was performed exclusively in vitro in EPHB2-overexpressing cells to evaluate apoptosis-associated DNA fragmentation.

Cells transfected with the EPHB2 overexpression plasmid or the corresponding empty vector control were seeded on coverslips and allowed to adhere. Cells were then fixed with paraformaldehyde, permeabilized, and subjected to TUNEL staining according to the manufacturer’s protocol. TUNEL-positive nuclei were visualized using a fluorescence microscope. Nuclei were counterstained with DAPI, and representative images were acquired under identical exposure settings.

The apoptotic index was calculated as the percentage of TUNEL-positive nuclei relative to the total number of DAPI-stained nuclei in at least five randomly selected fields.

### Vasculogenic mimicry (VM) assay

Vasculogenic mimicry (VM) formation was evaluated using a Matrigel-based tube formation assay. Briefly, Matrigel was added to 24-well plates at 300 µL per well and allowed to polymerize at 37 °C for 30 min. At 48 h after transfection, cells were harvested, counted, and assessed for viability. Equal numbers of viable cells were seeded onto Matrigel-coated wells across all experimental groups to ensure identical initial cell density (2 × 10⁵ cells/mL; 1 mL per well). Tube-like structures were imaged 8 h after plating. Total tube length, junction number, and mesh number were quantified using ImageJ with the Angiogenesis Analyzer plugin. All experiments were performed in at least three independent biological replicates.

### Immunohistochemistry (IHC)

Immunohistochemistry was performed on paraffin-embedded cervical tissue sections. Briefly, sections were deparaffinized, rehydrated, and subjected to antigen retrieval according to standard protocols, followed by blocking of endogenous peroxidase activity and nonspecific binding. Sections were then incubated with primary antibodies against EPHB2 and selected EMT- and VM-related markers, followed by appropriate secondary antibodies. Immunoreactivity was visualized using 3,3′-diaminobenzidine (DAB), and sections were counterstained with hematoxylin.

For quantitative analysis of EPHB2 staining, at least five randomly selected high-power fields (×400) were captured from each tissue section. The DAB-positive area was measured using ImageJ software, and the positive area fraction was calculated as the ratio of DAB-positive area to the total analyzed area in each field. For each section, the final value was defined as the mean positive area fraction across all evaluated fields, and these section-level values were used for statistical analysis. Image acquisition and analysis were performed in a blinded manner by investigators unaware of the clinicopathological information.

### In vivo animal studies

Male BALB/c nude mice (4–6 weeks old) were used for xenograft experiments. Mice were randomly assigned to two groups and subcutaneously injected in the flank with SiHa cells stably expressing sh-NC or sh-EPHB2 (5 × 10⁶ cells in 100 µL PBS). All mice received the same inoculation dose under identical experimental conditions. Tumor growth was monitored every 3 days by measuring tumor length and width with a digital caliper, and tumor volume was calculated using the following formula:

$$\:Volume=length\times\:{\left(width\right)}^{2}\times\:0.52$$

Tumor measurements were performed in a blinded manner to minimize observer bias. After four weeks, mice were euthanized, and tumors were excised, weighed, and photographed. Tumor tissues were fixed in formalin and embedded in paraffin for histological and immunohistochemical analyses, including hematoxylin and eosin (H&E) staining, CD31/PAS double staining to assess vascular/VM-associated histologic features, and IHC for relevant markers. All procedures were conducted in accordance with institutional animal care guidelines and approved by the Animal Ethics Committee.

### Statistical analysis

All data analyses were performed using R software (v4.3.1) and GraphPad Prism (v9.0). Quantitative data are presented as the mean ± standard deviation (SD) from at least three independent experiments unless otherwise indicated. Comparisons between two groups were performed using an unpaired two-tailed Student’s t-test for parametric data or the Wilcoxon rank-sum test for non-parametric data. For multiple group comparisons, one-way ANOVA followed by Tukey’s post hoc test was applied. Survival differences were assessed using the Kaplan–Meier method with the log-rank test. Correlations were evaluated using Spearman’s rank correlation coefficient. Immunohistochemical positive area fraction data are presented as median (range). A two-tailed P value < 0.05 was considered statistically significant.

## Results

### EPHB2 is upregulated in cervical cancer and is associated with immune-related features and EMT/VM-linked epithelial states

To define the expression pattern and biological relevance of EPHB2 in cervical cancer, we performed a multi-level evaluation integrating public bulk transcriptomic datasets, institutional immunohistochemistry (IHC), TCGA/GTEx analysis, and single-cell RNA-seq, with particular attention to EMT/VM-related epithelial states and immune-related transcriptional features.

At the public transcriptomic level, we first analyzed cervical cancer-related GEO expression datasets. In the discovery cohort GSE168244, differential expression analysis between cervical cancer and normal cervical tissues identified a broad set of dysregulated genes. Volcano plot visualization showed that EPHB2 was among the upregulated genes in tumor tissues, suggesting its potential involvement in molecular processes related to cervical carcinogenesis (Fig. 1A). To assess the robustness of this observation, we extracted the top 2000 differentially expressed genes ranked by P value in GSE168244 and examined them in the independent validation cohort GSE138080, which compares precancerous lesions with normal cervical tissues. EPHB2 remained differentially expressed in this cohort (Fig. 1B). Together, these findings indicate that aberrant EPHB2 expression is not restricted to invasive cancer but may already emerge during premalignant progression.

At the clinical tissue level, we performed EPHB2 IHC in non-tumor cervical tissues and cervical squamous cell carcinoma samples to validate its protein-level expression. The clinicopathological characteristics of these samples are summarized in Table 3. Representative staining showed generally weak EPHB2 expression in non-tumor cervical tissues, with only limited faint staining, whereas cervical squamous cell carcinoma tissues displayed stronger and more extensive brown-positive staining (Fig. 1C). Quantitative analysis based on section-level positive area fraction confirmed significantly higher EPHB2 protein expression in tumor tissues than in non-tumor controls, with median EPHB2-positive area fractions of 0.71 (0.63–0.82) and 0.30 (0.22–0.43), respectively (Fig. 1C; Table 3). This protein-level increase was concordant with the GEO transcriptomic findings and further supported aberrant EPHB2 upregulation in cervical cancer.

To evaluate EPHB2 expression in a larger public cohort, we integrated TCGA-CESC tumor tissues with GTEx normal cervical tissues. EPHB2 mRNA expression was significantly higher in tumor tissues than in normal tissues, further supporting transcript-level upregulation in cervical cancer (Fig. 1D). TCGA-CESC patients were then stratified into EPHB2-high and EPHB2-low groups using median EPHB2 expression as the cutoff for Kaplan–Meier survival analysis. Patients with high EPHB2 expression showed significantly worse overall survival, indicating that elevated EPHB2 is associated with adverse outcome in cervical cancer (Fig. 1E).

Using the surv_cutpoint-defined EPHB2-high and EPHB2-low subgroups within TCGA-CESC, we next examined the relationship between EPHB2 expression and EMT-related transcriptional features. The EPHB2-high group displayed a significantly higher EMT score than the EPHB2-low group (Fig. 1F). Differential expression analysis between the two groups revealed broad transcriptomic differences, indicating that high EPHB2 expression was accompanied by wider transcriptional reprogramming rather than isolated single-gene changes (Fig. 1G). KEGG enrichment analysis of the differentially expressed genes identified pathways including cytokine–cytokine receptor interaction, IL-17 signaling, TNF signaling, focal adhesion, and Hippo signaling (Fig. 1H). These pathways are closely related to inflammatory signaling, cell adhesion, tumor–microenvironment interactions, and malignant progression, suggesting that high EPHB2 expression may be linked to remodeling of cervical cancer-associated molecular networks.

After establishing that EPHB2 is upregulated in cervical cancer and associated with worse overall survival and EMT-related transcriptional features, we explored its relationship with immune-related molecules in TCGA-CESC. Correlation heatmap analysis showed variable associations between EPHB2 and multiple immune checkpoint or immunoregulatory genes (Fig. 1I). Among these candidates, stronger positive correlations were observed with SIGLEC15, CSF1, IL10, and VSIR, whereas associations with several other molecules were weaker or less evident. Overall, EPHB2 expression tended to vary in the same direction as genes linked to immunoregulatory and myeloid-associated suppressive features. Given the notable association with SIGLEC15, we further examined this relationship. Scatter plot analysis demonstrated a significant positive correlation between EPHB2 and SIGLEC15 expression (*R* = 0.30, *P* = 8.8 × 10⁻⁸) (Fig. 1J). Consistently, SIGLEC15 expression was significantly higher in the highest EPHB2 expression group than in the lowest expression group (Fig. 1K).

Based on these results, we selected EPHB2, SIGLEC15, CSF1, and IFNGR2 as representative immune-related features and evaluated their correlations with a panel of immune functional genes (Fig. 1L). EPHB2 was included as the core molecule of interest; SIGLEC15 represented checkpoint-related immunoregulatory features; CSF1 reflected myeloid recruitment/differentiation-associated programs; and IFNGR2 served as a marker of interferon-response signaling. The resulting heatmap showed that all four features were associated with immune functional genes to varying degrees, with CSF1 displaying the broadest correlation pattern. These observations suggest that high EPHB2 expression is associated with an immune-regulatory transcriptional context in cervical cancer, particularly one linked to immunoregulatory, myeloid-related, and inflammatory-response features. However, these analyses remain correlative and do not establish that EPHB2 directly shapes the tumor immune microenvironment.

To further investigate the relationship between EPHB2 and EMT/VM-related states at the level of cellular heterogeneity, we analyzed the publicly available cervical cancer single-cell RNA-seq dataset GSE168652. UMAP visualization revealed multiple distinct cell clusters (Fig. 1M, left). Based on canonical marker expression, major cell populations were annotated as epithelial cells, cycling epithelial cells, fibroblasts, fibroblast-like cells, myofibroblast/pericyte-like cells, endothelial cells, T/NK cells, and immune-like cells, indicating marked cellular complexity and heterogeneity within cervical cancer tissues (Fig. 1M, middle). Mapping tumor epithelial cells onto the global UMAP showed that they were mainly distributed in the central region of the atlas, providing the basis for subsequent analysis of intratumoral epithelial-state variation (Fig. 1M, right).

Within the tumor epithelial compartment, EPHB2-positive cells and EMT/VM co-high cells showed partial overlap on the UMAP and were both enriched in the core epithelial region, suggesting a potential correspondence between EPHB2 expression and EMT/VM-related epithelial states (Fig. 1N). Logistic regression analysis showed that the predicted probability of belonging to the EMT/VM co-high state increased with EPHB2 expression (Fig. 1O). In addition, among cells with nonzero EPHB2 expression, EPHB2 levels were positively correlated with both EMT and VM-ECM scores (Fig. 1P). Consistently, cells in the high EPHB2 expression quartile showed significantly higher EMT and VM-ECM scores than those in the low-expression quartile (Fig. 1Q). These single-cell results support a coordinated relationship between high EPHB2 expression and EMT/VM-related epithelial states in cervical cancer at cellular resolution.

Collectively, evidence from GEO cohorts, institutional IHC validation, TCGA/GTEx analysis, immune-related transcriptomic profiling, and single-cell RNA-seq consistently indicates that EPHB2 is aberrantly upregulated in cervical cancer. High EPHB2 expression was associated with worse overall survival, immune-related transcriptional features, and EMT/VM-linked malignant epithelial states, providing a rationale for subsequent mechanistic investigation of EPHB2 in cervical cancer progression.

**Table 3:** Clinicopathological characteristics of the cervical tissue samples used for EPHB2 immunohistochemistry

Variable	Non-tumor (*n* = 5)	Tumor (*n* = 5)
Age (years), median (range)	53 (47–58)	55 (51–62)
Tissue type	Non-tumor cervical tissue	Cervical cancer
Histological type	-	Squamous cell carcinoma
FIGO stage	-	I (*n* = 2), II (*n* = 3)
EPHB2-positive area fraction, median (range)	0.30 (0.22–0.43)	0.71 (0.63–0.82)

### EPHB2 knockdown suppresses malignant phenotypes and partially reverses EMT in cervical cancer cells

**Fig. 2:**
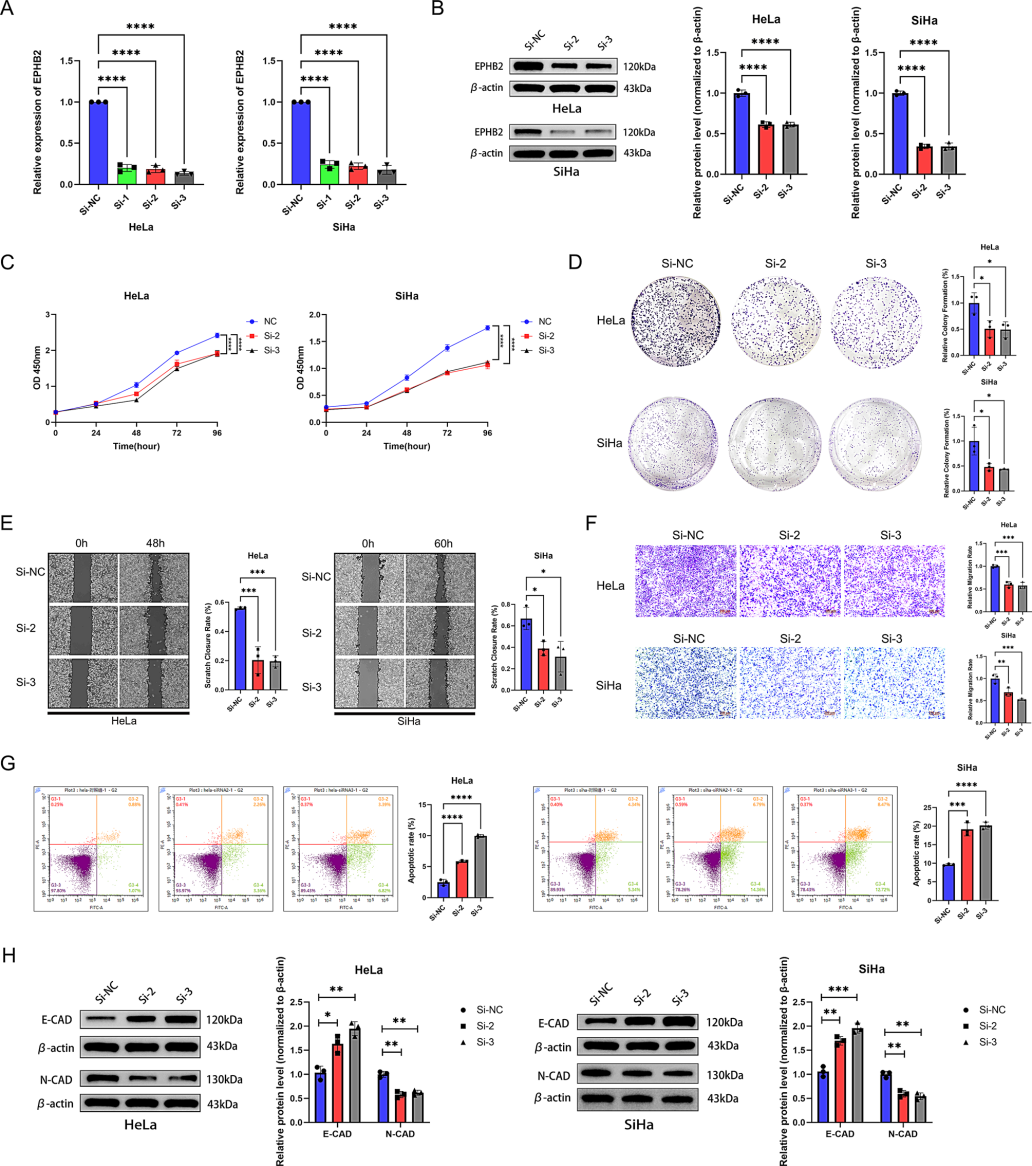
Knockdown of EPHB2 impairs proliferation and migration, induces apoptosis, and partially reverses EMT in cervical cancer cells. (**A**) qRT-PCR showing effective suppression of EPHB2 expression in HeLa and SiHa cells transfected with Si-2 or Si-3. (**B**) Western blot confirming the reduction of EPHB2 protein levels in both cell lines; β-actin served as the loading control. Quantification is shown on the right. (**C**) CCK-8 assays demonstrating that EPHB2 silencing inhibits cell proliferation over a 96-hour period. (**D**) Colony formation assays showing reduced clonogenic capacity following EPHB2 knockdown. Representative images and quantification are presented. (**E**) Wound-healing assays revealing impaired migratory ability in EPHB2-deficient cells at the indicated time points. (**F**) Transwell migration assays further confirming the decreased motility of HeLa and SiHa cells after EPHB2 knockdown. (**G**) Annexin V–FITC/PI flow cytometry showing increased apoptosis in EPHB2-silenced cells. Quantified apoptotic rates are shown on the right. (**H**) Western blot analysis of EMT markers demonstrating upregulation of E-cadherin and downregulation of N-cadherin after EPHB2 knockdown. Densitometric quantification normalized to β-actin is provided. Quantitative data are presented as mean ± SD from at least three independent experiments. Panels A, B, D, E, F, G, and H were analyzed using one-way ANOVA followed by Tukey’s post hoc test. Panel C (CCK-8 time-course assay) was analyzed using two-way ANOVA. *P < 0.05; **P < 0.01; ***P < 0.001; ****P < 0.0001

To determine the functional contribution of EPHB2 in cervical cancer cells, we performed siRNA-mediated knockdown in HeLa and SiHa cells. Three candidate siRNAs (Si-1, Si-2, and Si-3) were first evaluated by qRT–PCR to identify effective knockdown sequences. All three reduced EPHB2 transcript levels relative to the negative control (Si-NC), with Si-2 and Si-3 producing the most pronounced and consistent suppression in both cell lines; therefore, these two siRNAs were selected for subsequent functional experiments (Fig. 2A). Western blotting confirmed that Si-2 and Si-3 markedly decreased EPHB2 protein levels in HeLa and SiHa cells, with β-actin used as the loading control and densitometric quantification supporting the reduction (Fig. 2B).

We next examined whether EPHB2 depletion affects proliferative capacity. CCK-8 assays over a 96-hour time course showed that EPHB2-silenced cells proliferated significantly more slowly than control cells, with growth-curve divergence becoming increasingly evident over time in both HeLa and SiHa cells (Fig. 2C). Concordantly, colony formation assays demonstrated reduced clonogenic potential after EPHB2 knockdown, as reflected by a marked decrease in colony numbers compared with Si-NC (Fig. 2D). These results indicate that EPHB2 supports both short-term proliferation and long-term outgrowth.

Given the association of EPHB2 with aggressive transcriptional programs, we assessed cell motility after knockdown. In wound-healing assays, EPHB2-deficient cells showed delayed scratch closure at the indicated time points relative to controls, indicating impaired migratory ability (Fig. 2E). This finding was independently supported by Transwell migration assays, in which EPHB2 silencing significantly reduced the number of cells migrating through the membrane in both cell lines (Fig. 2F). Together, these data indicate that EPHB2 contributes to cervical cancer cell motility.

We further evaluated the impact of EPHB2 knockdown on cell survival. Annexin V–FITC/PI flow cytometry revealed increased apoptotic populations in EPHB2-silenced cells compared with Si-NC, with quantification confirming significantly elevated apoptotic rates in both HeLa and SiHa cells (Fig. 2G). Finally, consistent with the bioinformatic link between EPHB2 and EMT programs, western blot analysis of EMT markers showed increased E-cadherin and decreased N-cadherin after EPHB2 depletion, with densitometric values normalized to β-actin (Fig. 2H), indicating a partial reversal of EMT-associated marker patterns.

Collectively, these results indicate that EPHB2 helps maintain proliferative and clonogenic growth, promotes migratory behavior, suppresses apoptosis, and sustains EMT-related molecular features in cervical cancer cells (Fig. 2A–H). Data are presented as mean ± SD from at least three independent experiments. Statistical analyses were performed as indicated in the figure legends.

### EPHB2 overexpression promotes malignant phenotypes and enhances EMT-related features in cervical cancer cells

**Fig. 3:**
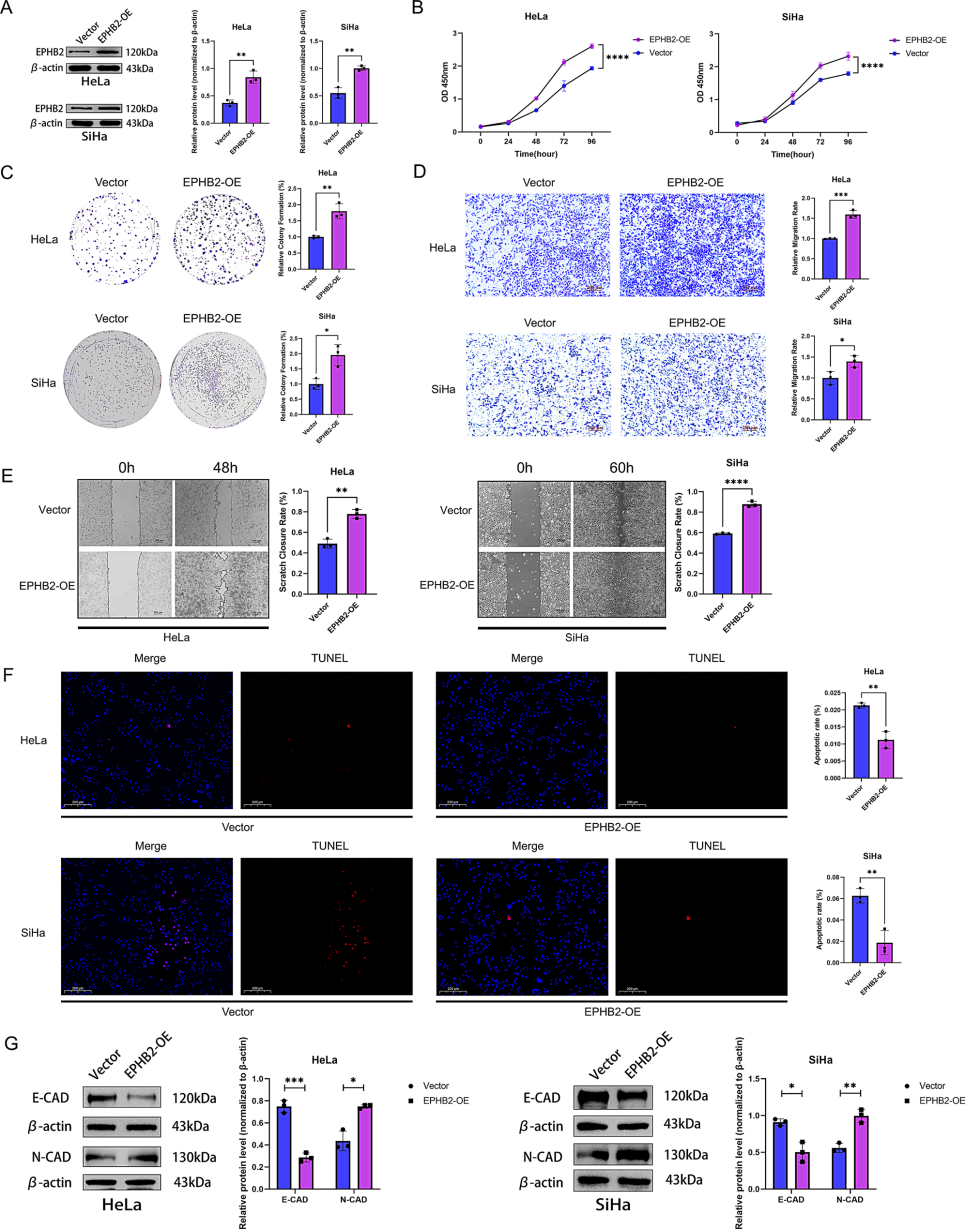
Overexpression of EPHB2 enhances proliferation, migration, and survival of cervical cancer cells. (**A**) Western blot validation of EPHB2 overexpression in HeLa and SiHa cells. β-actin was used as a loading control, and densitometric quantification is shown on the right. (**B**) CCK-8 assays showing accelerated cell growth in EPHB2-overexpressing cells over a 96-hour period. (**C**) Colony formation assays demonstrating enhanced clonogenic capacity in both cell lines after EPHB2 overexpression. (**D**) Transwell migration assays showing increased cell motility in EPHB2-overexpressing cells. Representative images and quantification are presented. (**E**) Wound-healing assays indicating faster closure rates in EPHB2-overexpressing cells at the indicated time points. (**F**) TUNEL staining showing reduced apoptosis in EPHB2-overexpressing HeLa and SiHa cells. Representative images (Merge and TUNEL) and quantification of TUNEL-positive cells are provided. (**G**) Western blot analysis of EMT markers demonstrating downregulation of E-cadherin and upregulation of N-cadherin after EPHB2 overexpression. Densitometric quantification normalized to β-actin is provided. Quantitative data are presented as mean ± SD from at least three independent experiments. Panel B (CCK-8 time-course assay) was analyzed using two-way ANOVA. Panels A, C, D, E, F, and G were analyzed using unpaired two-tailed Student’s t test. *P < 0.05; **P < 0.01; ***P < 0.001; ****P < 0.0001

To complement the loss-of-function findings, we established gain-of-function models by ectopically overexpressing EPHB2 in HeLa and SiHa cells. Western blotting confirmed a marked increase in EPHB2 protein abundance in EPHB2-overexpressing cells compared with vector controls, with densitometric quantification shown alongside the blots (Fig. 3A). These data validated the EPHB2-overexpressing systems for downstream functional analyses.

We first examined whether EPHB2 elevation influences cell growth. CCK-8 assays over a 96-hour time course showed accelerated proliferation in EPHB2-overexpressing cells relative to vector controls in both cell lines, with differences in OD values becoming more evident at later time points (Fig. 3B). Consistently, colony formation assays revealed enhanced clonogenic capacity after EPHB2 overexpression, as reflected by increased colony formation rates in both HeLa and SiHa cells (Fig. 3C). Together, these data indicate that EPHB2 overexpression promotes sustained growth and long-term proliferative outgrowth.

Given the association of EPHB2 with aggressive behavior, we next assessed cell motility. In Transwell migration assays, EPHB2-overexpressing cells exhibited a significantly higher number of migrated cells than vector controls (Fig. 3D). This pro-migratory phenotype was further supported by wound-healing assays, in which EPHB2-overexpressing cells closed the scratch gap more rapidly than control cells at the indicated time points (Fig. 3E), supporting enhanced migratory capacity across complementary assays.

Finally, we evaluated whether EPHB2 overexpression affects apoptosis. TUNEL staining showed fewer TUNEL-positive nuclei in EPHB2-overexpressing HeLa and SiHa cells than in vector controls, and quantification confirmed a significant reduction in apoptotic rates (Fig. 3F). To determine whether EPHB2 overexpression also induces reciprocal EMT-related molecular changes, we examined EMT markers by western blotting. EPHB2-overexpressing cells showed reduced E-cadherin and increased N-cadherin expression relative to vector controls in both HeLa and SiHa cells, with densitometric quantification normalized to β-actin supporting these changes (Fig. 3G). Collectively, these gain-of-function results mirror the phenotypes observed upon EPHB2 knockdown and support a role for EPHB2 in promoting proliferation and migration while enhancing survival and reinforcing EMT-related features in cervical cancer cells (Fig. 3A–G).

Data are presented as mean ± SD from at least three independent experiments. Statistical comparisons were performed using Student’s t-test or one-way ANOVA, as appropriate, with significance levels indicated in the corresponding panels.

### EPHB2 promotes VM-like capillary network formation in cervical cancer cells

**Fig. 4:**
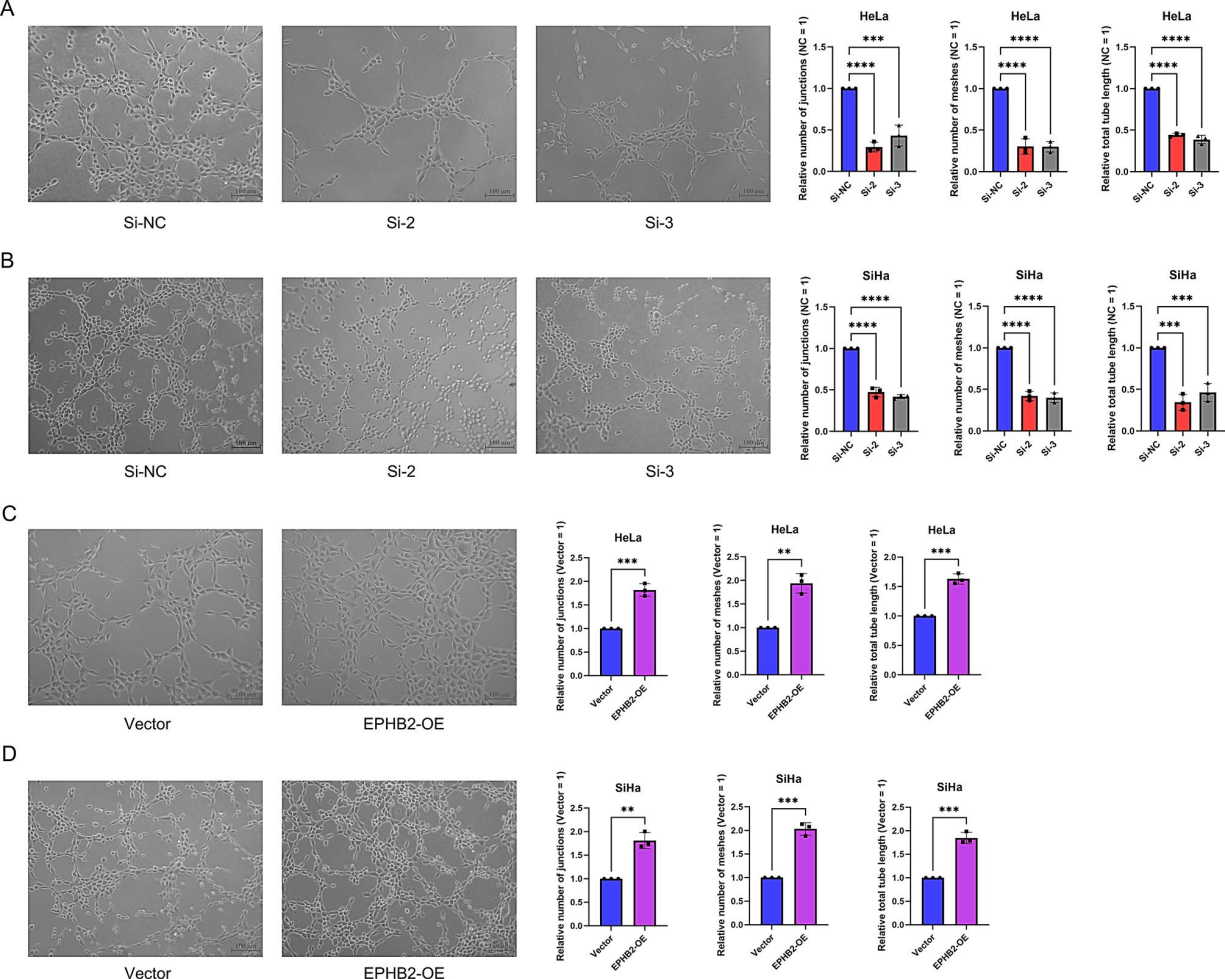
EPHB2 enhances VM-like capillary network formation in cervical cancer cells. (**A**, **B**) Matrigel tube formation assay in HeLa (**A**) and SiHa (**B**) cells transfected with control siRNA (Si-NC) or two independent EPHB2 siRNAs (Si-2, Si-3). Representative images and quantification of junctions, number of meshes, and total tube length are shown; values were normalized to Si-NC (set as 1). (**C**, **D**) Matrigel tube formation assay in HeLa (**C**) and SiHa (**D**) cells transfected with empty vector or EPHB2 overexpression plasmid (EPHB2-OE). Representative images and quantification of junctions, number of meshes, and total tube length are shown; values were normalized to vector control (set as 1). Equal numbers of viable cells were seeded in each well for all experimental groups. Quantitative data are presented as mean ± SD from three independent biological experiments. Group comparisons in panels A–B were performed using one-way ANOVA followed by Tukey’s multiple-comparisons test, whereas panels C–D were analyzed using unpaired two-tailed Student’s *t* test. Significance levels are indicated as *P < 0.05, **P < 0.01, ***P < 0.001, ****P < 0.0001. Scale bars, 100 μm

Because vasculogenic mimicry (VM)-like behavior is characterized by the ability of tumor cells to organize into capillary-like networks, we next examined whether EPHB2 modulates this phenotype using Matrigel tube formation assays. In HeLa cells, silencing EPHB2 with two independent siRNAs (Si-2 and Si-3) markedly disrupted network formation compared with the siRNA control (Si-NC), as evidenced by fewer branch points and less connected structures (Fig. 4A). Quantitative analysis confirmed significant reductions in key morphometric parameters, including junction number, mesh number, and total tube length, after EPHB2 knockdown (Fig. 4A). Similar results were observed in SiHa cells, where EPHB2 depletion likewise impaired capillary-like organization and reduced junction number, mesh number, and total tube length relative to Si-NC (Fig. 4B). For these analyses, values were normalized to the corresponding Si-NC controls (set to 1), indicating that EPHB2 supports efficient network formation in both cervical cancer cell lines.

We next examined whether EPHB2 upregulation enhances this network-forming capacity. Overexpression of EPHB2 in HeLa cells resulted in denser and more interconnected tubular structures on Matrigel than in vector controls (Fig. 4C). Consistently, quantification showed significant increases in junction number, mesh number, and total tube length in EPHB2-overexpressing cells (Fig. 4C). This pro-network phenotype was recapitulated in SiHa cells, where EPHB2 overexpression similarly increased the complexity and extent of capillary-like structures and significantly elevated all three parameters relative to vector controls (Fig. 4D). Quantitative values were normalized to vector controls (set to 1), further supporting a gain-of-function effect of EPHB2 on network formation.

Together, these reciprocal loss- and gain-of-function experiments indicate that EPHB2 positively regulates capillary-like network formation in cervical cancer cells, supporting a role for EPHB2 in promoting VM-associated phenotypes in vitro (Fig. 4A–D). Data are presented as mean ± SD from three independent experiments. Statistical analyses were performed using one-way ANOVA with Tukey’s post hoc test for knockdown groups and an unpaired two-tailed Student’s t-test for overexpression experiments, with significance indicated in the corresponding panels.

### Stable EPHB2 knockdown restrains xenograft growth and alters vascular/VM-associated and EMT-related features in vivo

**Fig. 5:**
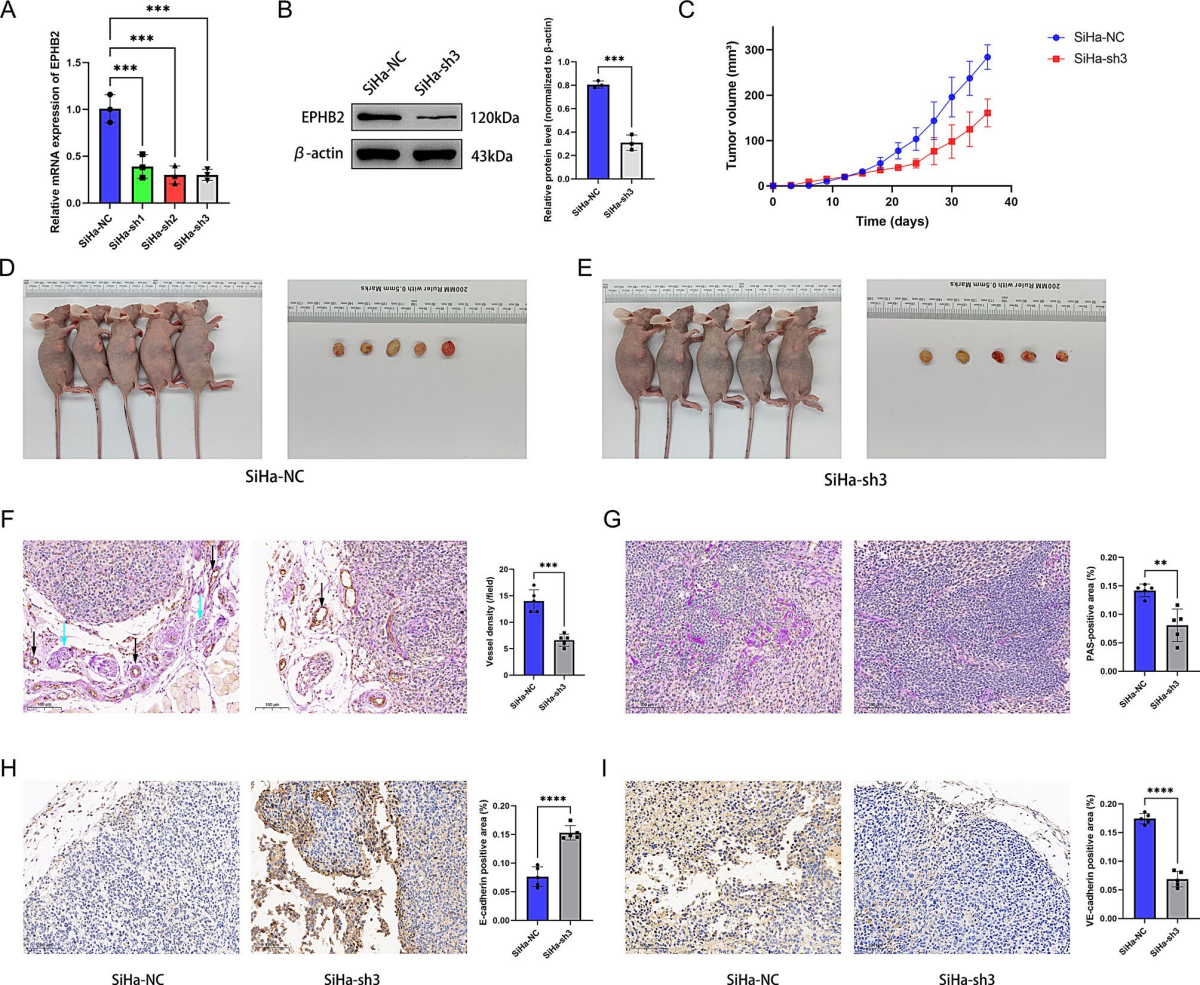
Stable EPHB2 knockdown restrains SiHa xenograft growth and alters vascular/VM-associated and EMT-related features in vivo. (**A**) RT–qPCR analysis of EPHB2 mRNA levels in stably transduced SiHa cells (NC and sh1–sh3). (**B**) Western blot validation of EPHB2 knockdown efficiency in representative stable SiHa-NC and SiHa-sh3 cells, with densitometric analysis normalized to β-actin. (**C**) Tumor growth curves of xenografts established by subcutaneous injection of SiHa-NC or SiHa-sh3 cells. Tumor growth was analyzed by two-way repeated-measures ANOVA with Geisser–Greenhouse correction; a significant time × group interaction was observed (*P* < 0.0001). (**D**–**E**) Representative images of mice and excised tumors from the SiHa-NC and SiHa-sh3 groups at the experimental endpoint. (**F**) Representative CD31/PAS-stained xenograft sections showing vascular structures and quantification of vessel density. Black arrows indicate CD31-positive vessels, whereas blue arrows indicate putative CD31-negative vascular-like structures. (**G**) Representative avascular regions selected from the same CD31/PAS-stained sections shown in (**F**), used to compare PAS-positive histologic patterns while minimizing interference from overt vascular structures; quantification of PAS-positive area is shown on the right. (**H**) E-cadherin IHC and quantification of positive area. (**I**) VE-cadherin IHC and quantification of positive area. Quantitative data are presented as mean ± SD. For in vivo analyses (**C**–**I**), *n* = 5 mice per group. Panel C was analyzed using two-way repeated-measures ANOVA with Geisser–Greenhouse correction, whereas panels F–I were analyzed using unpaired two-tailed Student’s *t* test. *P < 0.05, **P < 0.01, ***P < 0.001, ****P < 0.0001. Scale bars, 100 μm

To determine whether EPHB2 contributes to cervical cancer progression in vivo, we generated stable EPHB2-silenced SiHa cells using three independent shRNAs. RT–qPCR screening showed that EPHB2 mRNA was significantly reduced in each knockdown line compared with the negative control, with sh3 producing robust suppression and therefore selected for xenograft studies (Fig. 5A). Knockdown efficiency was further validated at the protein level by western blotting in representative stable lines (SiHa-NC and SiHa-sh3), and densitometric analysis normalized to β-actin confirmed marked EPHB2 reduction in sh3 cells (Fig. 5B).

We then established subcutaneous xenografts by injecting SiHa-NC or SiHa-sh3 cells into nude mice (*n* = 5 per group) and monitored tumor growth over time. Tumors derived from SiHa-sh3 cells grew substantially more slowly than controls, resulting in a significantly reduced tumor volume trajectory across the observation period (two-way repeated-measures ANOVA with Geisser–Greenhouse correction; time × group interaction: F(12, 96) = 21.27, *P* < 0.0001; group effect: *P* = 0.0031) (Fig. 5C). Consistent with the growth curves, endpoint gross examination showed smaller tumors in the SiHa-sh3 group than in the SiHa-NC group, as illustrated by representative images of mice and excised xenografts (Fig. 5D–E). These results indicate that EPHB2 supports efficient tumor outgrowth in vivo.

To assess how stable EPHB2 silencing affects vascular/VM-associated histologic features within tumors, we examined CD31/PAS-stained sections and matrix-associated patterns. Histologic evaluation showed that SiHa-NC xenografts contained more vascular structures than SiHa-sh3 tumors, including CD31-positive vessels and occasional CD31-negative vascular-like structures in the control group (Fig. 5F). In the representative images, black arrows indicate CD31-positive vessels, whereas blue arrows indicate putative CD31-negative vascular-like structures. These putative structures were identified based on a relatively complete PAS-positive circular outline, absence of evident CD31 staining, and erythrocyte-containing round luminal profiles in section. By contrast, such CD31-negative vascular-like structures were not observed in the representative EPHB2-knockdown sections examined. Quantification further demonstrated significantly reduced vessel density compared with controls (Fig. 5F), suggesting attenuated vascular/VM-associated remodeling following EPHB2 knockdown.

To compare PAS-positive matrix-associated patterns while minimizing interference from overt vascular structures, we selected representative avascular regions from the same CD31/PAS-stained sections. In these regions, PAS-positive area was decreased in EPHB2-silenced tumors (Fig. 5G), consistent with attenuation of PAS-positive histologic patterns associated with vascular/VM-related remodeling.

Given the in vitro evidence that EPHB2 regulates EMT marker expression, we examined EMT/VM-related markers in xenograft tissues. E-cadherin staining was increased in SiHa-sh3 tumors relative to SiHa-NC, with quantification confirming a significantly higher E-cadherin-positive area after EPHB2 depletion (Fig. 5H), indicating reinforcement of epithelial features. Conversely, VE-cadherin, which is frequently linked to VM-related tumor cell plasticity, was markedly reduced in SiHa-sh3 xenografts, and quantitative analysis confirmed a significant decrease in VE-cadherin-positive area compared with controls (Fig. 5I). Taken together, these findings support coordinated reductions in vascular/VM-associated features and a shift away from an EMT/VM-permissive phenotype following stable EPHB2 silencing, although they do not constitute definitive histologic proof of classical VM channels in vivo.

Overall, stable EPHB2 knockdown in SiHa cells suppressed xenograft growth and was accompanied by reduced vessel density, decreased PAS-positive histologic patterns, increased epithelial differentiation as indicated by E-cadherin, and diminished VE-cadherin expression (Fig. 5A–I). Data are presented as mean ± SD; *n* = 5 mice per group for in vivo analyses. Tumor growth curves were analyzed using two-way repeated-measures ANOVA with Geisser–Greenhouse correction, and other comparisons were assessed using an unpaired two-tailed Student’s t-test, with significance indicated in the corresponding panels.

### EPHB2 regulates an ERK–ETV4-associated transcriptional program and downstream functional phenotypes

**Fig. 6:**
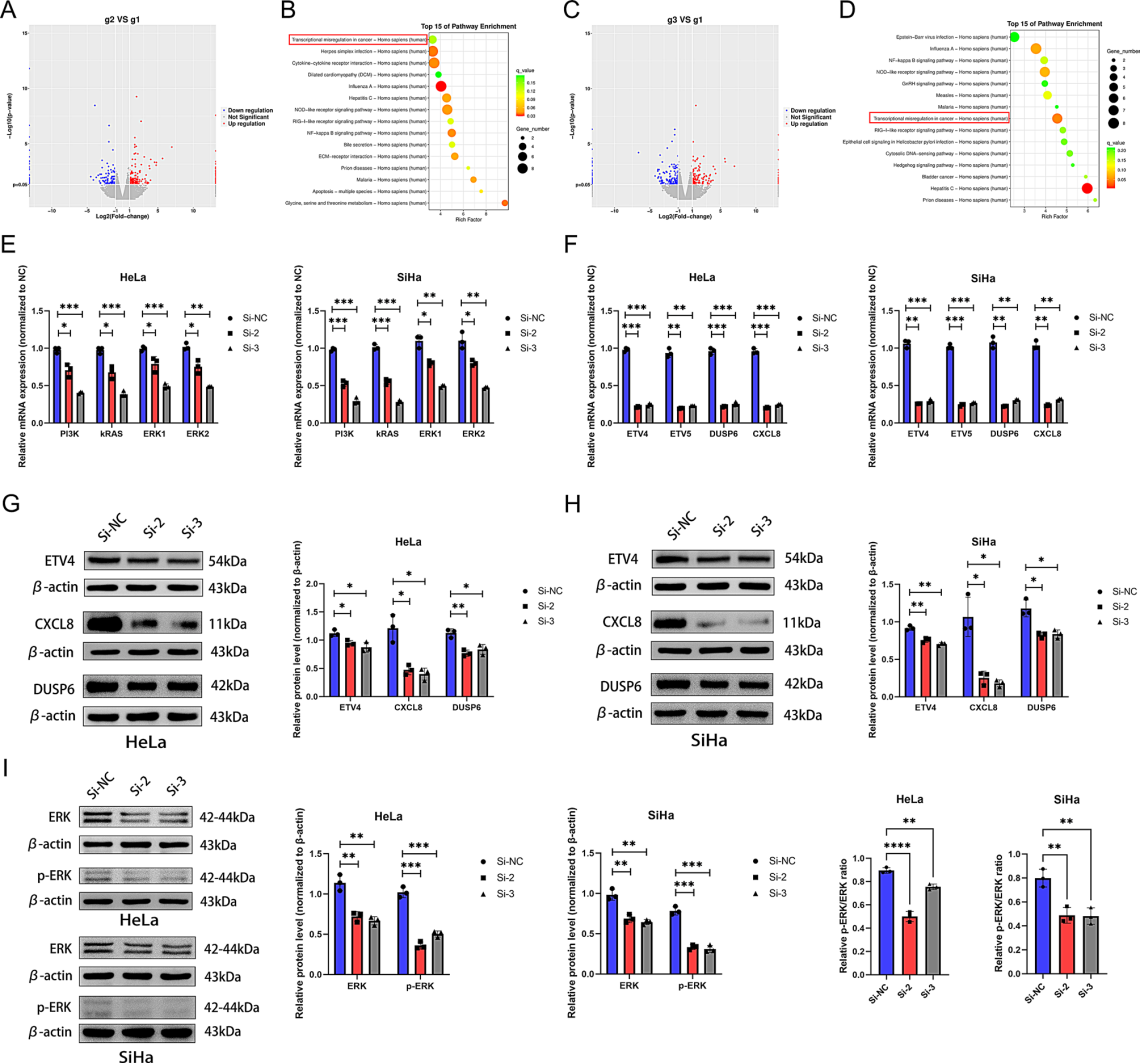
RNA-seq analysis and targeted validation identify ERK-associated signaling and downstream transcriptional alterations following EPHB2 silencing in cervical cancer cells. (**A**–**B**) RNA-seq comparison between control cells (Si-NC; g1) and EPHB2 siRNA-2 (Si-2; g2). Shown are the differential expression results (volcano plot; g2 vs. g1) and the top 15 enriched KEGG pathways based on the final DEG set defined by the predefined adaptive filtering strategy; the pathway “Transcriptional misregulation in cancer” is highlighted. (**C**–**D**) RNA-seq comparison between control cells (Si-NC; g1) and EPHB2 siRNA-3 (Si-3; g3). Shown are the differential expression results (volcano plot; g3 vs. g1) and the top 15 enriched KEGG pathways based on the final DEG set defined by the predefined adaptive filtering strategy; “Transcriptional misregulation in cancer” is highlighted as a reproducible enriched term across both knockdown datasets. (**E**) qRT–PCR validation of selected pathway-associated candidate genes (PI3K, KRAS, ERK1, ERK2) in HeLa and SiHa cells transfected with Si-NC, Si-2, or Si-3. (**F**) qRT–PCR validation of selected downstream genes (ETV4, ETV5, DUSP6, CXCL8) following EPHB2 knockdown in HeLa and SiHa cells. (**G**–**H**) Immunoblot analysis of ETV4, CXCL8, and DUSP6 in HeLa (**G**) and SiHa (**H**) cells after EPHB2 knockdown, with densitometric quantification normalized to β-actin. (**I**) Immunoblot analysis of ERK and p-ERK in HeLa and SiHa cells following EPHB2 knockdown, with densitometric quantification normalized to β-actin; relative ERK phosphorylation is shown as the p-ERK/ERK ratio. Quantitative data are presented as mean ± SD from at least three independent experiments unless otherwise indicated. Differential expression analyses in panels A and C were performed using the edgeR package with Benjamini–Hochberg correction. Genes meeting q-value ≤ 0.05 and |log₂ fold change| ≥ 1 were reported as FDR-significant DEGs. Because the number of FDR-significant DEGs was limited, nominally significant genes with P-value ≤ 0.05 and |log₂ fold change| ≥ 1 were used for pathway-enrichment hypothesis generation. KEGG enrichment analyses in panels B and D were performed using these nominally significant gene sets by over-representation analysis, followed by Benjamini–Hochberg adjustment at the pathway-enrichment level. Panels E, F, G, H, and I were analyzed using one-way ANOVA followed by Tukey’s post hoc test. *P < 0.05, **P < 0.01, ***P < 0.001, ****P < 0.0001

To systematically define EPHB2-regulated transcriptional programs, we performed RNA sequencing after EPHB2 silencing with two independent siRNAs and prioritized signals reproducible across perturbations. In the Si-2 vs. Si-NC comparison (g2 vs. g1), differential expression analysis revealed clear separation of up- and downregulated genes in the volcano plot (Fig. 6A). Using edgeR, 14 genes met the stringent q-value ≤ 0.05 and |log₂ fold change| ≥ 1 criterion. Because this number was below the predefined minimum DEG count for downstream enrichment analysis, the filtering threshold was automatically adjusted to P-value ≤ 0.05 while retaining |log₂ fold change| ≥ 1, resulting in 449 genes for pathway analysis. KEGG pathway enrichment based on this final DEG set highlighted a restricted group of highly ranked pathways after pathway-level multiple-testing correction (Top 15; Fig. 6B). This pattern was independently assessed using a second siRNA comparison (Si-3 vs. Si-NC; g3 vs. g1), in which 7 genes met the stringent q-value ≤ 0.05 and |log₂ fold change| ≥ 1 criterion; after automatic adjustment to P-value ≤ 0.05 with |log₂ fold change| ≥ 1 retained, 516 genes were included in pathway analysis. KEGG enrichment based on this second final DEG set again yielded a highly concordant enrichment profile (Fig. 6C–D). Notably, “Transcriptional misregulation in cancer” emerged as the only tumor-related pathway reproducibly retained among the Top 15 enriched terms in both datasets, providing a stringent basis for downstream mechanistic prioritization.

Guided by this convergent RNA-seq signal, we performed targeted validation of representative pathway-associated and transcriptional components. qRT–PCR analysis showed that EPHB2 silencing reduced the mRNA levels of PI3K, KRAS, ERK1, and ERK2 in both HeLa and SiHa cells (Fig. 6E), indicating coordinated perturbation of pathway-associated signaling nodes at the transcriptional level. We then examined candidate transcriptional outputs identified by RNA-seq. EPHB2 knockdown consistently decreased ETV4 expression, together with ETV5, DUSP6, and CXCL8 (Fig. 6F), supporting a model in which EPHB2 sustains a coordinated cancer-relevant transcriptional program rather than regulating isolated targets.

At the protein level, these observations were further supported by immunoblotting. In both HeLa and SiHa cells, EPHB2 silencing reduced ETV4 abundance, accompanied by decreased CXCL8 and DUSP6 expression (Fig. 6G–H). We next evaluated whether ERK pathway activity was concurrently affected. Western blot analysis showed that EPHB2 depletion reduced both ERK and p-ERK levels, and quantification of the p-ERK/ERK ratio confirmed a significant decrease in relative ERK phosphorylation in both cell lines (Fig. 6I). Together, these data link the transcriptomic signature to concordant attenuation of ERK-associated signaling output and downstream transcriptional effectors.

**Fig. 7:**
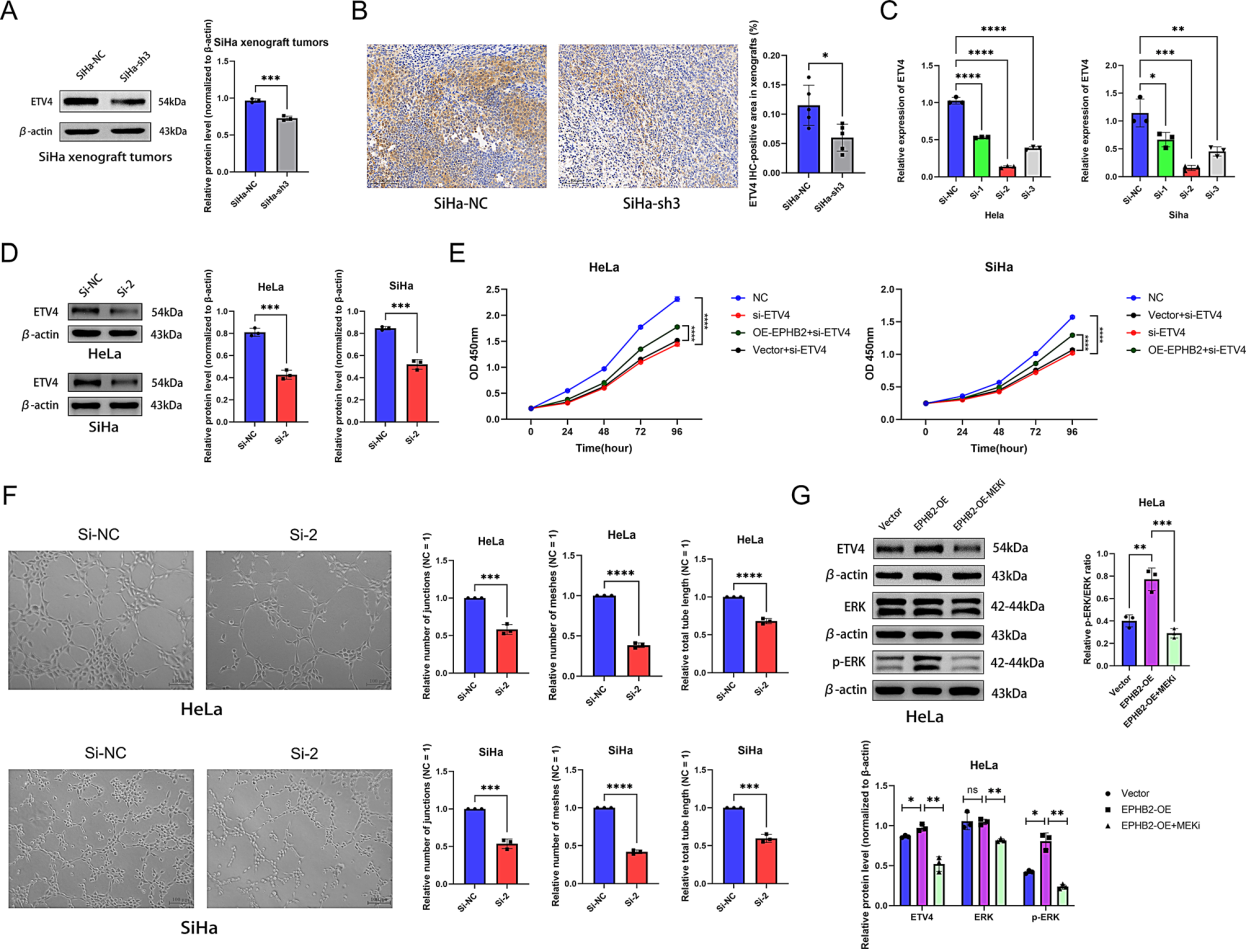
In vitro and in vivo validation supports ETV4 as a downstream effector and links EPHB2-associated ERK signaling to proliferative and VM-like phenotypes. (**A**) Western blot analysis of ETV4 expression in xenograft tumors derived from SiHa-NC and SiHa-sh3 cells, with densitometric quantification normalized to β-actin. (**B**) Representative images of ETV4 immunohistochemistry in xenograft tumors and quantification of ETV4-positive area fraction in the SiHa-NC and SiHa-sh3 groups. (**C**) qRT–PCR screening of siRNAs targeting ETV4 in HeLa and SiHa cells. (**D**) Western blot confirmation of ETV4 knockdown in HeLa and SiHa cells, with densitometric quantification normalized to β-actin. (**E**) CCK-8 assays assessing cell proliferation after ETV4 silencing and in EPHB2-overexpressing cells under ETV4 knockdown conditions (groups as indicated). (**F**) Matrigel tube formation assays after ETV4 knockdown in HeLa and SiHa cells, with quantification of junction number, mesh formation, and total tube length (normalized to control, set as 1). (**G**) Immunoblot analysis of ETV4, ERK, and p-ERK in vector control, EPHB2-overexpression (EPHB2-OE), and EPHB2-OE + MEK inhibitor groups in HeLa cells, with densitometric quantification normalized to β-actin; relative ERK phosphorylation is shown as the p-ERK/ERK ratio. Quantitative data are presented as mean ± SD from at least three independent experiments unless otherwise indicated. Panels C and G were analyzed using one-way ANOVA followed by Tukey’s post hoc test. Panel E (CCK-8 time-course assay) was analyzed using two-way ANOVA. Panels A, B, D, and F were analyzed using unpaired two-tailed Student’s t test. *P < 0.05, **P < 0.01, ***P < 0.001, ****P < 0.0001. Scale bars, 100 μm

To determine whether the key transcriptional regulator identified above is functionally relevant and retained in vivo, we performed additional validation experiments. In xenograft tumors derived from stable EPHB2-knockdown cells (SiHa-sh3) and control cells (SiHa-NC), ETV4 protein abundance was markedly reduced in the EPHB2-depleted group by western blotting (Fig. 7A). This finding was further supported by immunohistochemistry, which showed significantly lower ETV4-positive area in the SiHa-sh3 group than in controls (Fig. 7B), indicating that the EPHB2-associated transcriptional regulator identified in vitro was consistently suppressed in vivo.

Given the reproducible downregulation of ETV4 across RNA-seq, in vitro validation, and xenograft tissues, we next examined whether ETV4 is functionally required for EPHB2-associated phenotypes. Among the siRNAs tested, si-2 achieved robust suppression of ETV4 expression at both the mRNA and protein levels (Fig. 7C–D) and was therefore selected for functional assays. ETV4 silencing impaired cell proliferation in both HeLa and SiHa cells and, notably, attenuated the proliferative advantage conferred by EPHB2 overexpression (Fig. 7E), supporting ETV4 as a functional downstream mediator rather than a parallel marker. In addition, ETV4 depletion markedly compromised Matrigel tube formation, as reflected by reductions in junction number, mesh number, and total tube length in both cell lines (Fig. 7F), indicating that ETV4 contributes to VM-like network formation.

To further assess pathway dependency, we performed pharmacologic inhibition experiments targeting the ERK pathway. In EPHB2-overexpressing HeLa cells, EPHB2 overexpression increased ETV4 expression together with ERK activation, as indicated by elevated p-ERK levels. Importantly, treatment with a MEK inhibitor reduced p-ERK levels and attenuated ETV4 upregulation, with quantification of the p-ERK/ERK ratio confirming suppression of ERK activation (Fig. 7G). These findings provide additional functional evidence linking EPHB2-associated ERK signaling to downstream ETV4 regulation.

Collectively, these results support a functionally connected EPHB2–ERK–ETV4 axis in which EPHB2 sustains ERK-associated signaling output and downstream ETV4-related transcriptional programs, thereby promoting proliferative capacity and VM-like phenotypes in cervical cancer cells. At the same time, because reciprocal rescue experiments were not performed, the current data support the functional requirement of ETV4 but do not fully establish that ETV4 is sufficient to mediate all EPHB2-driven effects.

### Integrated working model of EPHB2-driven EMT/VM programs in cervical cancer

**Fig. 8:**
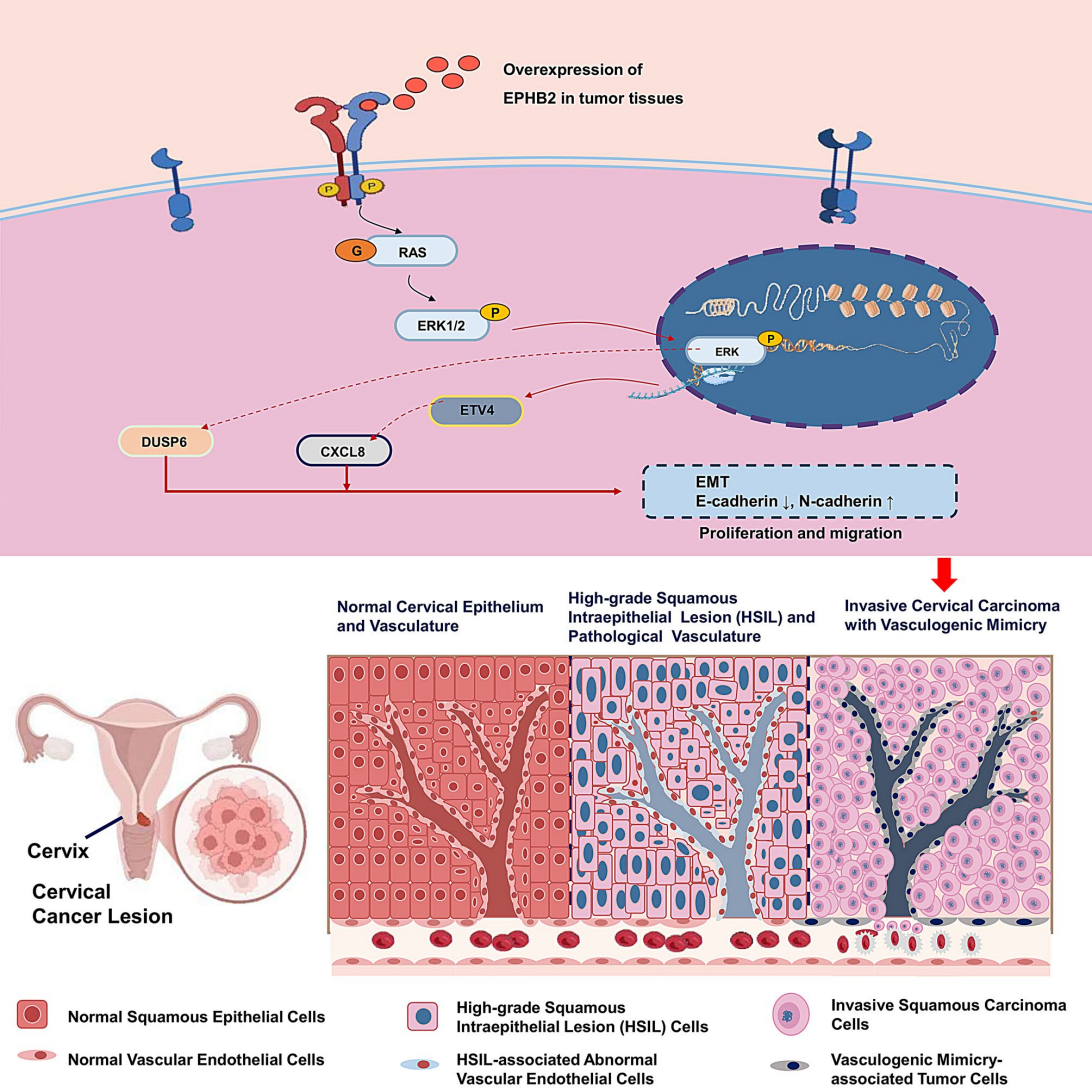
Working model of EPHB2–ERK–ETV4 signaling in EMT-associated plasticity and vasculogenic mimicry in cervical cancer. Schematic overview of the proposed mechanism by which EPHB2 upregulation in cervical cancer activates ERK-associated signaling and induces ETV4-associated transcriptional outputs, including DUSP6 and CXCL8. These changes promote an EMT-like shift, characterized by decreased E-cadherin and increased N-cadherin, and enhance proliferative and migratory capacity, thereby supporting vasculogenic mimicry-associated tumor progression. The lower panel illustrates a conceptual progression from normal cervical epithelium and vasculature to HSIL-associated pathological vasculature and invasive cervical carcinoma with vasculogenic mimicry. This working model integrates the findings presented in Figs. 1, 2, 3, 4, 5, 6 and 7; solid arrows indicate relationships supported by data from this study, whereas dashed arrows denote putative links

Collectively, the clinical, transcriptomic, and functional data support a unified model in which EPHB2 acts as an upstream regulator of malignant progression in cervical cancer (Fig. 8). EPHB2 is upregulated in tumor tissues and is associated with unfavorable survival and enhanced EMT-related transcriptional activity (Fig. 1). Functional perturbation experiments further show that EPHB2 promotes proliferative and clonogenic capacity, enhances migratory behavior, and confers resistance to apoptosis in cervical cancer cells (Figs. 2 and 3). In parallel, EPHB2 positively regulates capillary-like network formation on Matrigel and modulates VM/vascularization- and EMT-related features in vivo, including reduced PAS-positive patterns and VE-cadherin, together with restoration of E-cadherin, upon stable knockdown (Figs. 4 and 5).

Mechanistically, two independent RNA-seq comparisons converged on “Transcriptional misregulation in cancer” as a reproducible pathway-level signal downstream of EPHB2 depletion (Fig. 6A–I). Targeted validation further linked EPHB2 to ERK pathway output and an ETV4-centered transcriptional program: EPHB2 knockdown reduced p-ERK and decreased ERK-associated outputs (DUSP6 and CXCL8) together with ETV4 in vitro, while ETV4 was also diminished in EPHB2-silenced xenografts (Fig. 7A–B). Importantly, ETV4 silencing suppressed cell growth, impaired VM-like tube formation, and attenuated the growth advantage associated with EPHB2 overexpression (Fig. 7C–F). In addition, MEK inhibitor treatment attenuated ERK phosphorylation and reduced ETV4 upregulation in EPHB2-overexpressing cells, providing further functional support for the EPHB2–ERK–ETV4 signaling relationship (Fig. 7G). Together, these findings, summarized in Fig. 8, delineate an EPHB2–ERK–ETV4 axis that supports EMT-associated programs and VM-like phenotypes during cervical cancer progression.

## Discussion

In this study, we integrated clinical specimens, bulk transcriptomic datasets, single-cell RNA-seq, and gain- and loss-of-function experiments to define the role of EPHB2 in cervical cancer progression. Several lines of evidence converge on a consistent conclusion: EPHB2 is upregulated in cervical cancer, associated with adverse outcome, and functionally contributes to a malignant cellular state characterized by enhanced proliferation, migration, EMT-like features, and vasculogenic mimicry (VM)-like network formation. Mechanistically, two independent RNA-seq comparisons following EPHB2 knockdown identified a reproducible pathway-level signal, “Transcriptional misregulation in cancer”, and subsequent targeted validation supported an EPHB2–ERK–ETV4 signaling module with downstream transcriptional outputs including DUSP6 and CXCL8. Together, these findings position EPHB2 as an upstream regulator that links oncogenic signaling to transcriptional reprogramming and phenotypic plasticity in cervical cancer. More specifically, our data support a model in which EPHB2 sustains ERK-associated signaling and an ETV4-centered transcriptional program, thereby promoting EMT/VM-related plasticity and malignant progression. This mechanistic framework suggests that EPHB2-high cervical cancers may represent a biologically distinct subset with potential vulnerability to pathway-informed therapeutic intervention.

A major strength of this study is the concordance across platforms and biological levels. At the clinical level, EPHB2 protein was elevated in tumor tissues compared with non-tumor cervix in our IHC cohort, consistent with increased mRNA expression in the integrated TCGA–GTEx dataset. Importantly, high EPHB2 expression stratified TCGA-CESC patients with worse overall survival, supporting its relevance beyond a descriptive biomarker. Although cervical cancer is clinically heterogeneous, concordant EPHB2 upregulation across tissue specimens, bulk RNA-seq, and outcome analysis suggests that EPHB2 activation reflects a broader progression-linked biological state.

At the bulk transcriptomic level, this state was not limited to EMT-related features but also extended to immune-regulatory programs. EPHB2 expression was positively associated with several immunoregulatory molecules, with SIGLEC15 showing the most prominent correlation, together with broader associations involving myeloid- and inflammatory-response-related features. These observations suggest that EPHB2-high cervical cancers may exist in a distinct immune-related transcriptional context characterized by coordinated variation in immunoregulatory and myeloid-associated programs. However, these findings remain correlative and should be interpreted cautiously. Because the present study did not include immune co-culture assays, immune-marker profiling in xenografts, or immunocompetent in vivo models, we cannot conclude that EPHB2 directly shapes the tumor immune microenvironment in cervical cancer. Rather, these immune-related observations should be viewed as hypothesis-generating and as a rationale for future studies addressing whether EPHB2 contributes to TIME remodeling during cervical cancer progression.

The single-cell analyses refined this interpretation by mapping EPHB2-associated signals to the tumor epithelial compartment and linking EPHB2-high cells to EMT/VM-related epithelial states. This is conceptually important because bulk transcriptomic associations may reflect differences in cellular composition or stromal infiltration. By resolving cell states at single-cell resolution, our data support the view that EPHB2 is embedded in a tumor-cell-intrinsic plasticity program rather than serving solely as a surrogate for microenvironmental variation. Indeed, EPHB2-positive epithelial cells substantially overlapped with EMT/VM co-high cells on the UMAP, and higher EPHB2 expression was associated with an increased probability of belonging to the EMT/VM co-high state, as well as higher EMT and VM-ECM scores. These findings support a link between EPHB2 and a plastic, transition-prone epithelial phenotype, consistent with the in vitro functional data.

Functionally, EPHB2 exerted reciprocal effects on malignant behaviors. EPHB2 silencing impaired proliferation and clonogenicity, reduced migration in both wound-healing and Transwell assays, increased Annexin V/PI-defined apoptotic fractions, and shifted EMT markers toward a more epithelial pattern, with increased E-cadherin and decreased N-cadherin. Conversely, EPHB2 overexpression enhanced growth, clonogenic output, and migratory behavior while reducing TUNEL positivity. These reciprocal phenotypes across independent perturbations reduce the likelihood of off-target effects and support a functional role for EPHB2 in maintaining malignant fitness. This is particularly relevant in cervical cancer, where local invasion and dissemination are major contributors to recurrence and treatment resistance.

A distinctive aspect of this study is its focus on VM-like phenotypes. VM is increasingly recognized as an alternative perfusion strategy that can coexist with or complement endothelial angiogenesis, particularly in aggressive tumors with high plasticity. In vitro, EPHB2 modulated Matrigel network formation in two cervical cancer cell lines, affecting junction number, mesh number, and total tube length. In vivo, stable EPHB2 knockdown reduced xenograft growth and altered vascular/VM-associated readouts, including reduced CD31-defined microvessel density and decreased PAS-positive patterns, accompanied by reduced VE-cadherin and increased E-cadherin. Together, these data suggest that EPHB2 not only promotes classical malignant behaviors but also reinforces plasticity programs compatible with VM-like organization and endothelial-mimicking features.

The mechanistic component of this study was guided by an explicit prioritization strategy designed to minimize false discovery. Rather than selecting pathways from a single transcriptomic comparison, we performed RNA-seq after EPHB2 knockdown using two independent siRNAs (Si-2 and Si-3). Among the Top 15 enriched pathways in each comparison, “Transcriptional misregulation in cancer” emerged as the only tumor-relevant term consistently retained across both datasets. This convergence suggests that the pathway-level signal is robust to siRNA choice and therefore less likely to reflect sequence-specific off-target effects. Subsequent validation further supported this interpretation: qRT–PCR and immunoblotting showed that EPHB2 depletion reduced ERK pathway output, including reduced p-ERK, and decreased the expression of downstream outputs including DUSP6 and CXCL8, together with reduced ETV4. The in vivo relevance of this axis was supported by reduced ETV4 protein abundance and IHC positivity in EPHB2-silenced xenografts.

ETV4 is an attractive mechanistic node because it provides a plausible bridge between upstream kinase signaling and transcriptional reprogramming that drives plasticity-related phenotypes. In this study, ETV4 was linked to the EPHB2-dependent transcriptomic signature and supported by multiple layers of validation. Specifically, EPHB2 knockdown reduced ETV4 protein expression, whereas MEK inhibition in EPHB2-overexpressing cells attenuated both p-ERK and ETV4 upregulation, supporting a functional link between EPHB2-associated ERK signaling and ETV4 regulation. In addition, ETV4 knockdown suppressed proliferation, impaired VM-like tube formation, and attenuated the growth advantage associated with EPHB2 overexpression. Together, these findings support ETV4 as a biologically relevant downstream effector rather than a passive marker. However, because reciprocal rescue experiments were not performed, the current data support the functional requirement of ETV4 in EPHB2-associated phenotypes but do not establish that ETV4 is sufficient to mediate all effects of EPHB2.

The accompanying changes in DUSP6 and CXCL8 are also mechanistically coherent with an ERK-linked transcriptional output state. DUSP6 is commonly regarded as an ERK-inducible feedback phosphatase; therefore, its reduction upon EPHB2 knockdown is compatible with diminished ERK activity rather than implying that DUSP6 drives ERK activation. CXCL8 is frequently associated with inflammatory signaling, tumor–stroma communication, and pro-migratory behavior; its regulation downstream of EPHB2 suggests that EPHB2 may influence both intrinsic tumor programs and extrinsic microenvironmental crosstalk, a possibility that warrants further investigation.

Despite the overall coherence of the dataset, several limitations should be acknowledged. The institutional IHC cohort was small and restricted to early-stage squamous histology, which limits the generalizability of the biomarker-related observations and warrants validation in larger and more diverse clinical cohorts. In addition, although TCGA–GTEx integration improved normal tissue representation, cross-cohort differences in sample processing may still introduce batch-related effects that should be addressed in future harmonized analyses.

Another important limitation concerns the interpretation of VM-related findings in vivo. Although our Matrigel assays provide tumor-cell-intrinsic evidence for VM-like network formation, and CD31/PAS staining in xenografts revealed putative CD31-negative vascular-like structures together with coordinated changes in vascular/VM-associated readouts, these observations should be interpreted cautiously rather than as definitive histologic proof of classical VM channels. More stringent structural validation and additional in vivo models will be needed to strengthen this aspect of the study. Likewise, although the current data support a functional relationship among EPHB2, ERK signaling, and ETV4, the absence of reciprocal rescue experiments limits our ability to determine whether ETV4 is sufficient to fully mediate EPHB2-driven phenotypes; this mechanistic hierarchy will require further clarification in future work.

From a translational perspective, our findings suggest that EPHB2 may serve not only as a biomarker but also as a candidate therapeutic entry point in cervical cancer. As a member of the Eph receptor family, EPHB2 is a cell-surface receptor with potential pharmacologic accessibility. However, Eph signaling is highly context-dependent and may be shaped by ligand engagement, receptor crosstalk, and bidirectional signaling, indicating that any therapeutic strategy would require careful consideration of tumor context and pathway directionality. In addition, the EPHB2–ERK–ETV4 axis identified in this study raises the possibility that EPHB2-high tumors may be particularly dependent on ERK-linked transcriptional programs, providing a rationale for pathway-based intervention. The partial functional dependency on ETV4 further suggests that downstream transcriptional mediators, or their upstream signaling regulators, may represent therapeutically relevant leverage points.

Several EPHB2-directed therapeutic concepts have already been explored in preclinical settings. Early studies showed that monoclonal antibodies against the extracellular domain of EphB2 can recognize EphB2-positive tumor cells, supporting the feasibility of antibody-based targeting strategies [35]. More recently, EphB2-directed antibodies have been developed into antibody–drug conjugates and shown antitumor activity in EphB2-overexpressing xenograft models, highlighting the potential utility of EphB2 as a cell-surface therapeutic target [36]. In parallel, pharmacologic inhibition of Eph-family kinase activity has also shown promise. For example, the small-molecule inhibitor NVP-BHG712 suppresses Eph receptor autophosphorylation, with reported activity that includes EphB2 in cell-based assays, suggesting that Eph kinase signaling is pharmacologically tractable [37]. Taken together, these observations support the biological plausibility of EPHB2-directed or pathway-informed therapeutic strategies in EPHB2-high cervical cancer. Nevertheless, the present study was designed to define the mechanistic role of the EPHB2–ERK–ETV4 axis rather than to directly test pharmacologic inhibition in cervical cancer models. Therefore, the translational implications of our findings should be interpreted cautiously. Future studies should evaluate EPHB2-targeted approaches in cervical cancer-specific models and determine whether p-ERK/ERK and ETV4 can serve as mechanism-informed pharmacodynamic readouts.

In summary, this study identifies EPHB2 as a functional contributor to cervical cancer progression, linking oncogenic signaling to transcriptional regulation and phenotypic plasticity. By integrating clinical validation, bulk and single-cell transcriptomic analyses, functional perturbation experiments, and in vivo confirmation, we provide evidence that EPHB2 sustains an ERK-associated transcriptional program centered on ETV4 and linked to EMT- and VM-associated phenotypes. This mechanistic framework provides a rationale for exploring EPHB2-related pathway vulnerabilities in aggressive cervical cancer.

## Conclusion

This study demonstrates that EPHB2 is consistently upregulated in cervical cancer and is associated with worse overall survival. Integrating institutional IHC data with TCGA–GTEx and single-cell transcriptomic analyses, we show that elevated EPHB2 is associated with EMT/VM-related epithelial states and EMT-associated programs. Functional experiments further establish that EPHB2 promotes malignant phenotypes, including proliferation, clonogenic growth, migration, survival, and capillary-like network formation in vitro. In vivo, stable EPHB2 knockdown suppresses SiHa xenograft growth and is accompanied by reduced vascular/VM-associated features and a shift toward a more epithelial phenotype, including increased E-cadherin and decreased VE-cadherin.

Mechanistically, two independent RNA-seq comparisons converged on “Transcriptional misregulation in cancer” as a robust pathway-level signal downstream of EPHB2 depletion. Subsequent validation supports an EPHB2–ERK–ETV4 signaling module in which EPHB2 sustains ERK pathway output and ETV4-centered transcriptional programs, with downstream effectors including DUSP6 and CXCL8. Importantly, ETV4 knockdown attenuates cell growth and VM-like network formation and diminishes the pro-growth effect of EPHB2 overexpression, supporting ETV4 as a functional mediator. Together, these findings identify EPHB2 as a functional regulator of EMT/VM-associated plasticity in cervical cancer and support the EPHB2–ERK–ETV4 axis as a candidate therapeutic entry point for further investigation in aggressive cervical cancer.

## Electronic Supplementary Material

Below is the link to the electronic supplementary material.

Supplementary Material 1

Supplementary Material 2

## Data Availability

The datasets analyzed in this study are publicly available. Bulk RNA-seq and clinical data were obtained from The Cancer Genome Atlas (TCGA) and the Genotype-Tissue Expression (GTEx) project. Publicly available single-cell RNA-seq datasets were obtained from previously published studies. The RNA-seq data generated in this study have been deposited in the Gene Expression Omnibus (GEO) under accession number GSE326407. Additional data generated or analyzed during this study are available from the corresponding author upon reasonable request.
