# Supplementary material for: EPHB2 drives EMT-linked vasculogenic mimicry and cervical cancer progression via an ERK–ETV4 transcriptional program
Source: J Transl Med. 2026 May 16;24:774. doi: 10.1186/s12967-026-08300-0 (PMC13274032; doi:10.1186/s12967-026-08300-0)
Supplement: Supplementary file 1 — Supplementary Material 1 [file 12967_2026_8300_MOESM1_ESM.pdf]

FIG2_B_Knockdown of EPHB2_HeLa

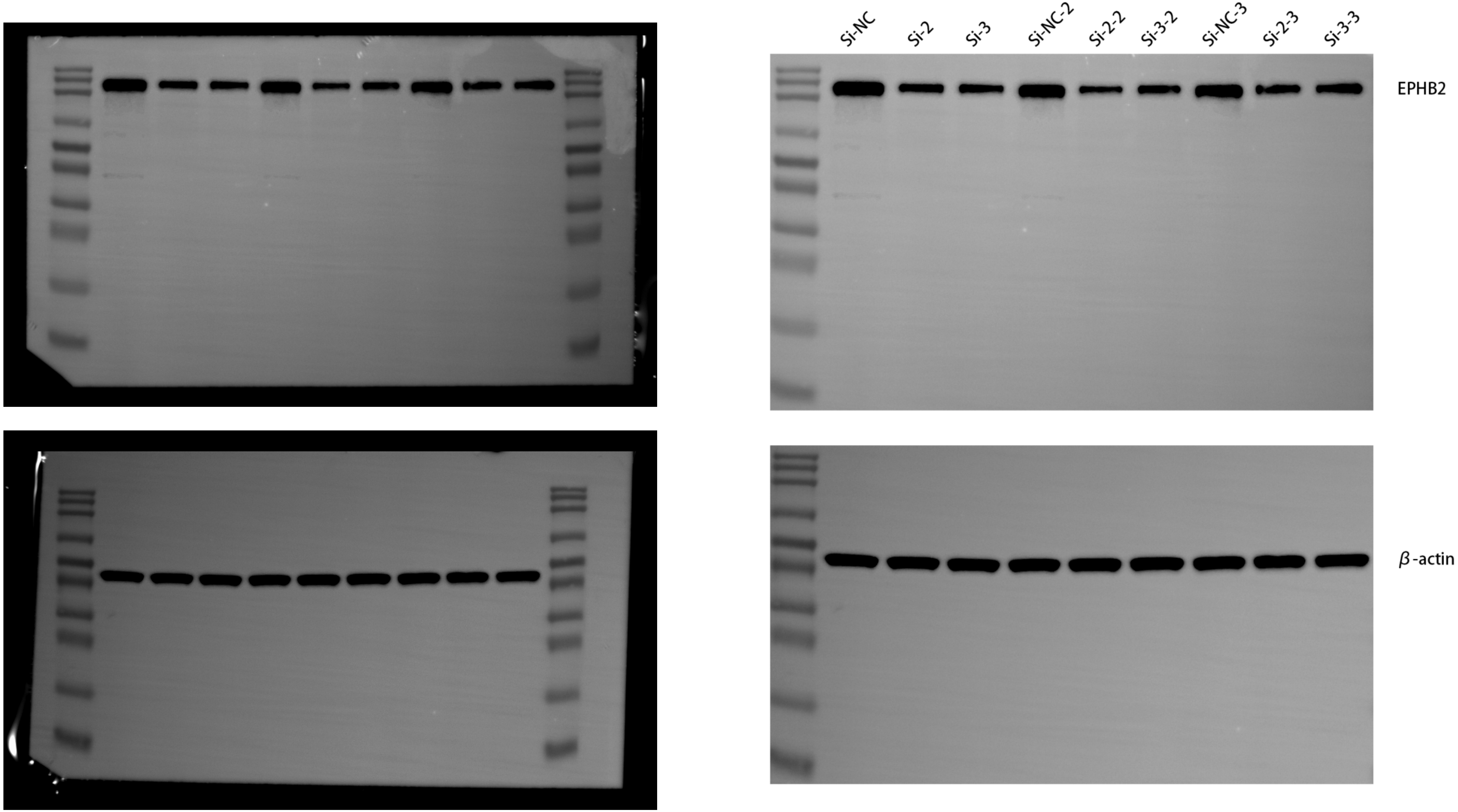

FIG2_B_Knockdown of EPHB2_SiHa

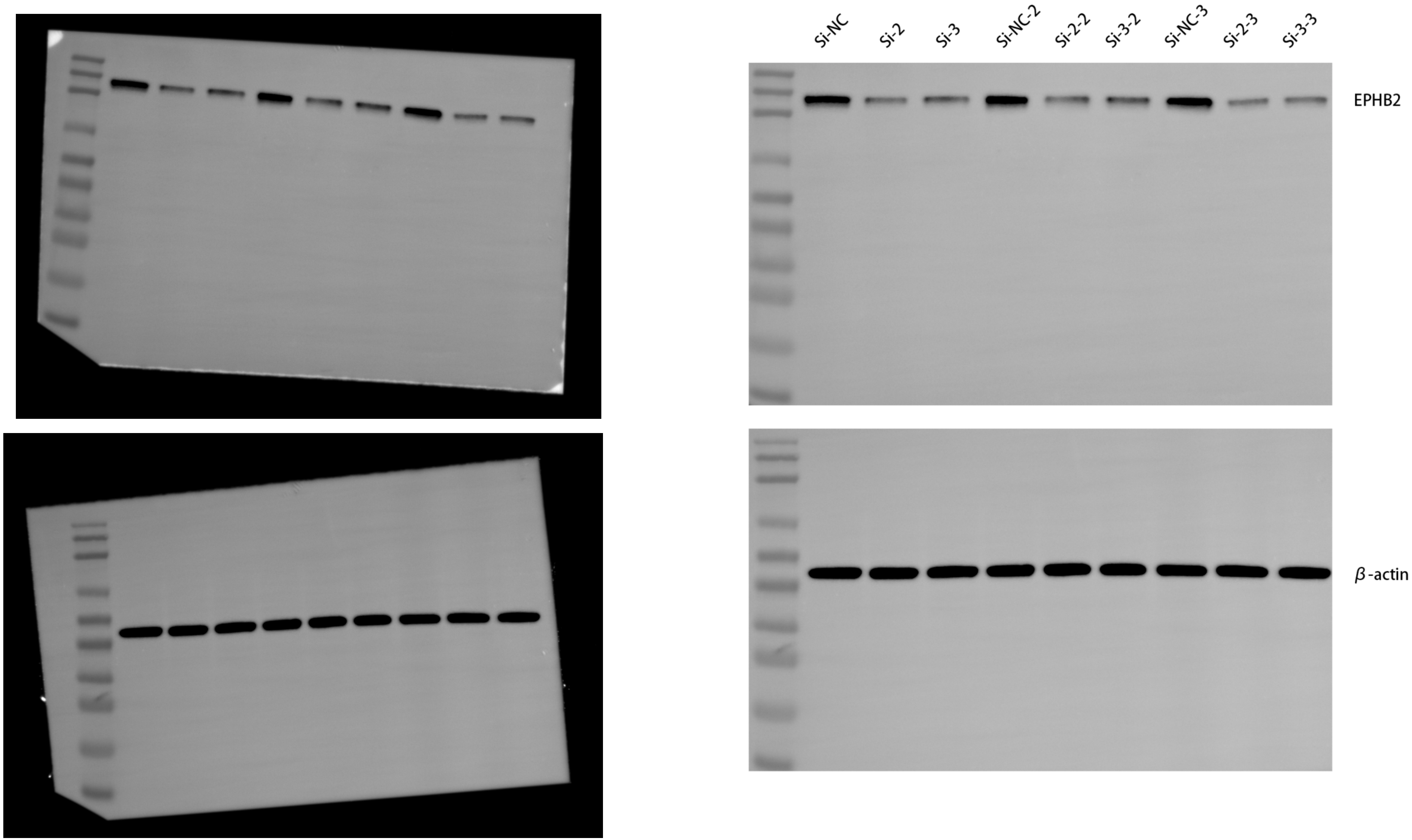

FIG2_H_E-CAD_HeLa

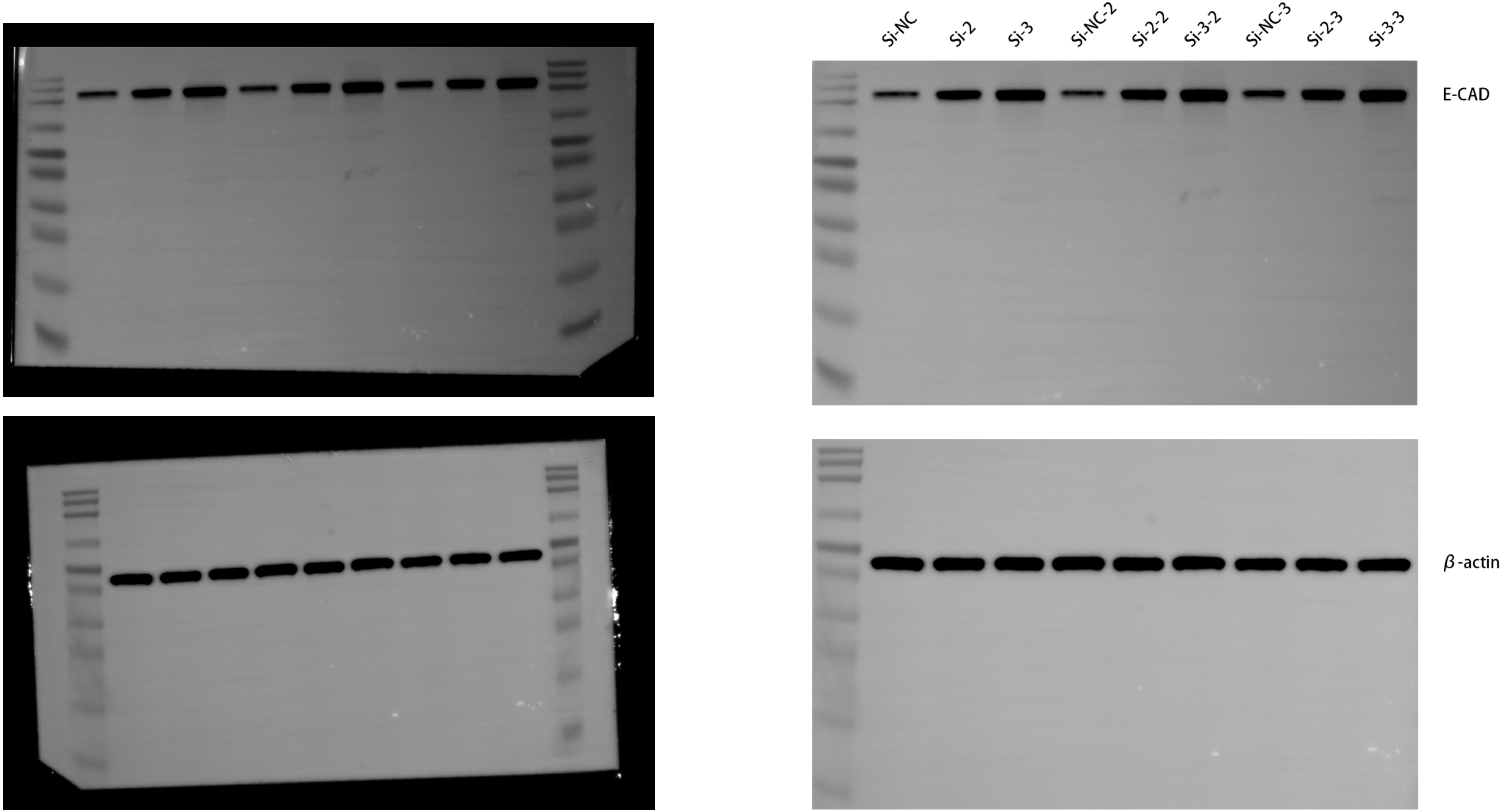

FIG2_H_N-CAD_HeLa

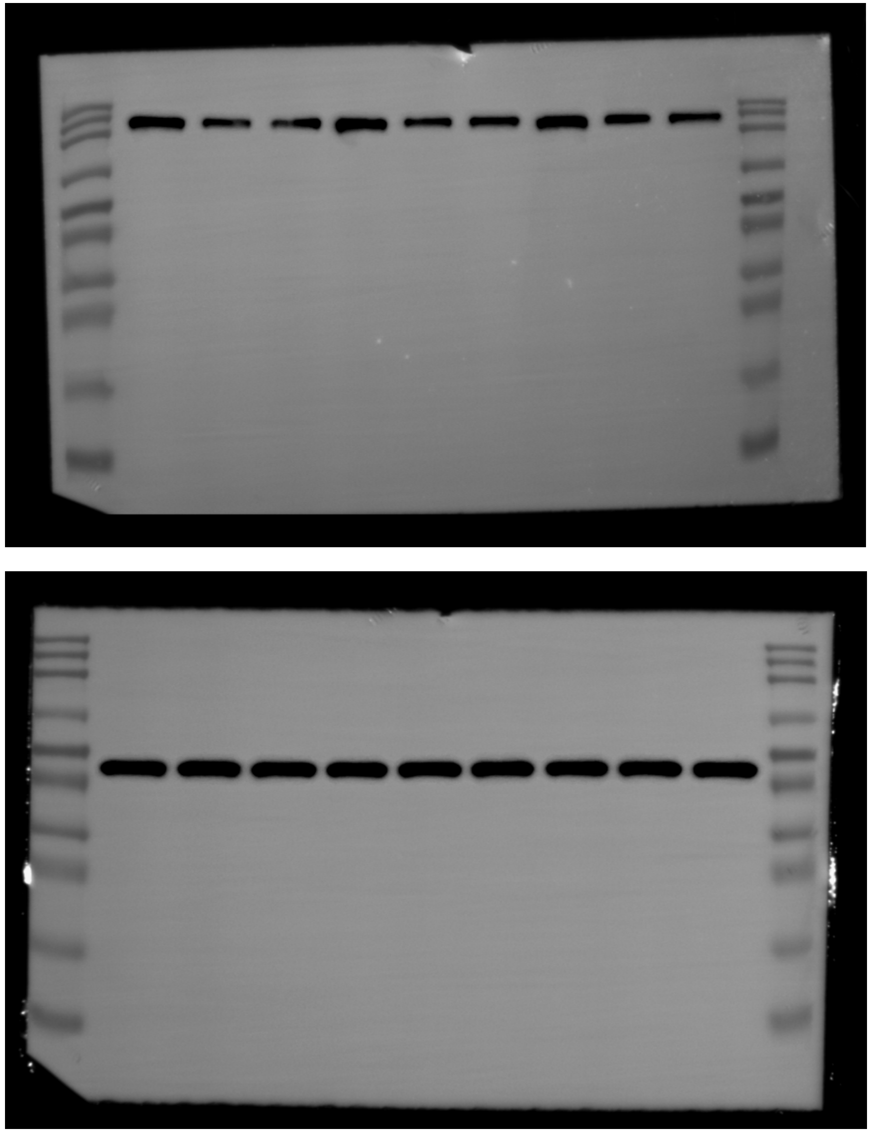

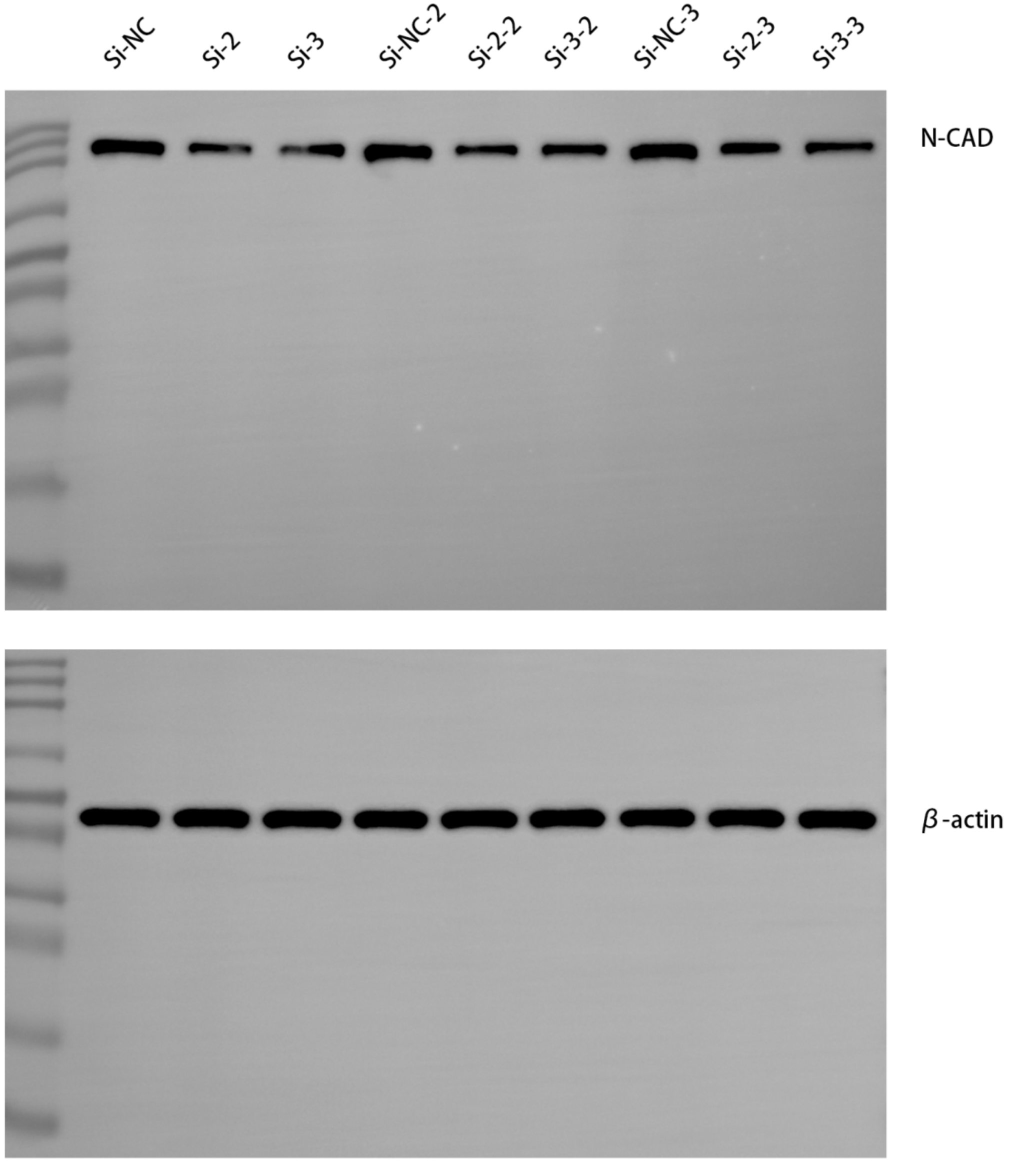

FIG2_H_E-CAD_SiHa

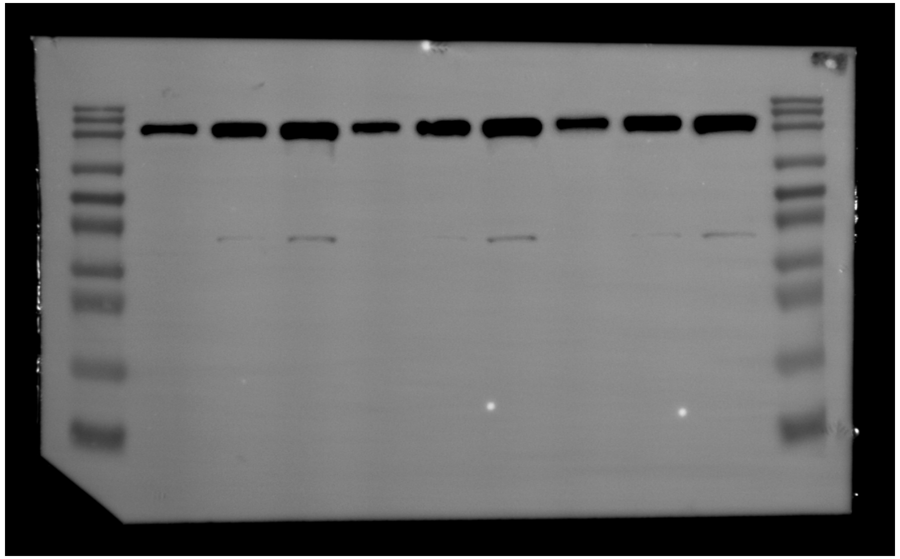

Si-NC Si-2 Si-3 Si-NC-2 Si-2-2 Si-3-2 Si-NC-3 Si-2-3 Si-3-3

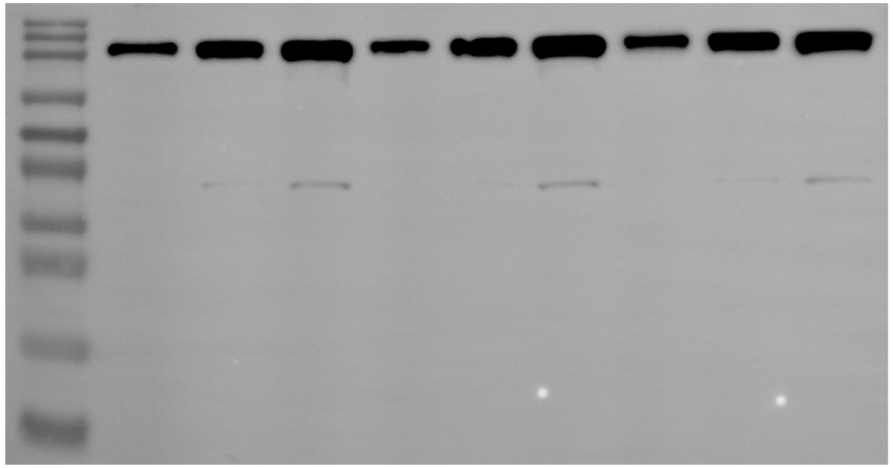

E-CAD

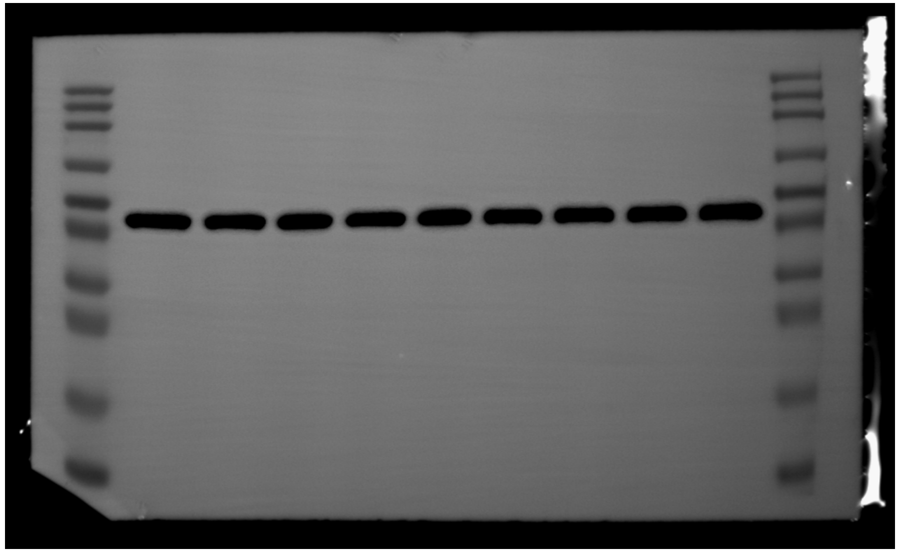

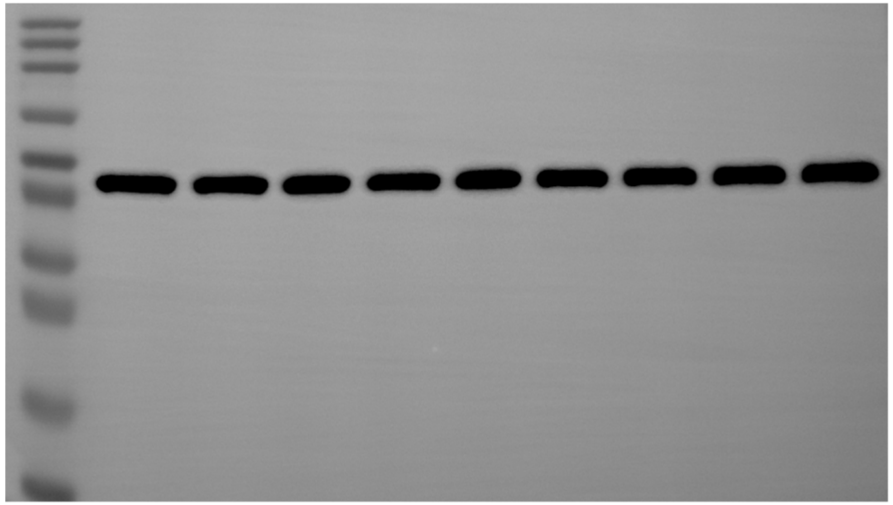

β-actin

FIG2_H_N-CAD_SiHa

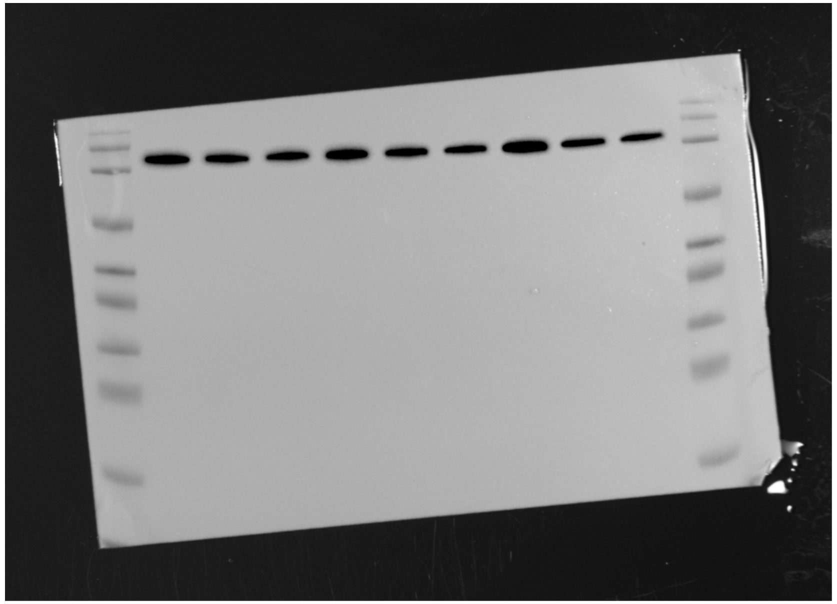

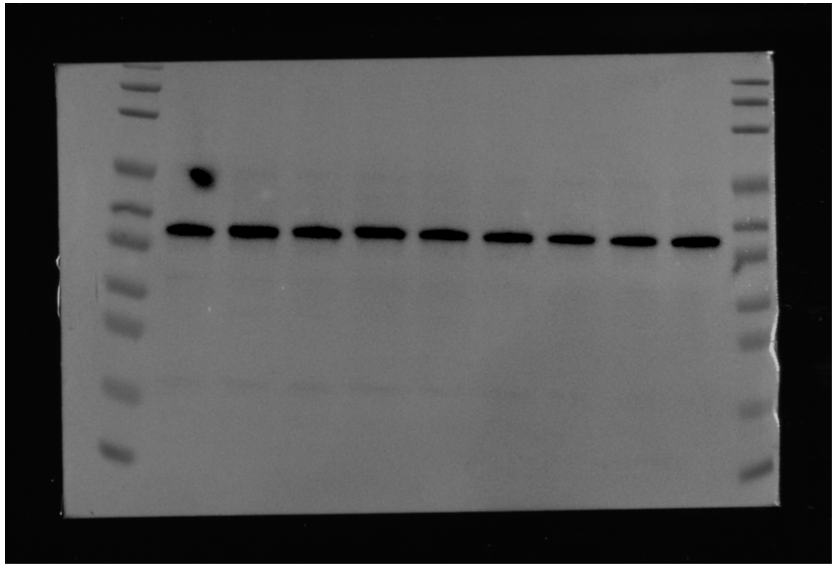

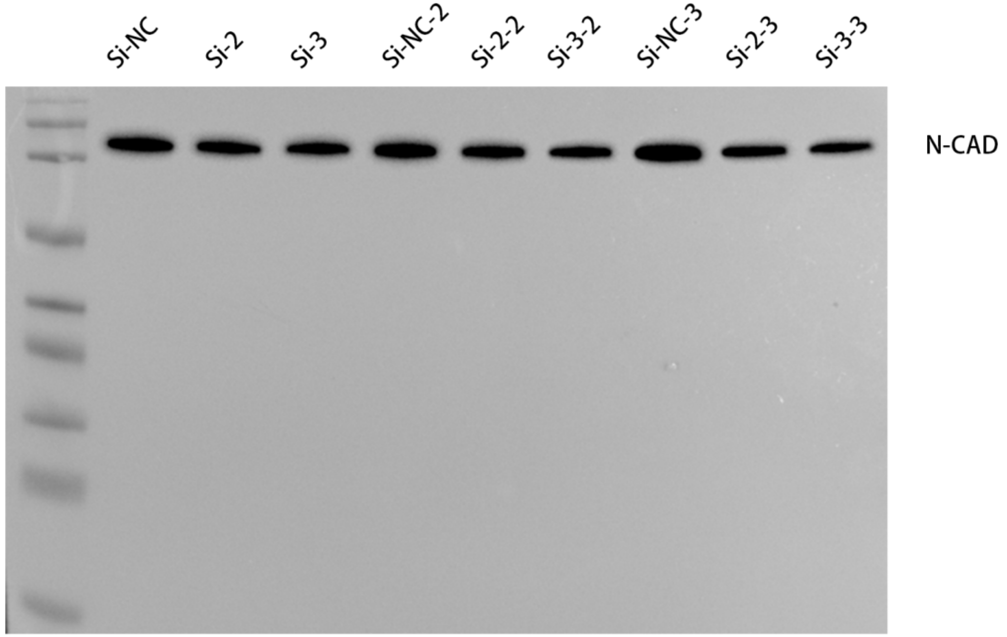

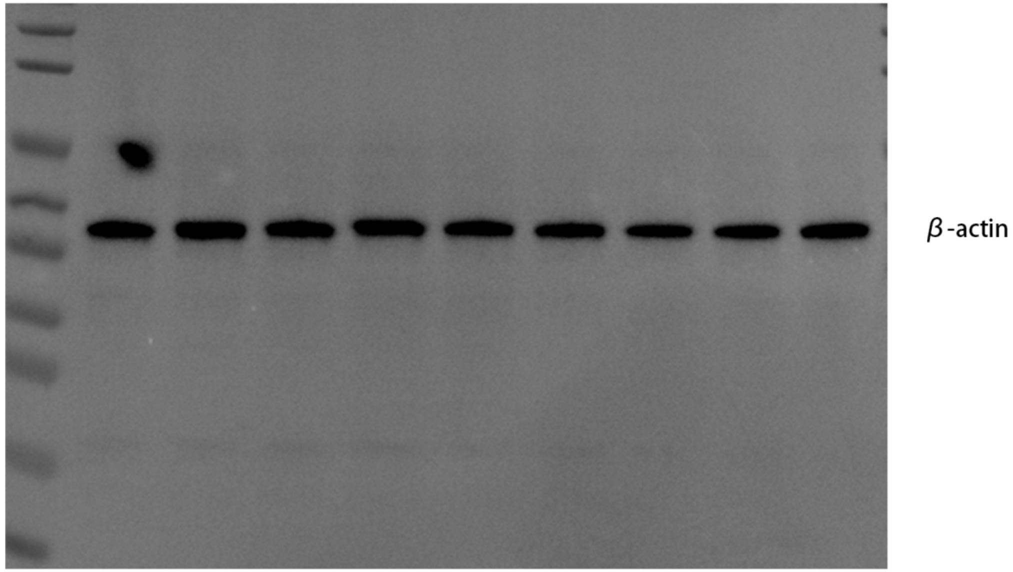

FIG3_A_Overexpression of EPHB2_HeLa

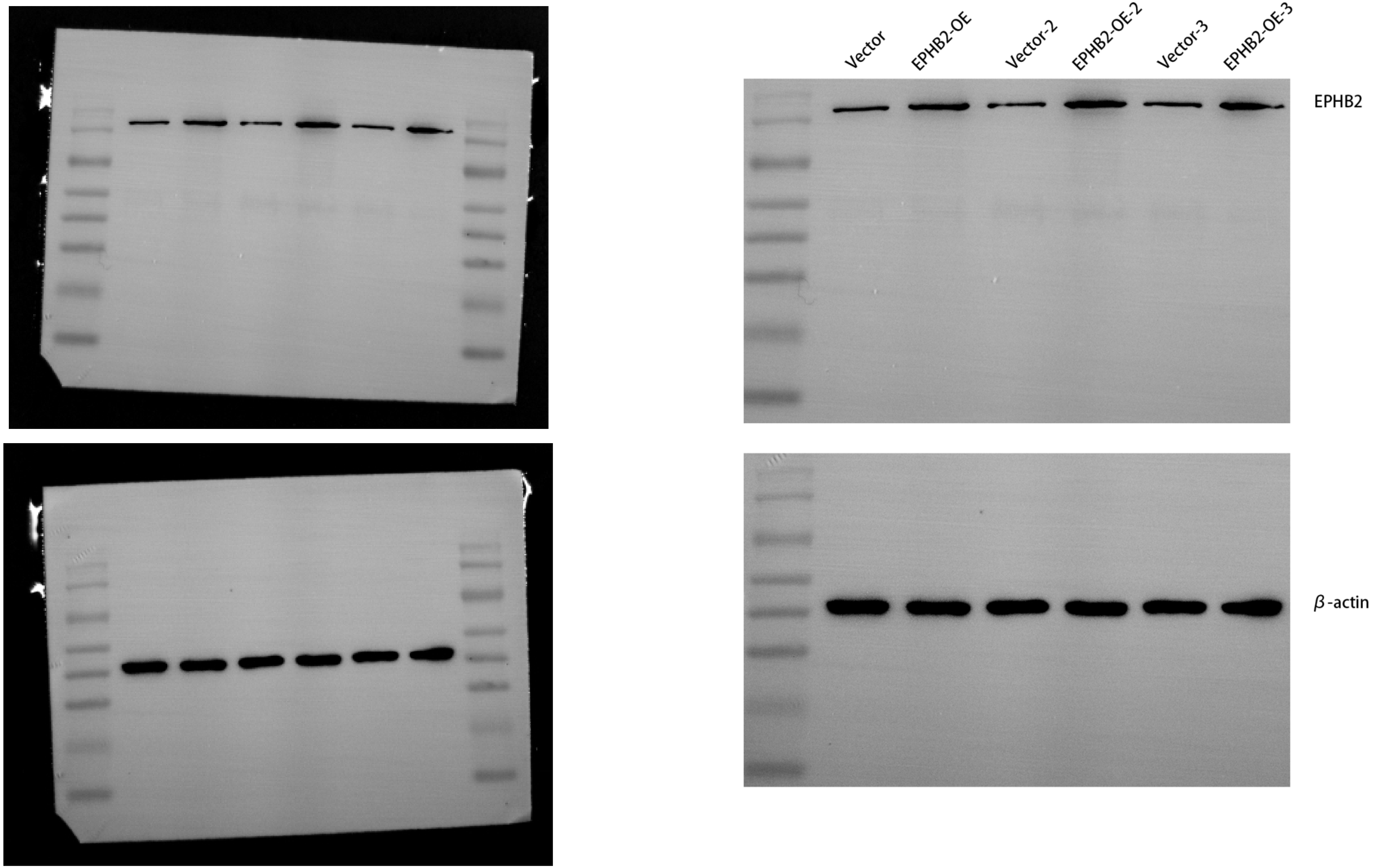

FIG3_A_Overexpression of EPHB2_SiHa

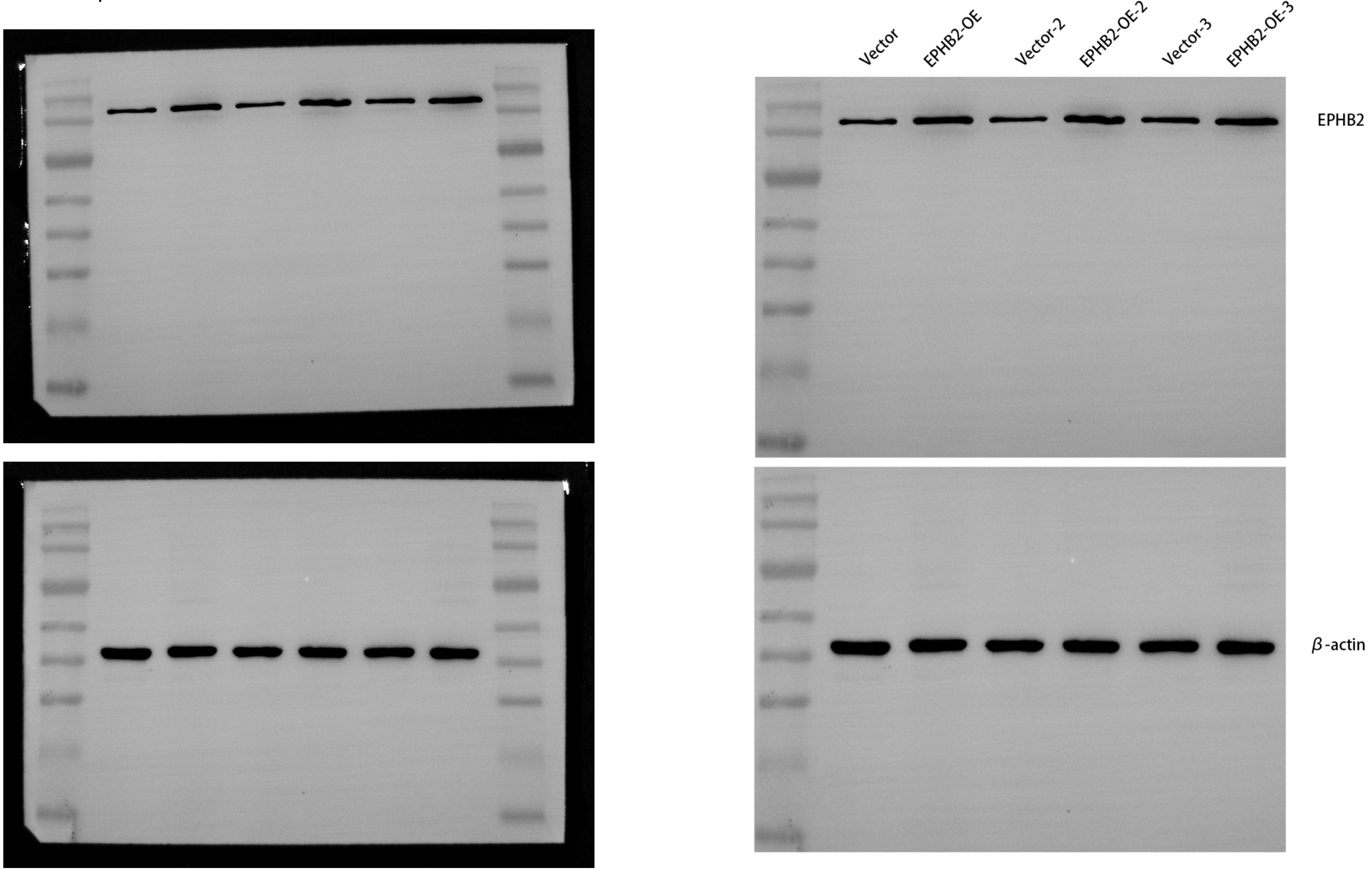

FIG3_G_ E-CAD_HeLa

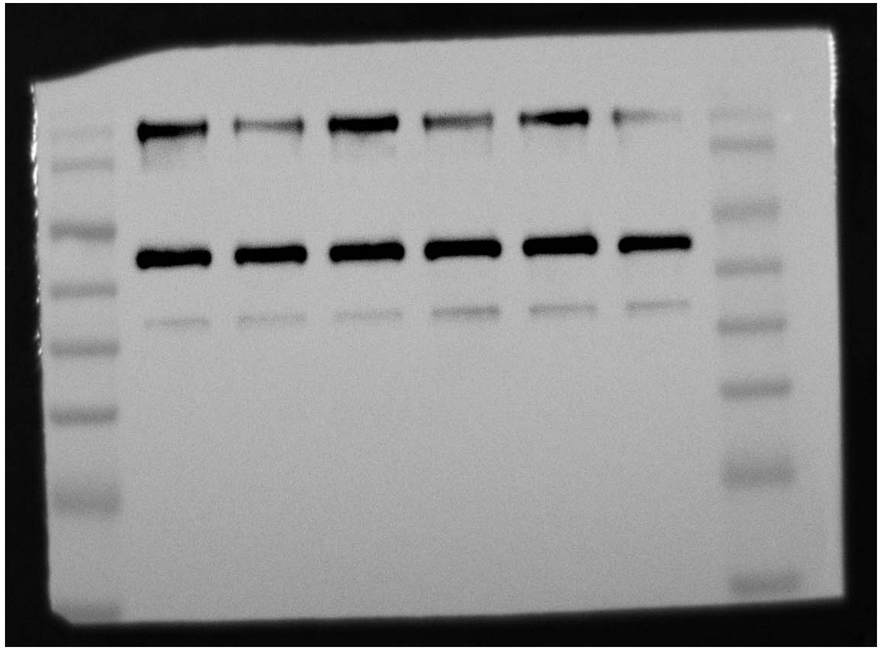

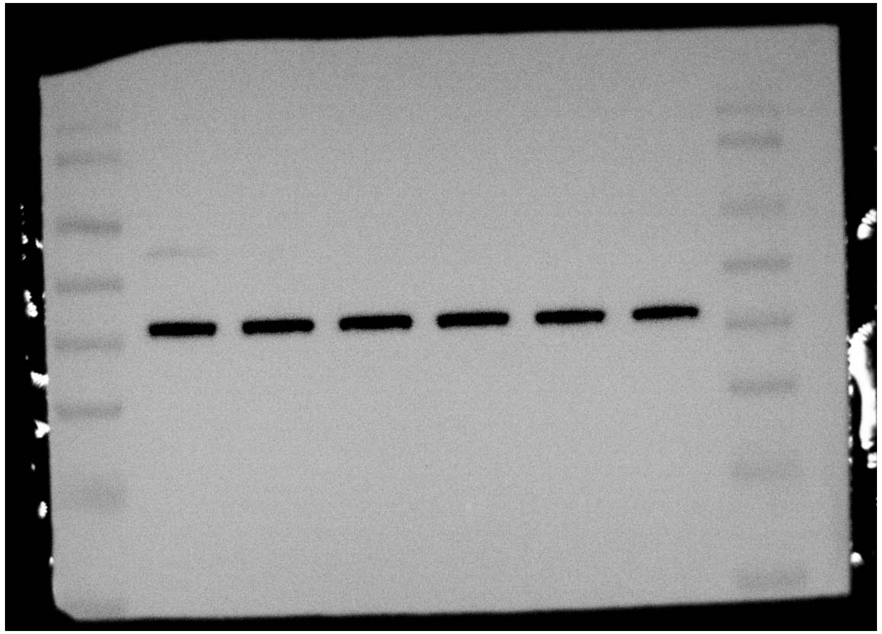

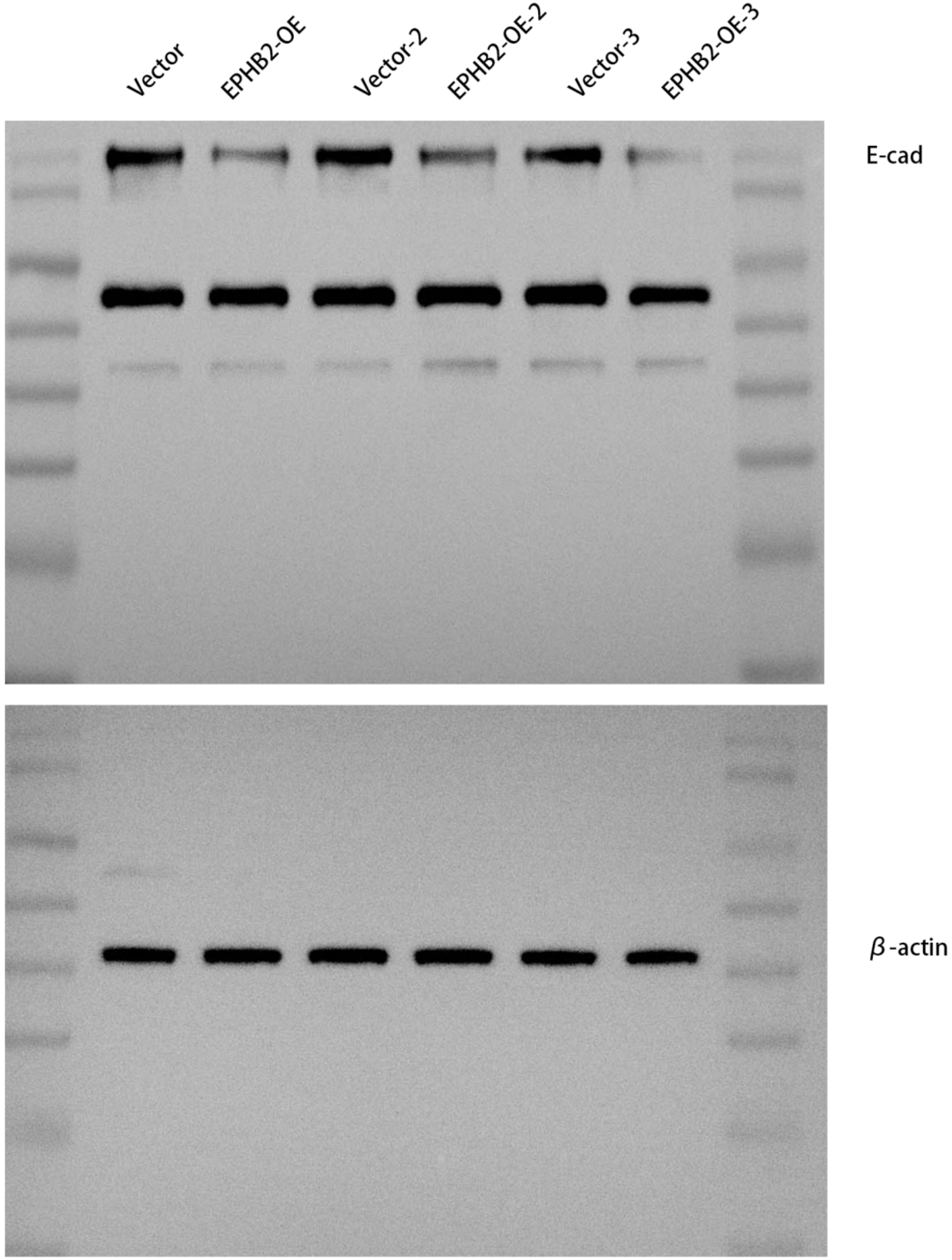

FIG3_G_ N-CAD_HeLa

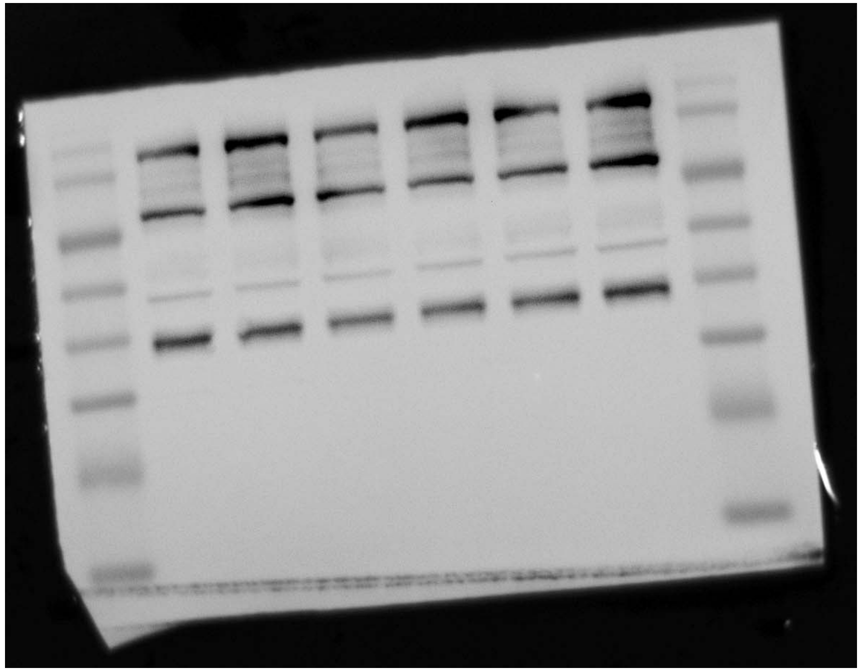

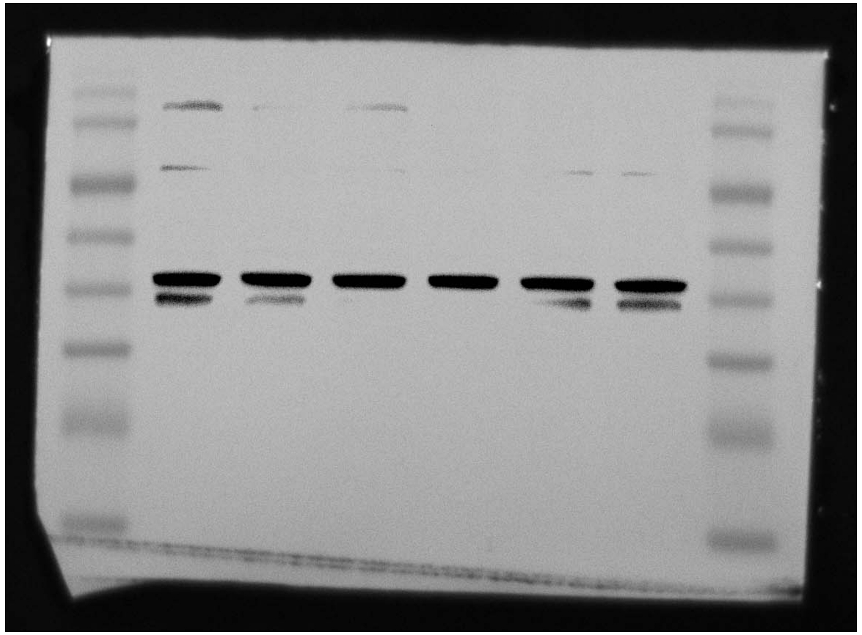

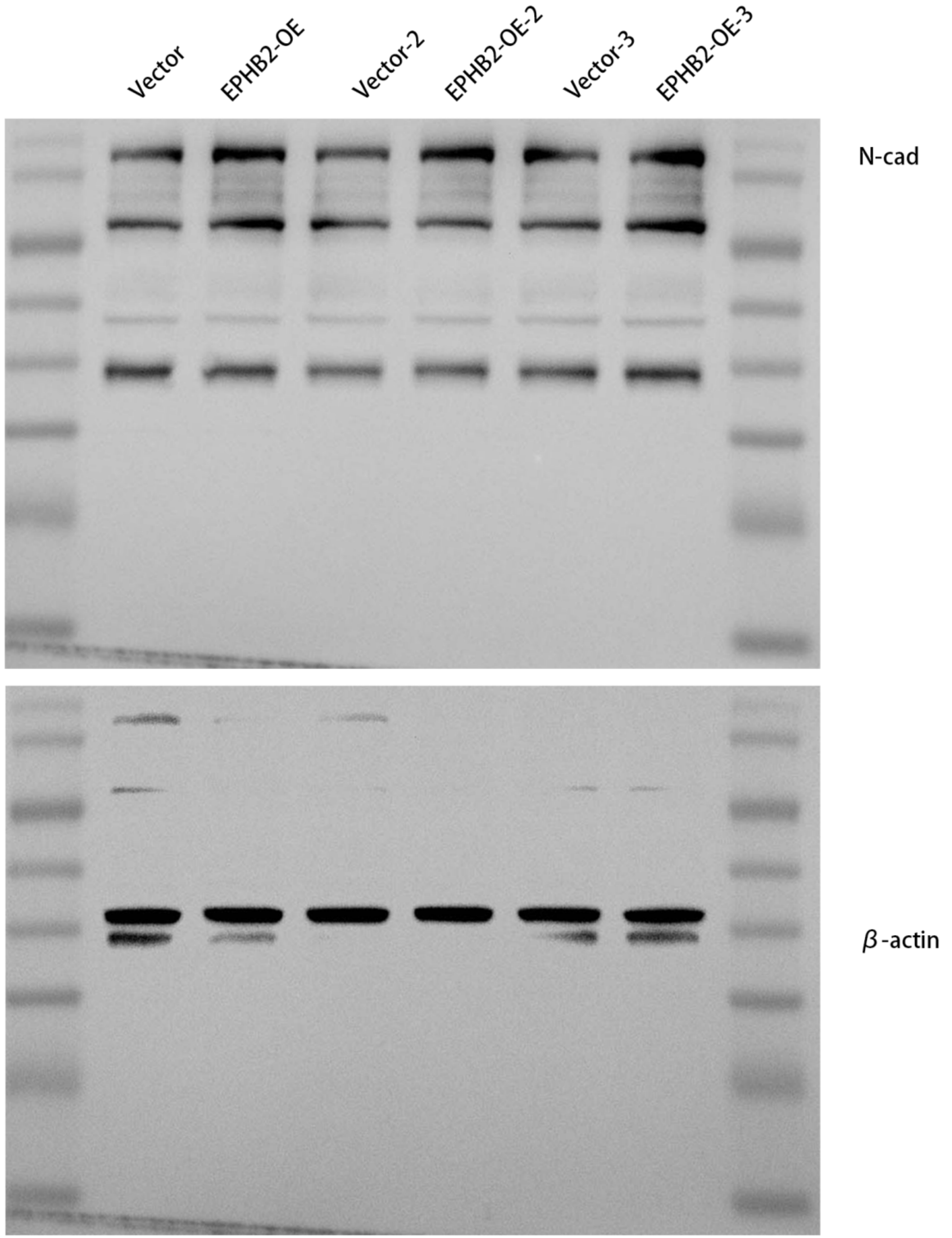

FIG3_G_ E-CAD_SiHa

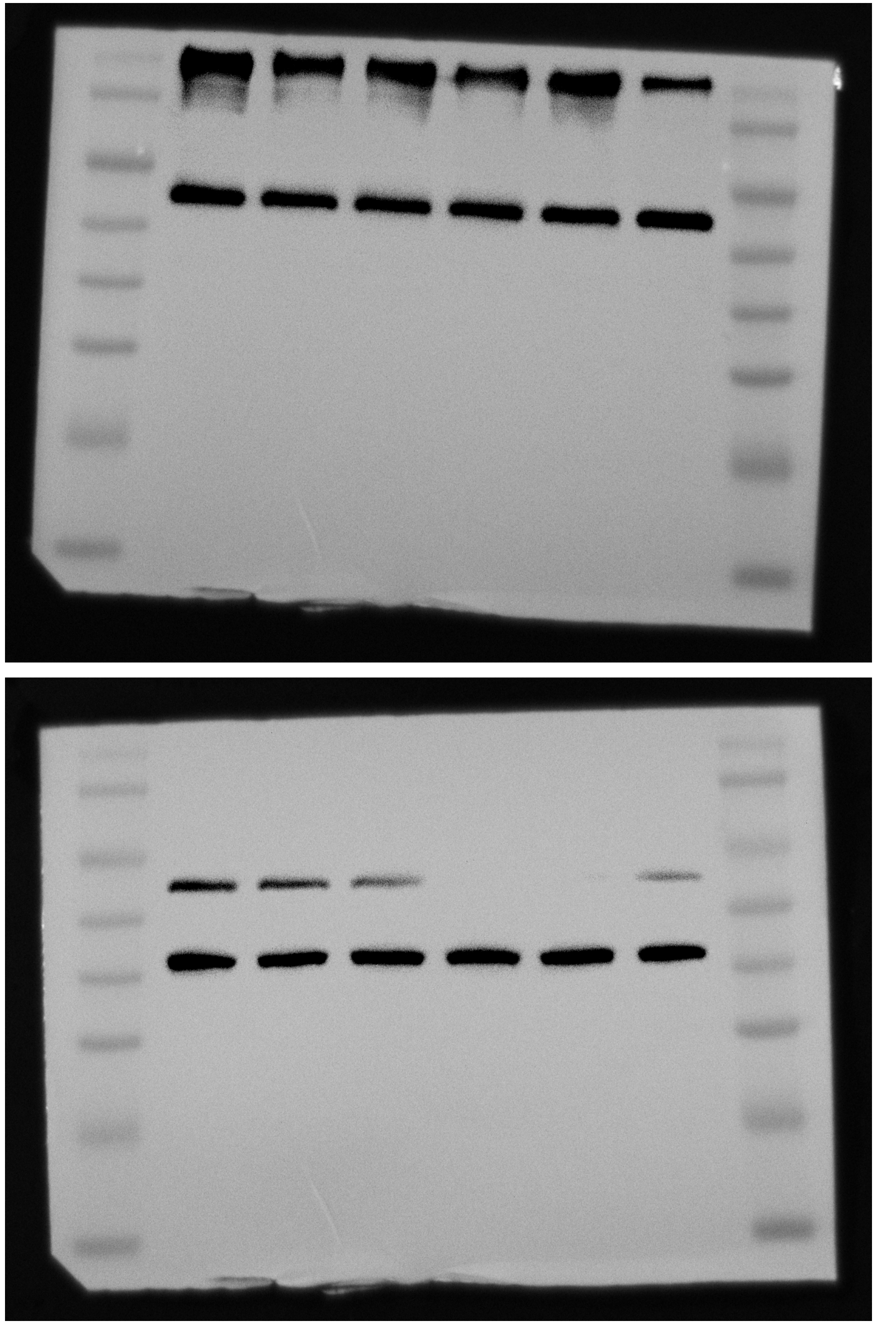

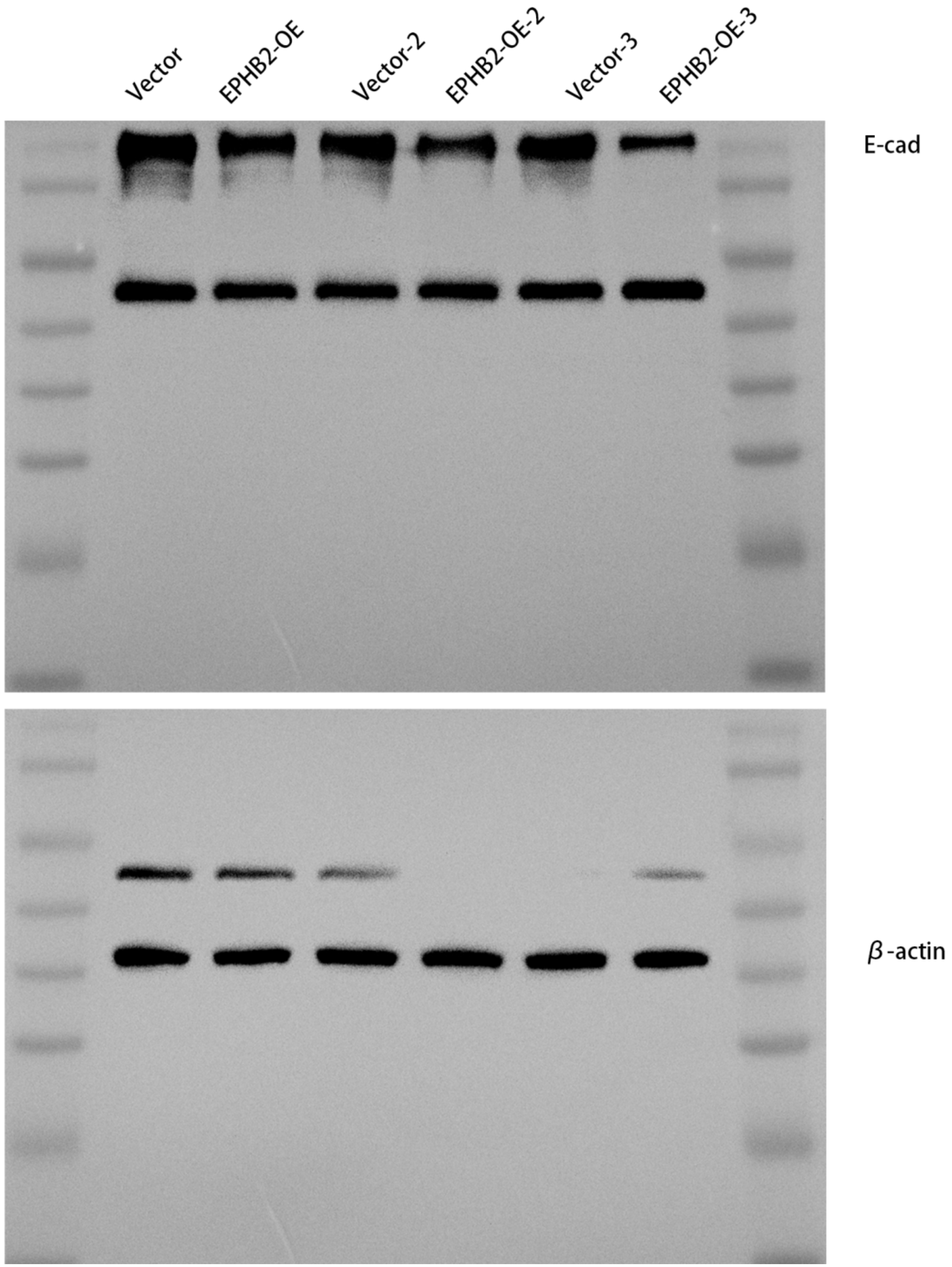

FIG3_G_ N-CAD_SiHa

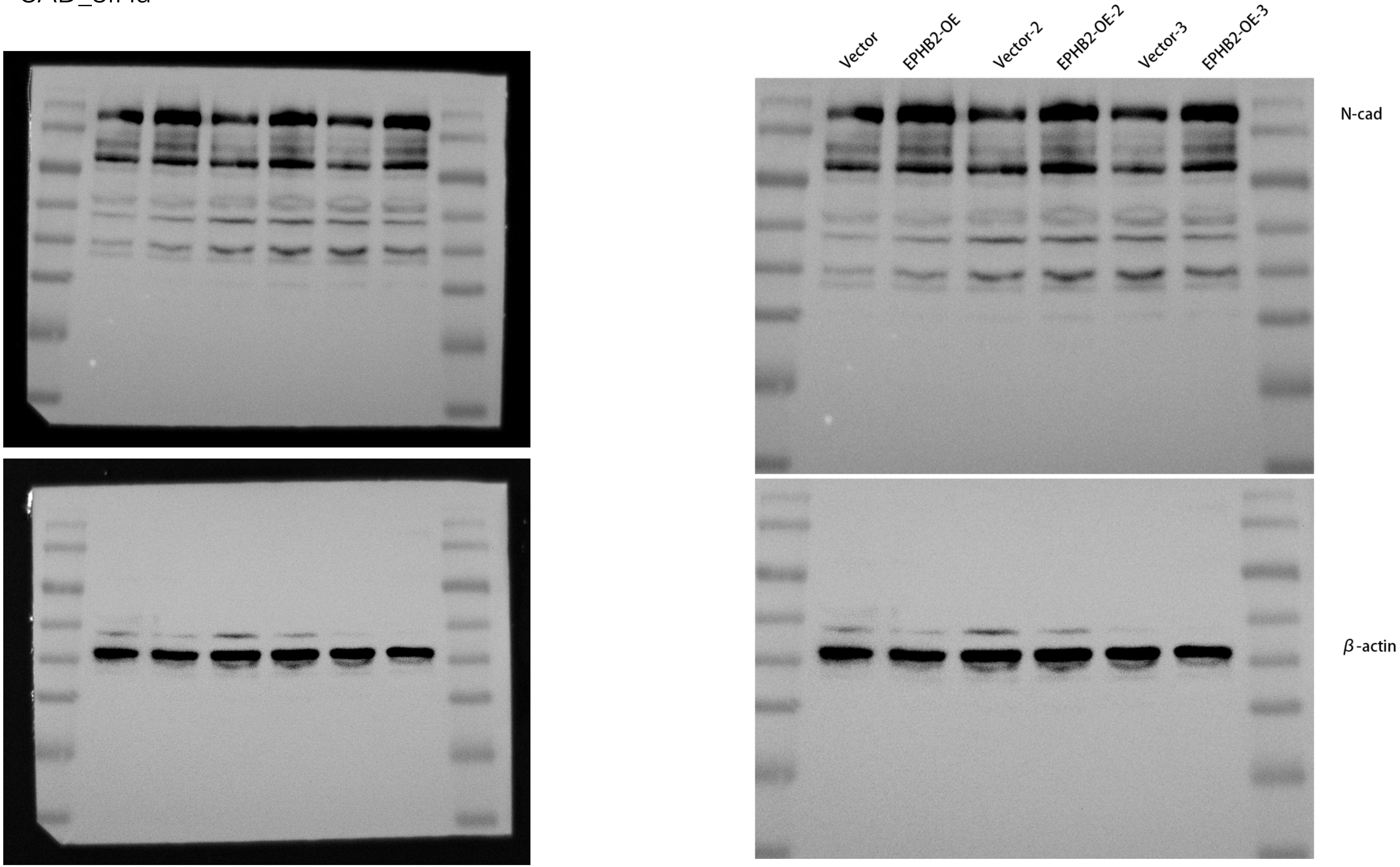

FIG5_B_SiHa_sh

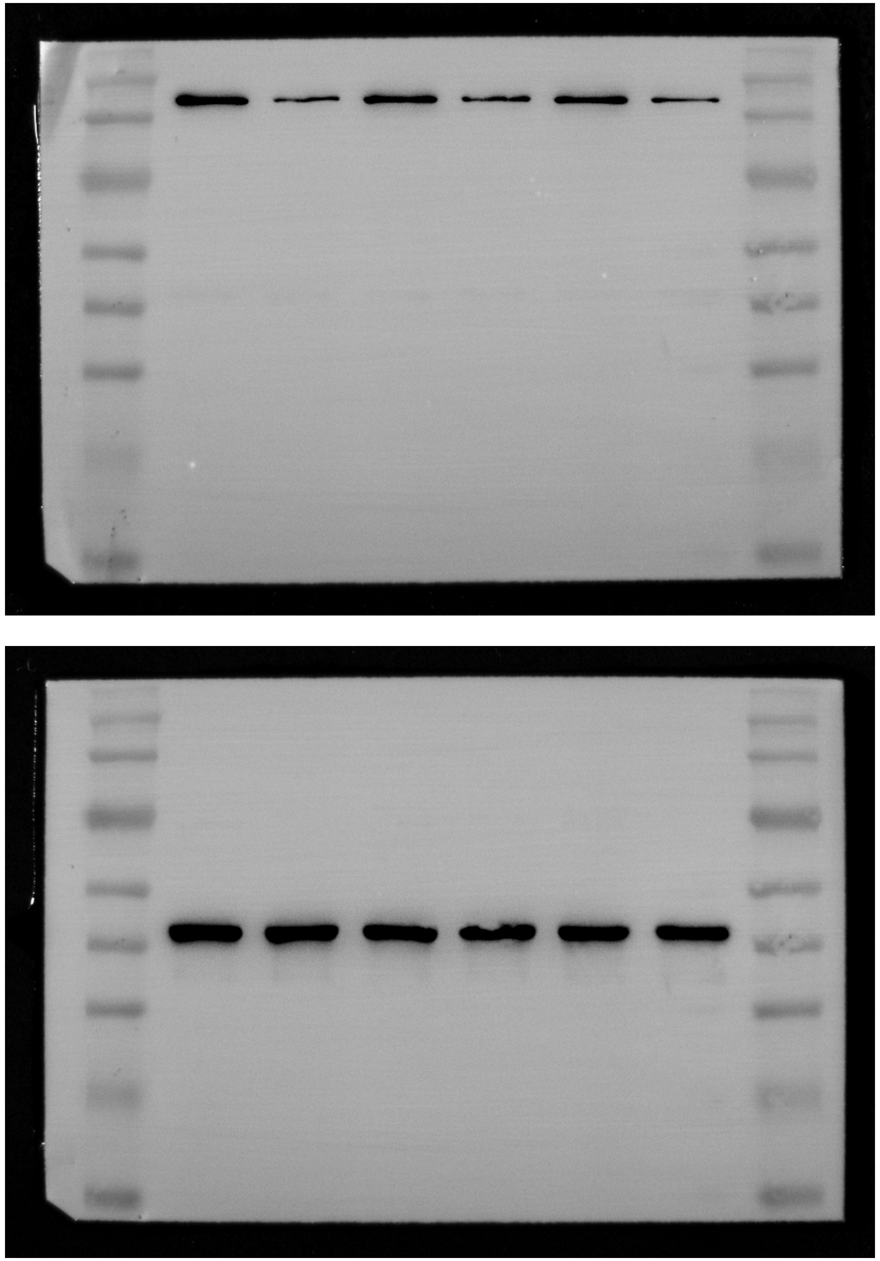

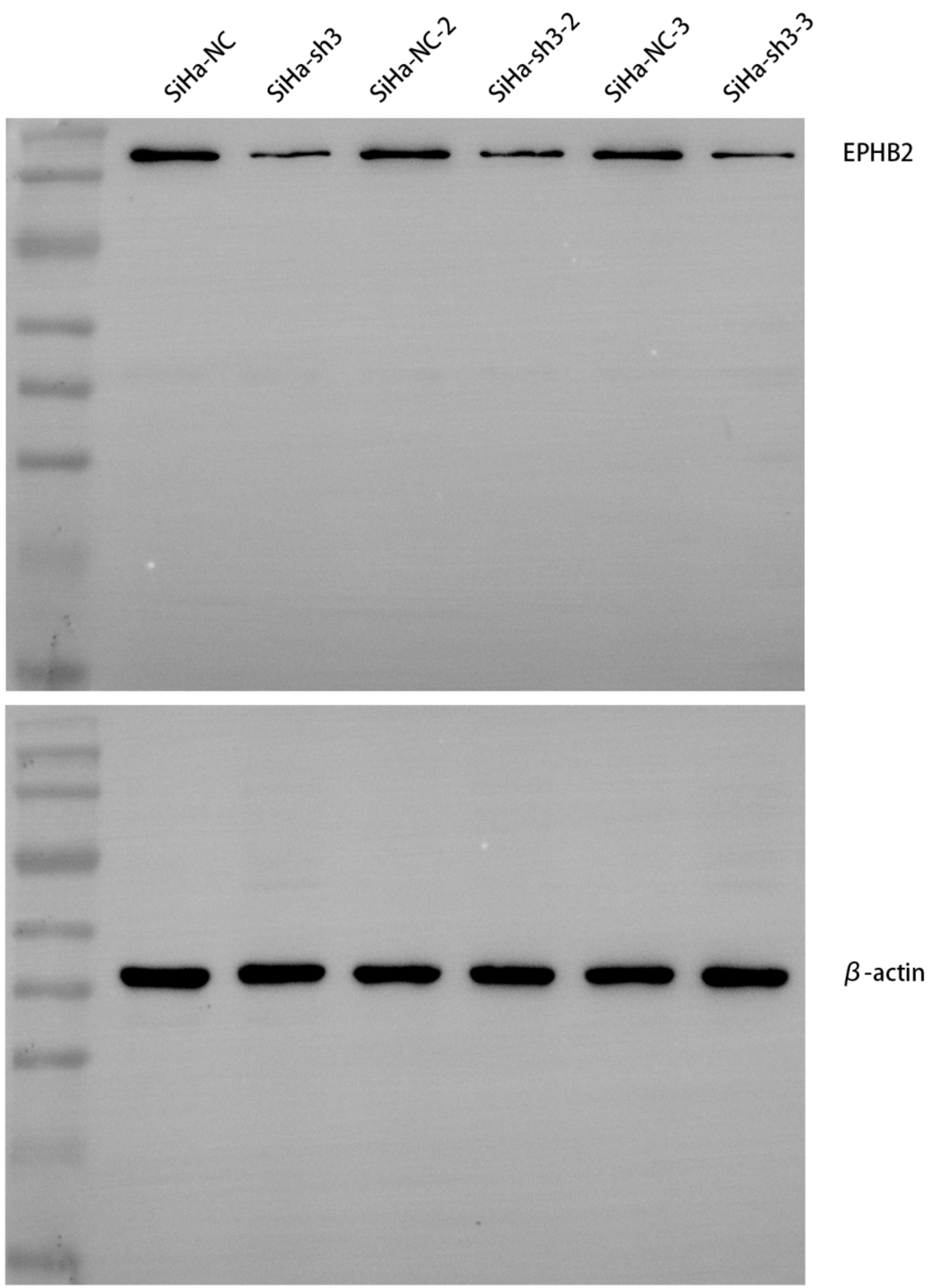

FIG6_G_HeLa_ETV4

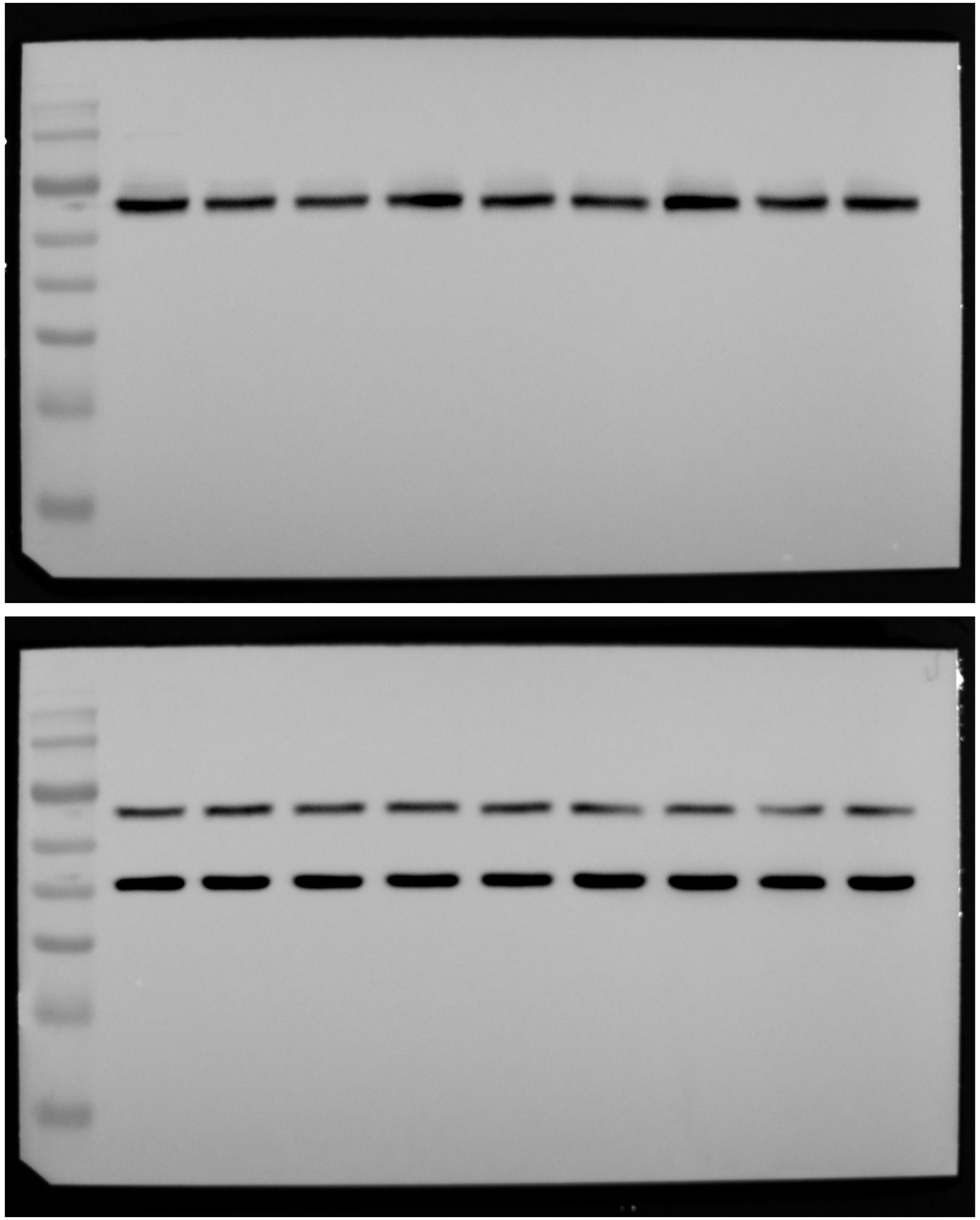

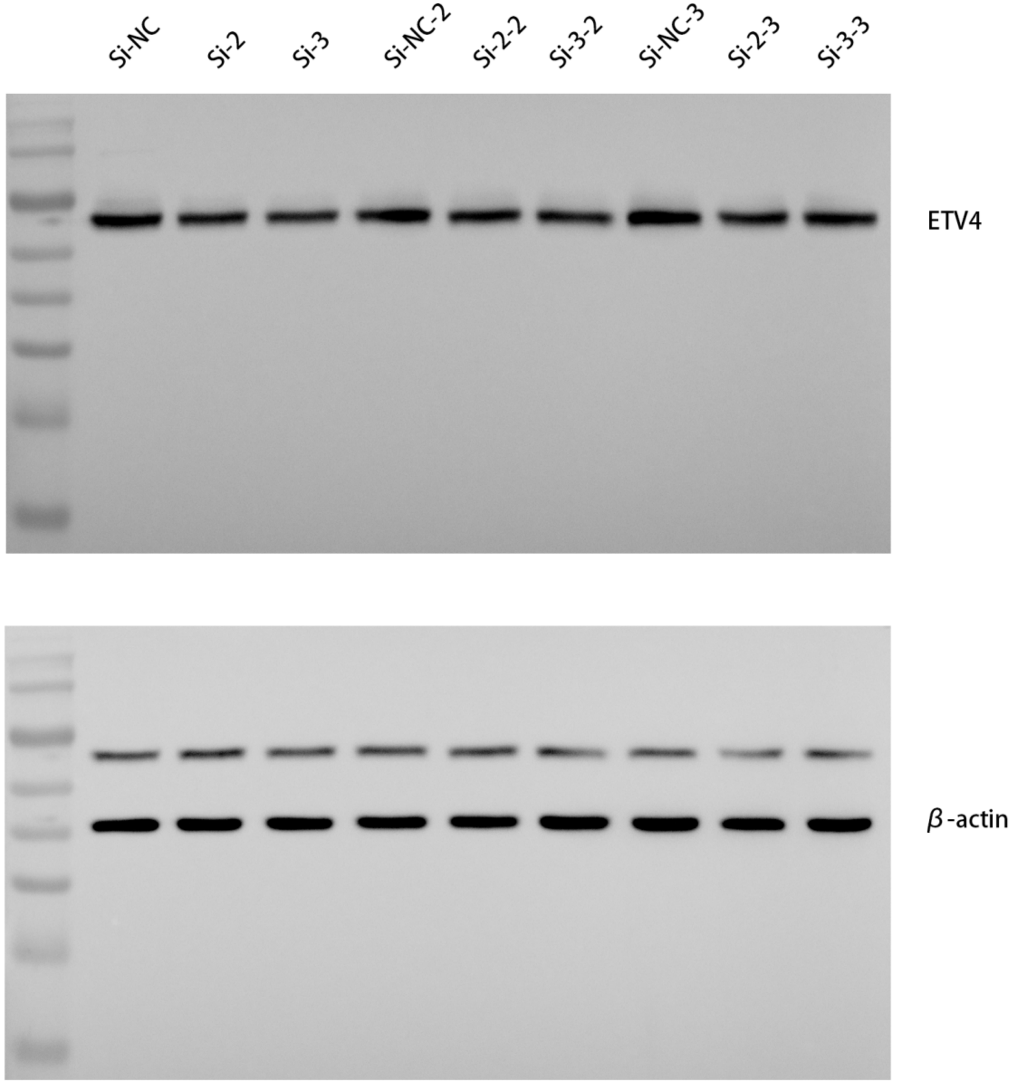

FIG6_G_HeLa_CXCL8

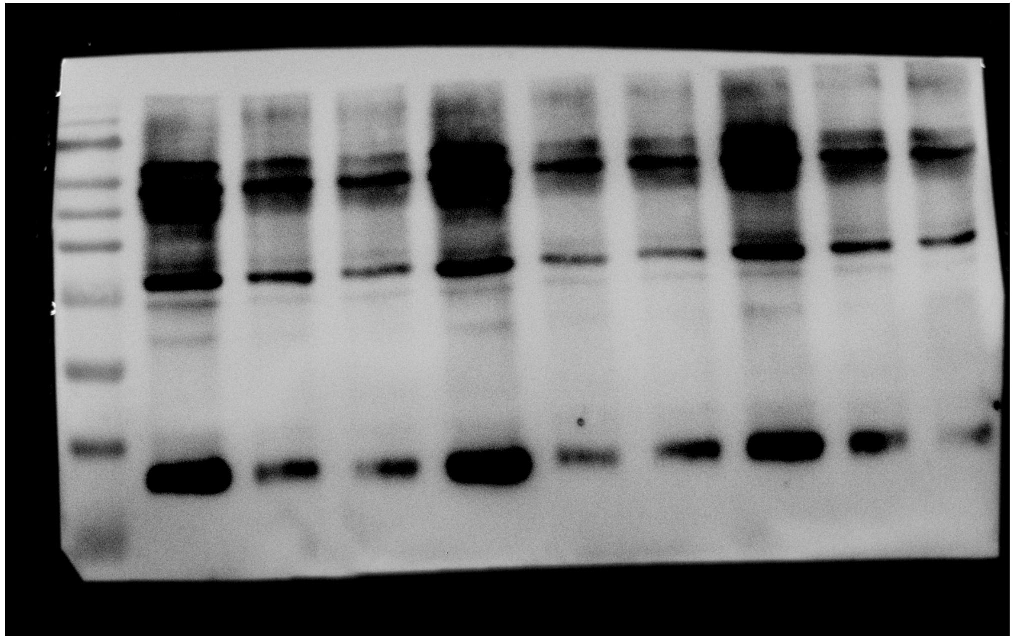

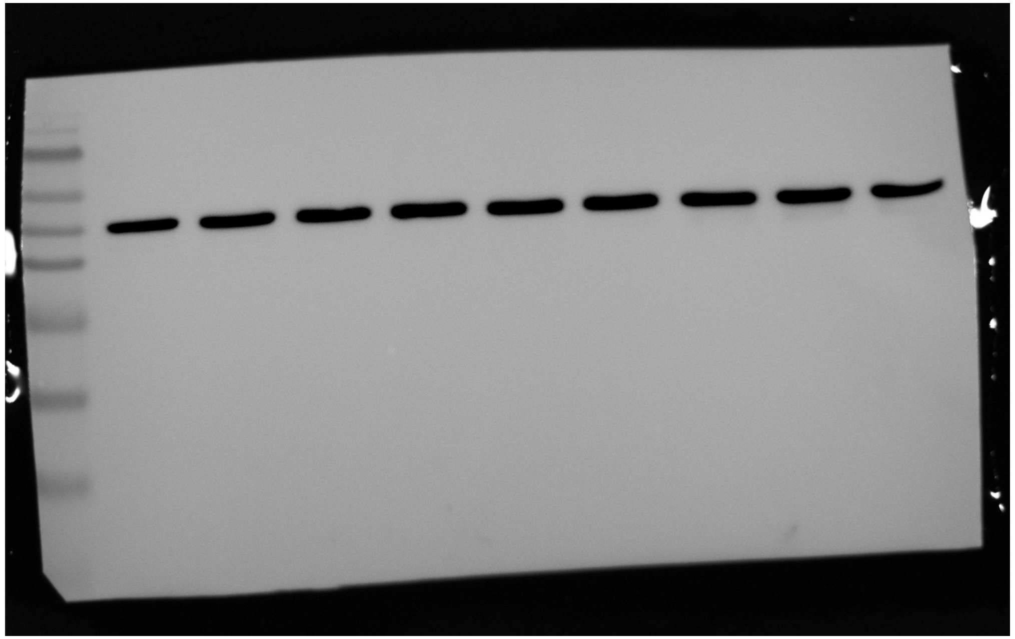

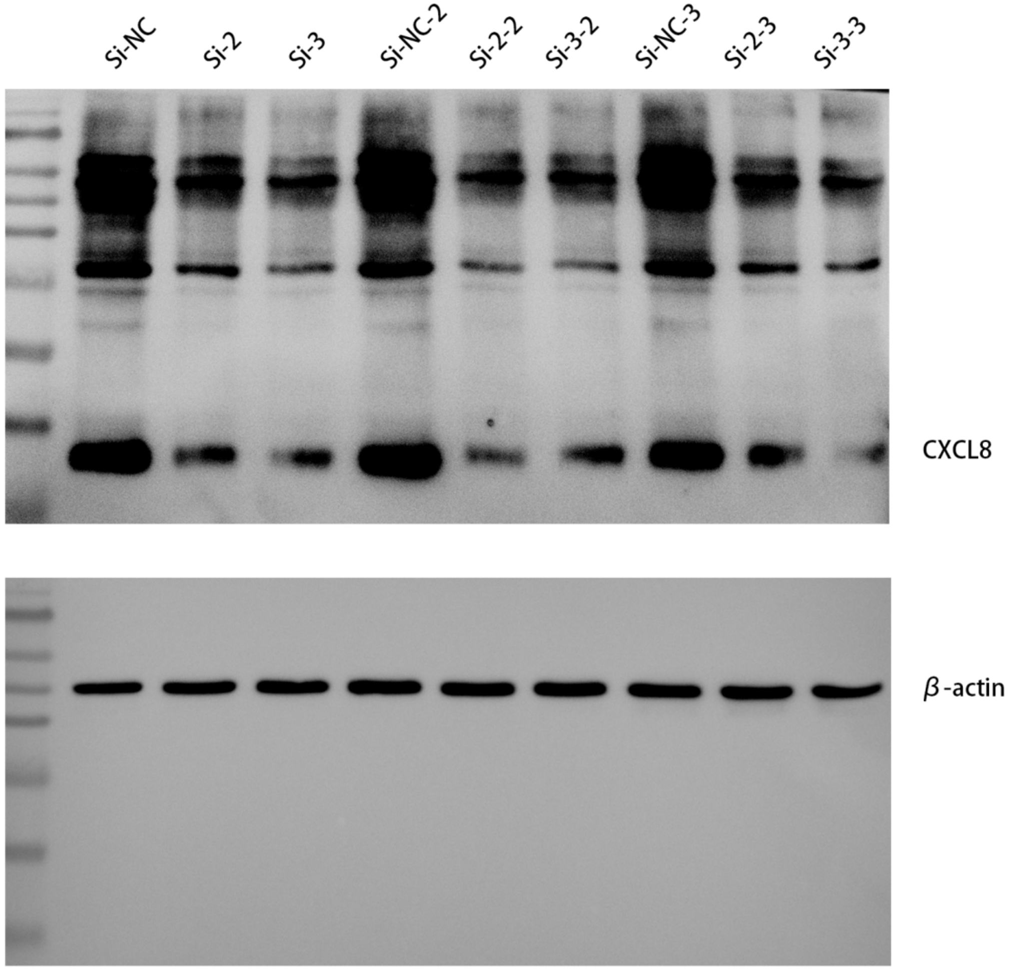

FIG6_G_HeLa_DUSP6

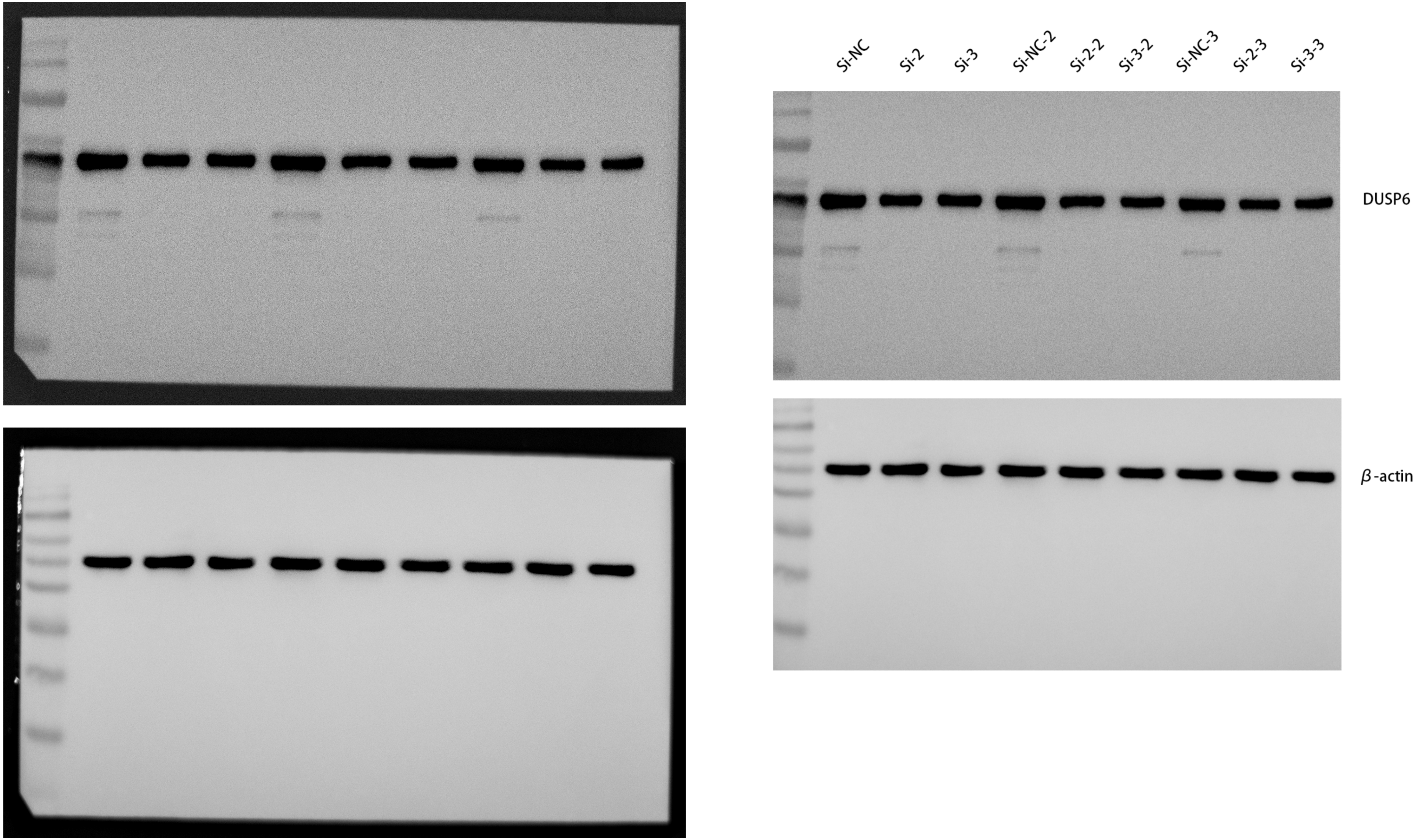

FIG6_H_SiHa_ETV4

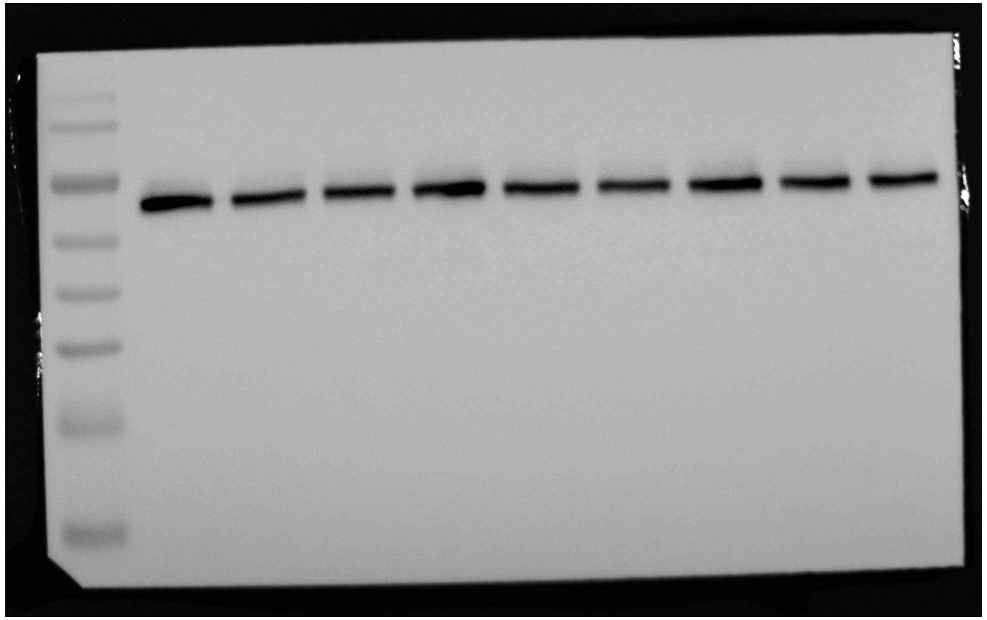

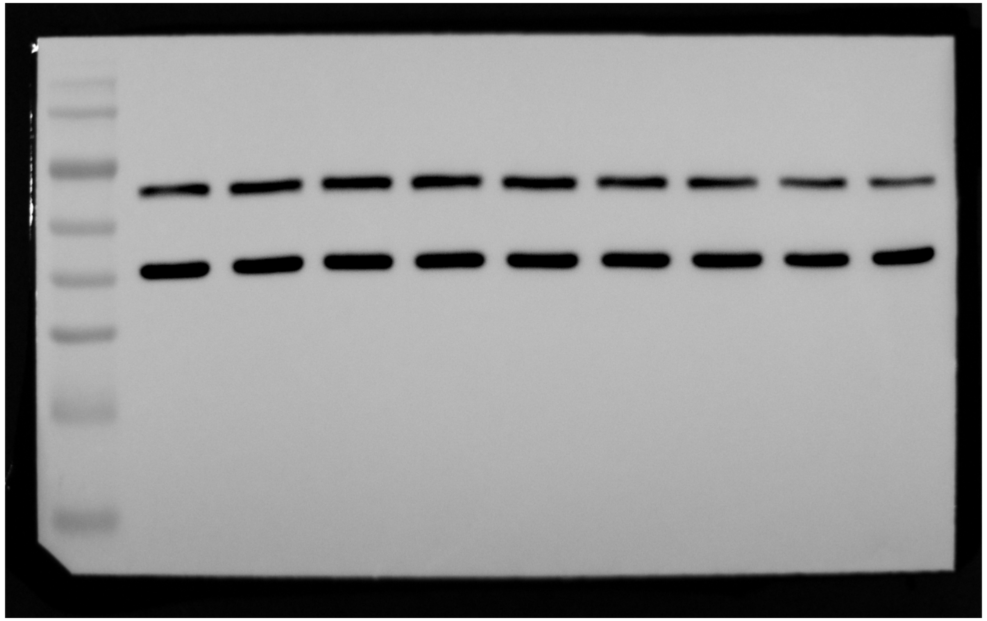

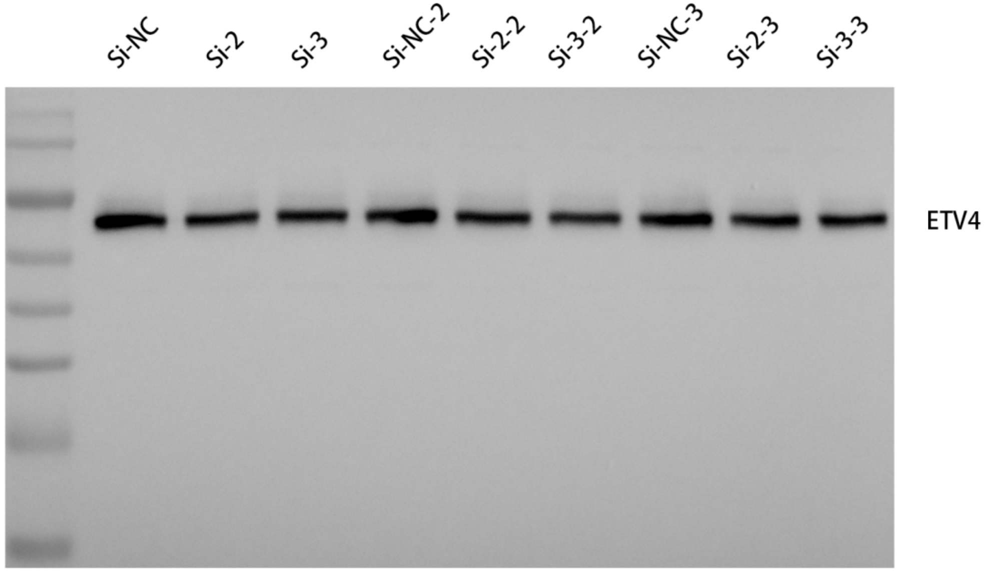

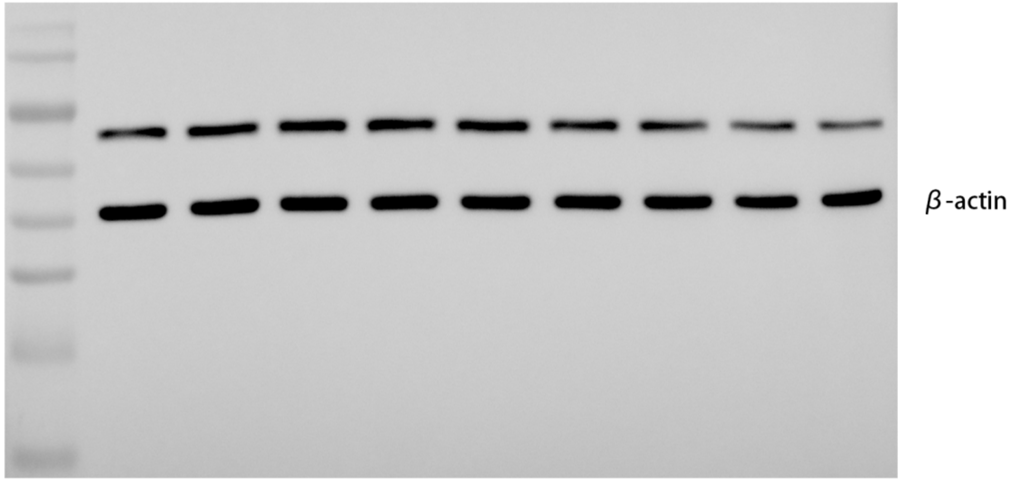

FIG6_H_SiHa_CXCL8

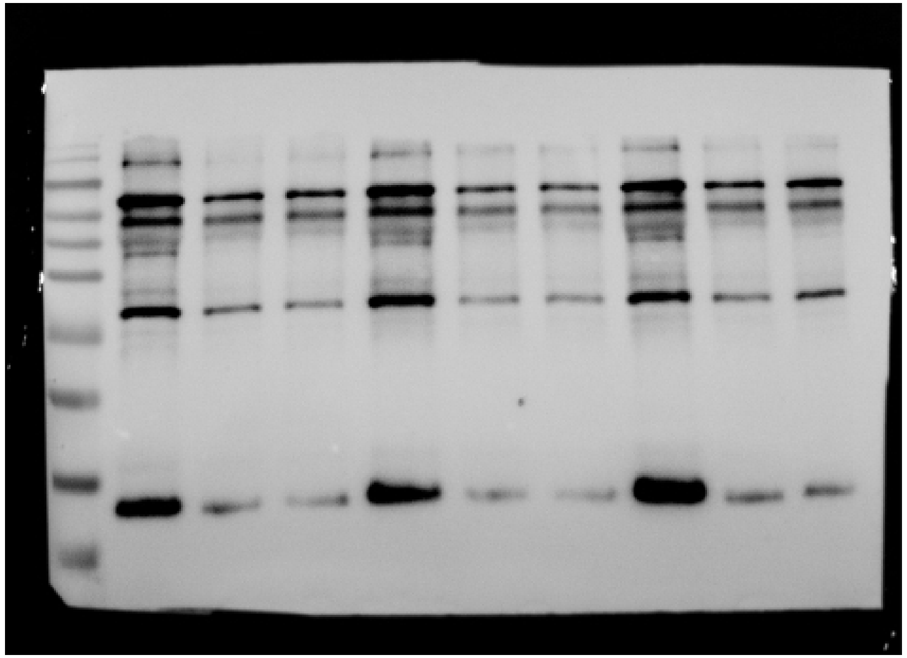

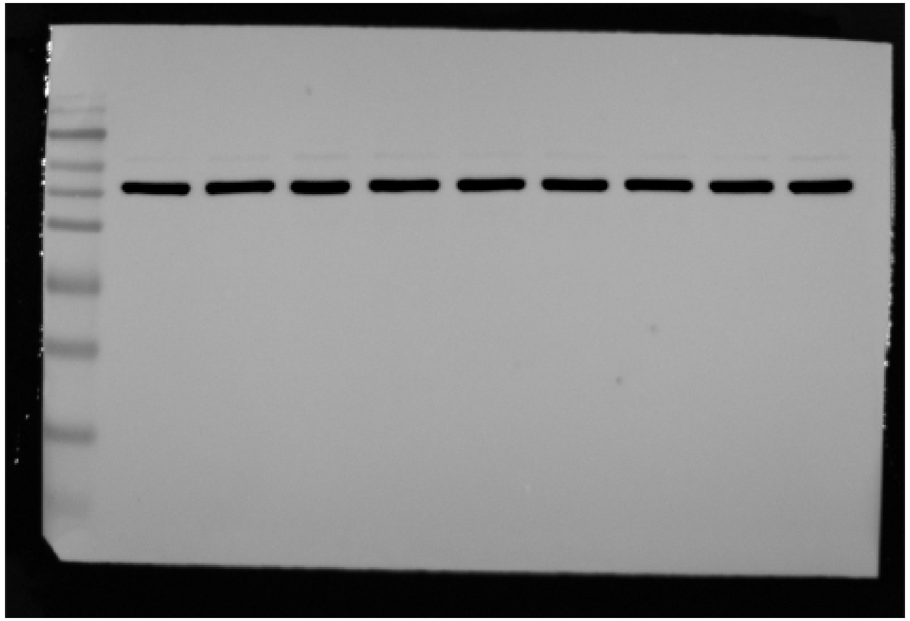

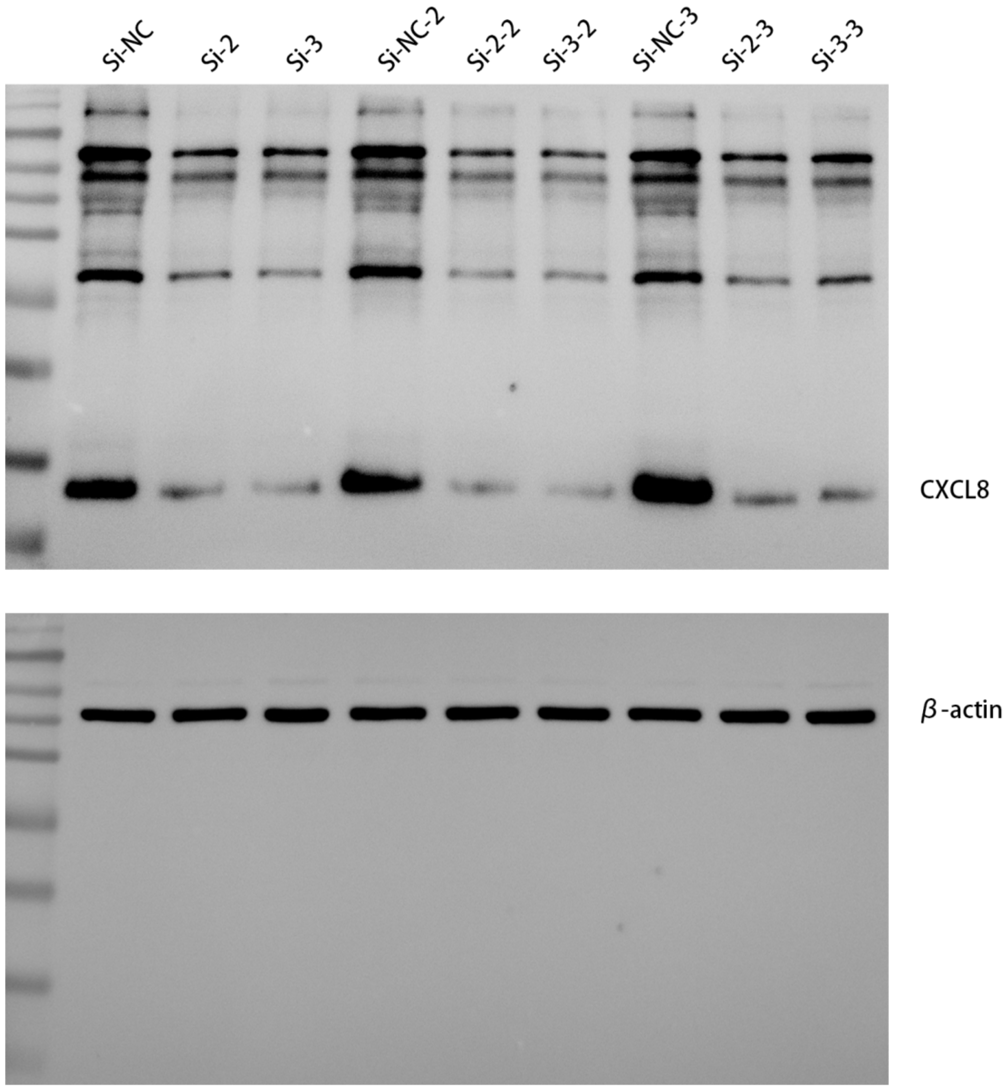

FIG6_H_SiHa_DUSP6

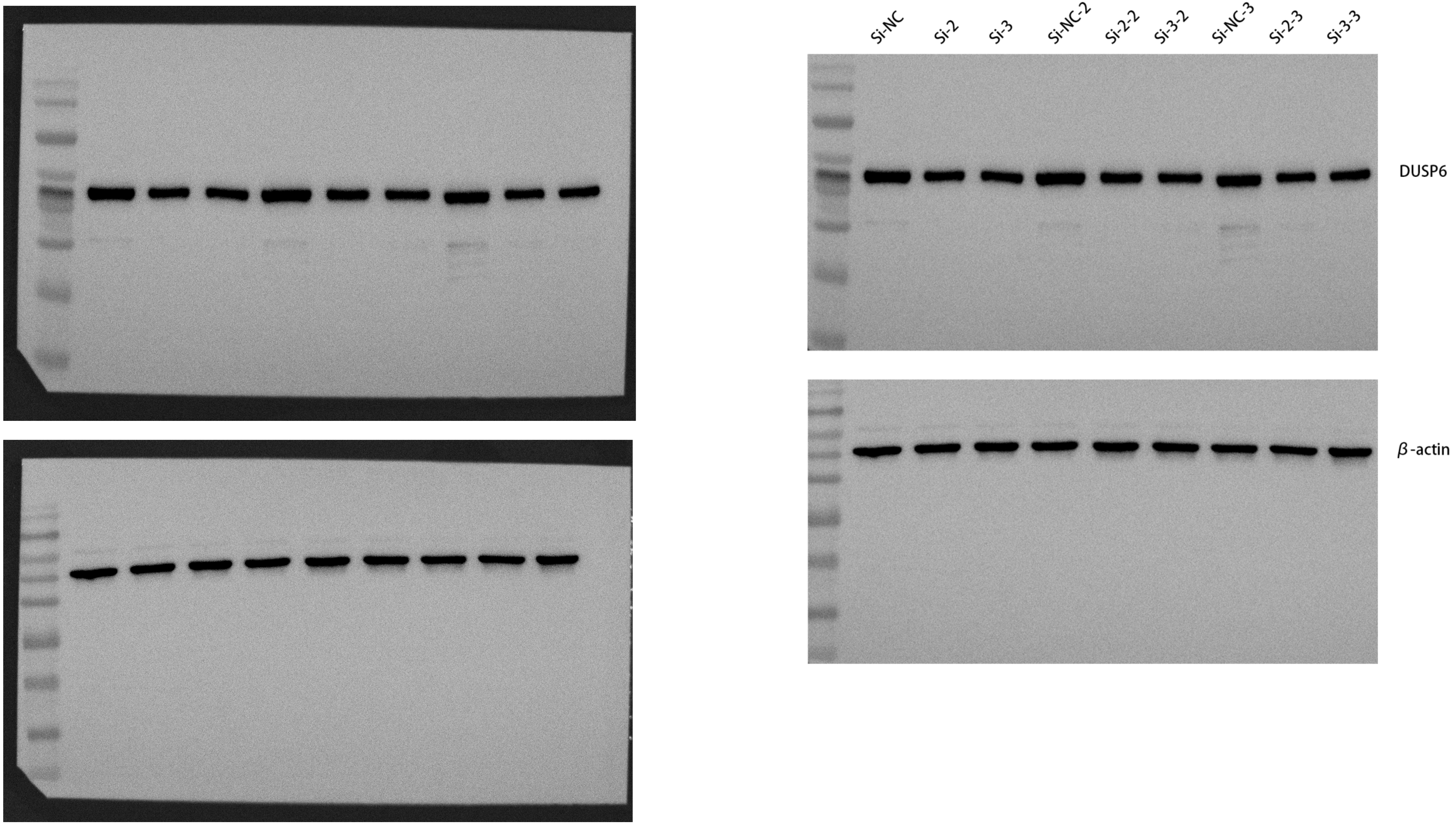

FIG6_I_HeLa_ERK

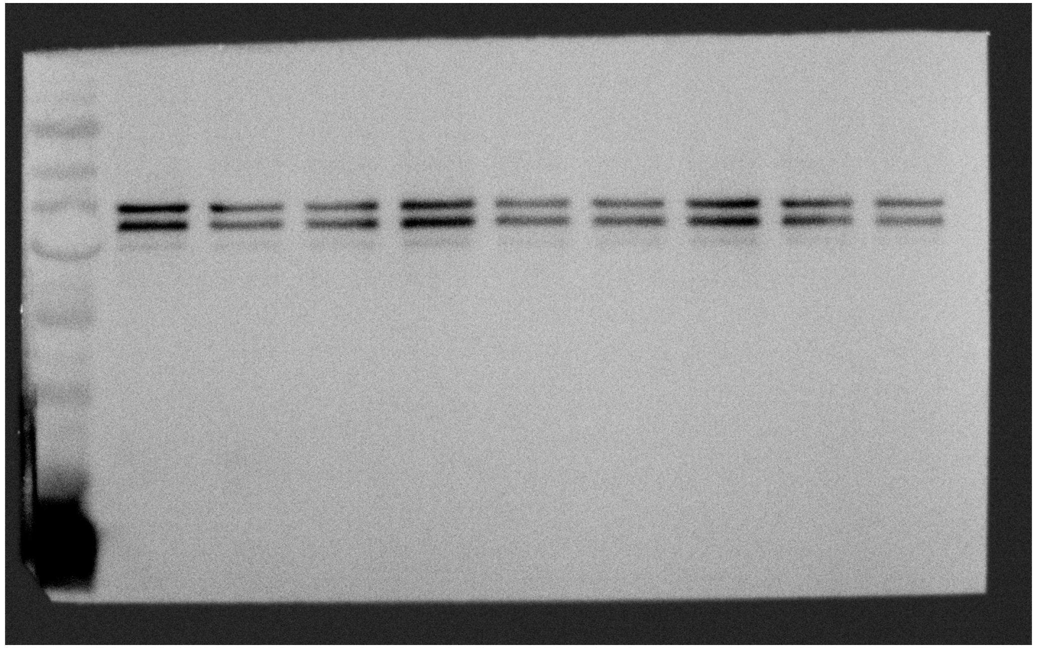

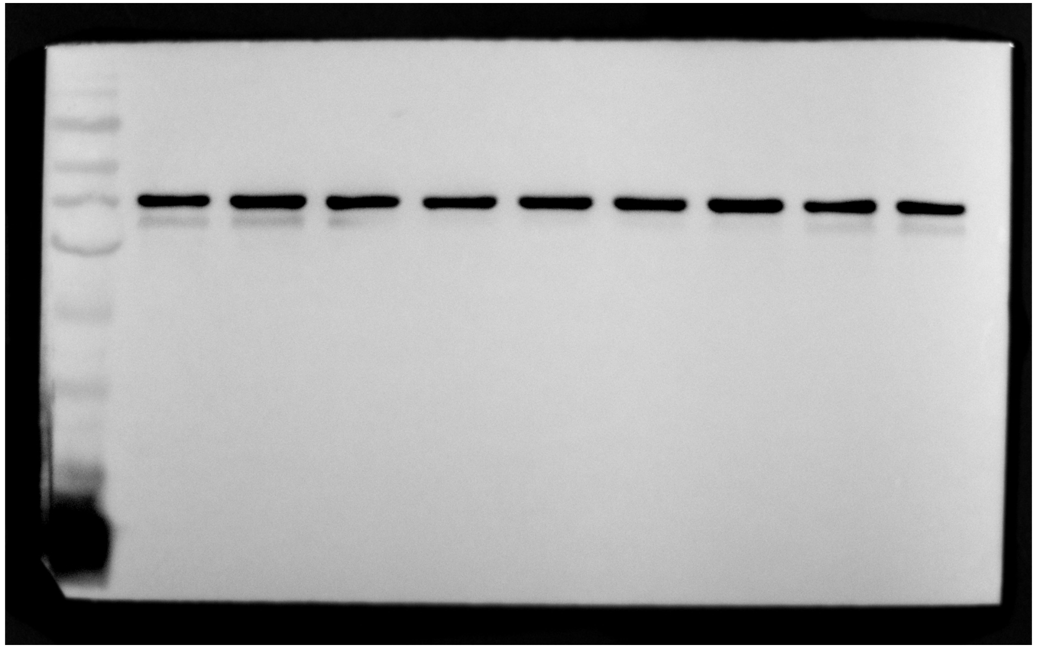

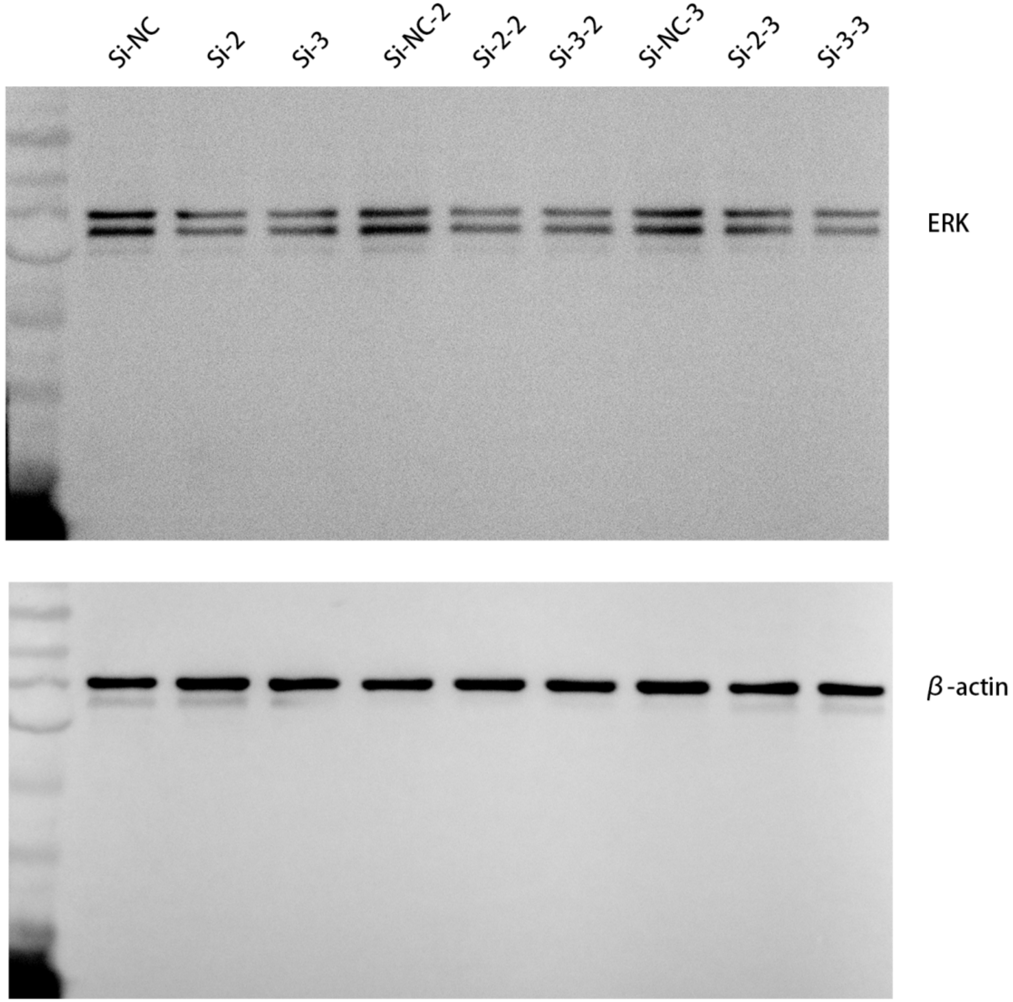

FIG6_I_HeLa_p-ERK

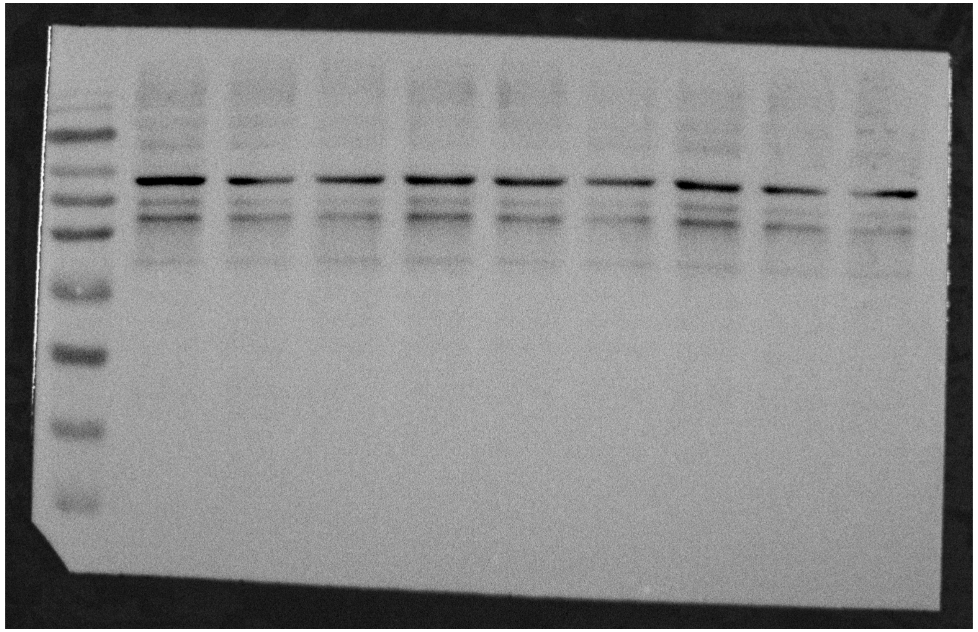

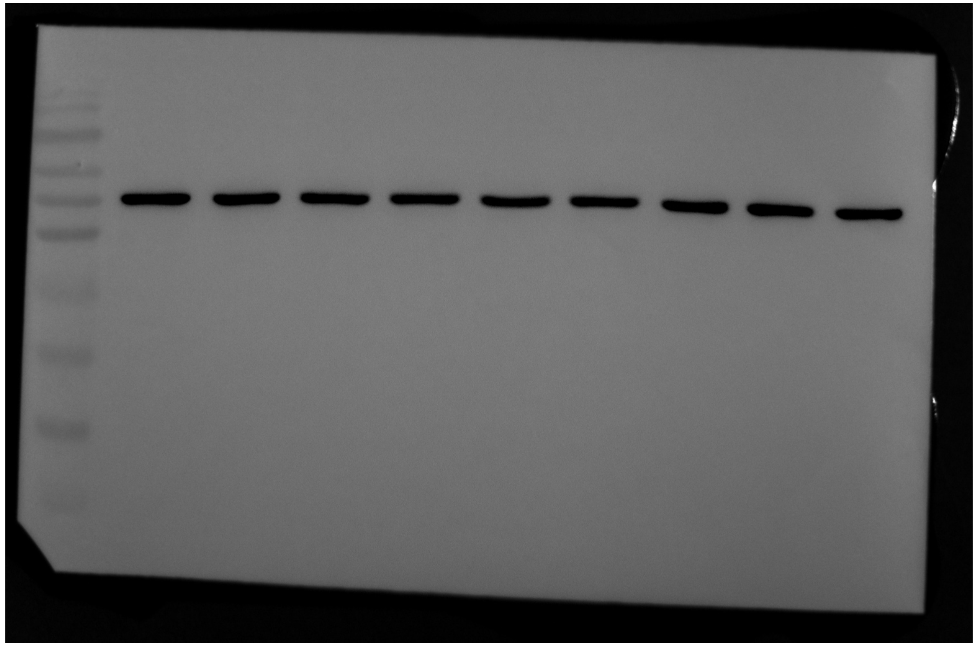

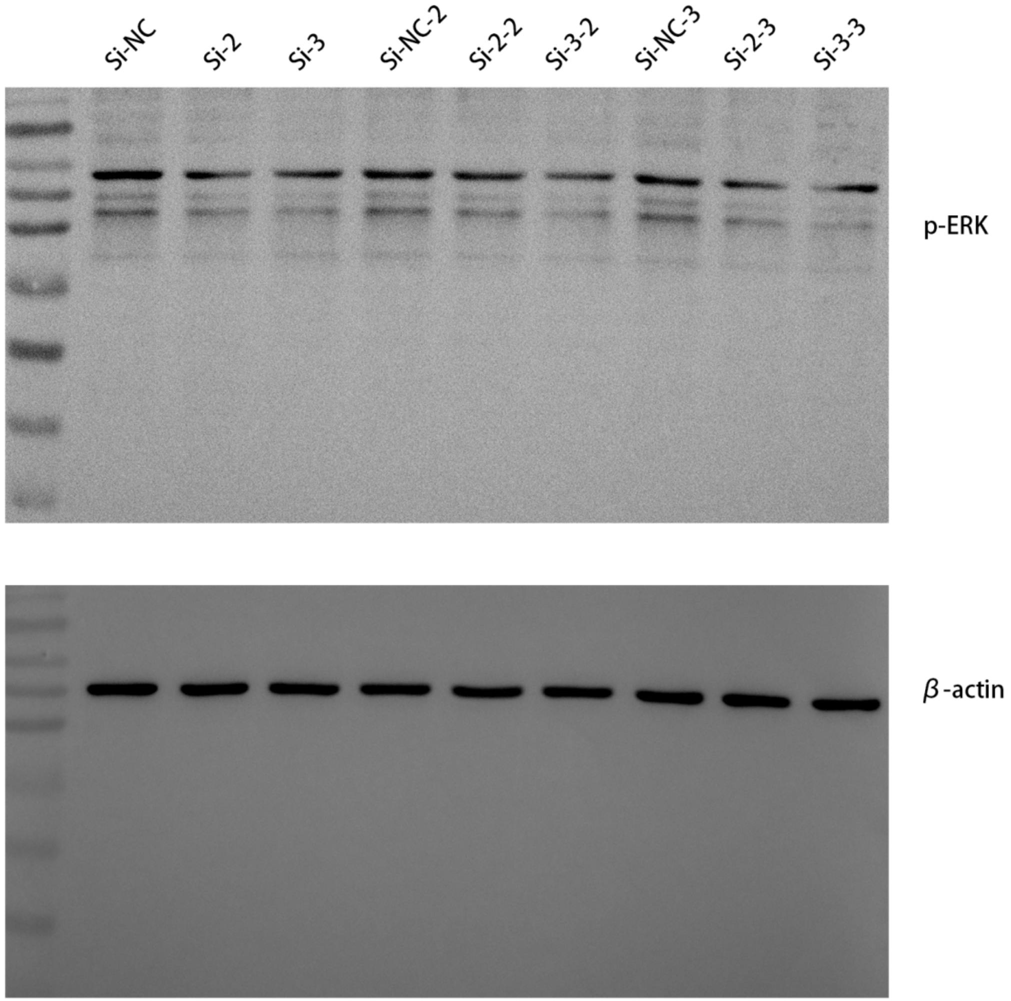

FIG6_I_SiHa_ERK

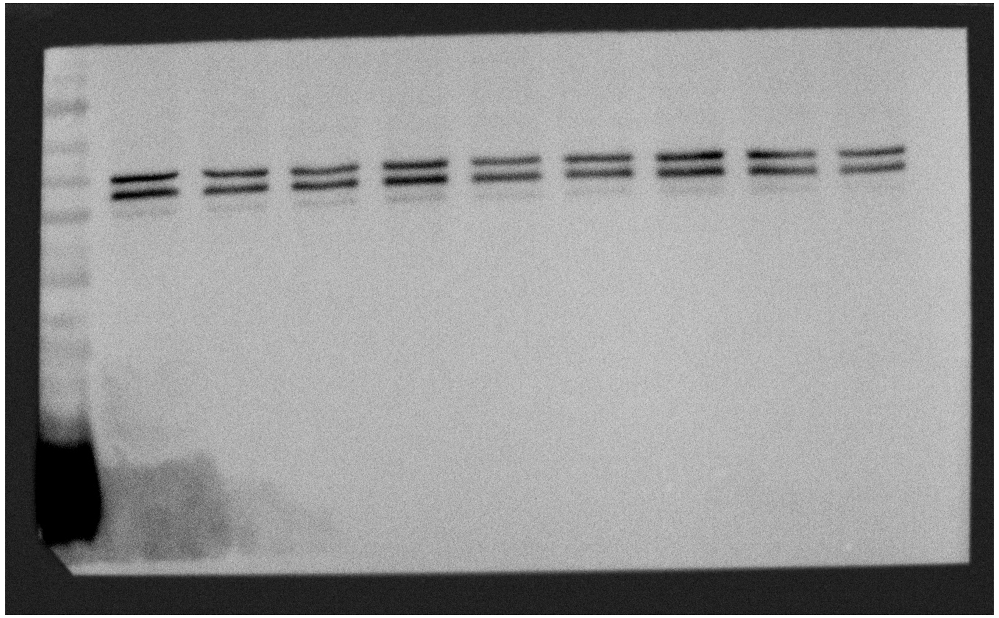

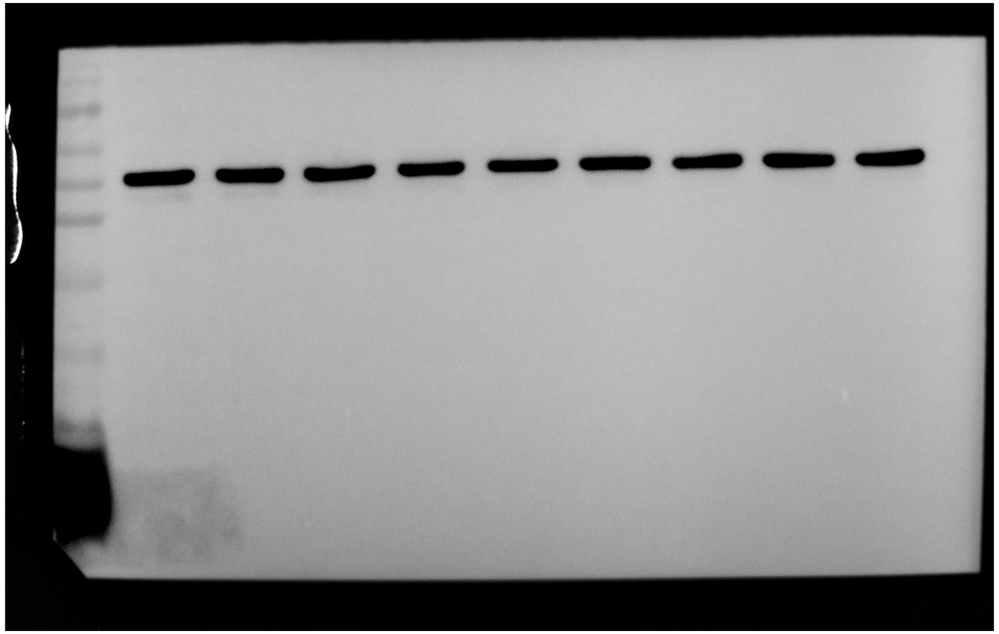

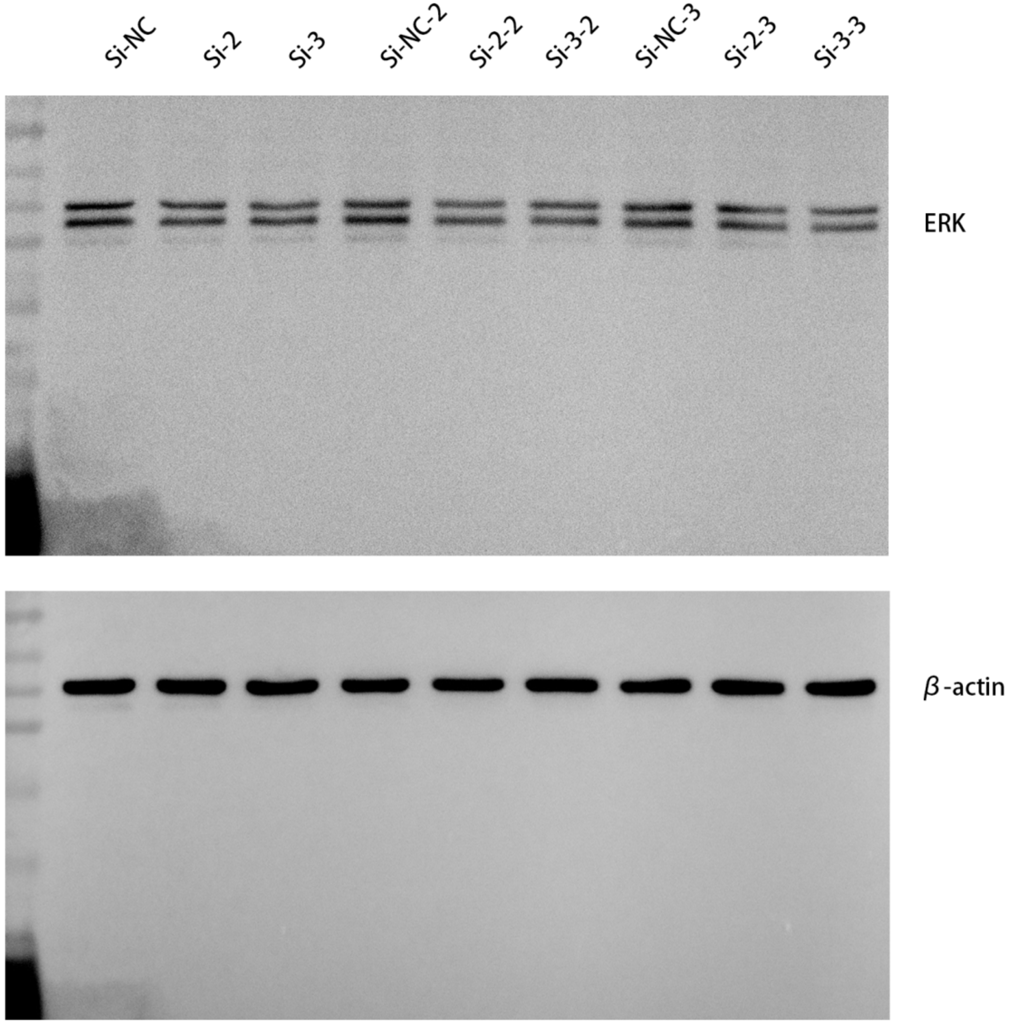

FIG6_I_SiHa_p-ERK

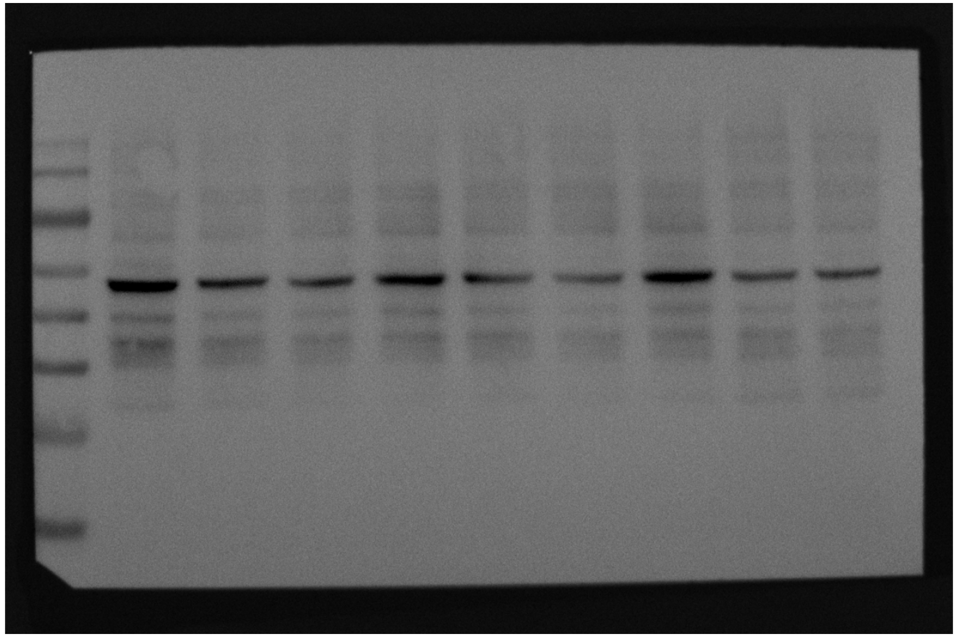

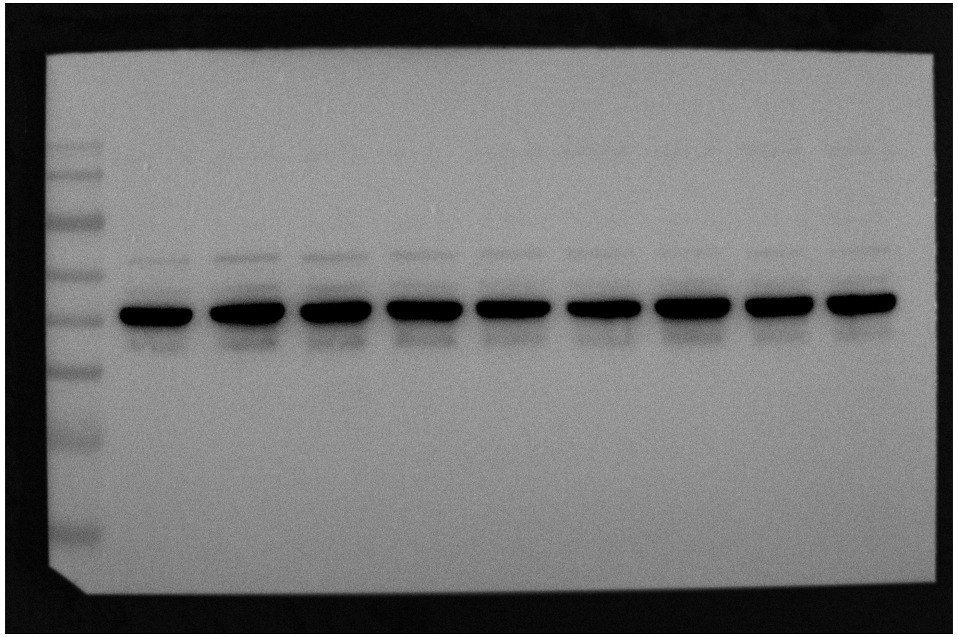

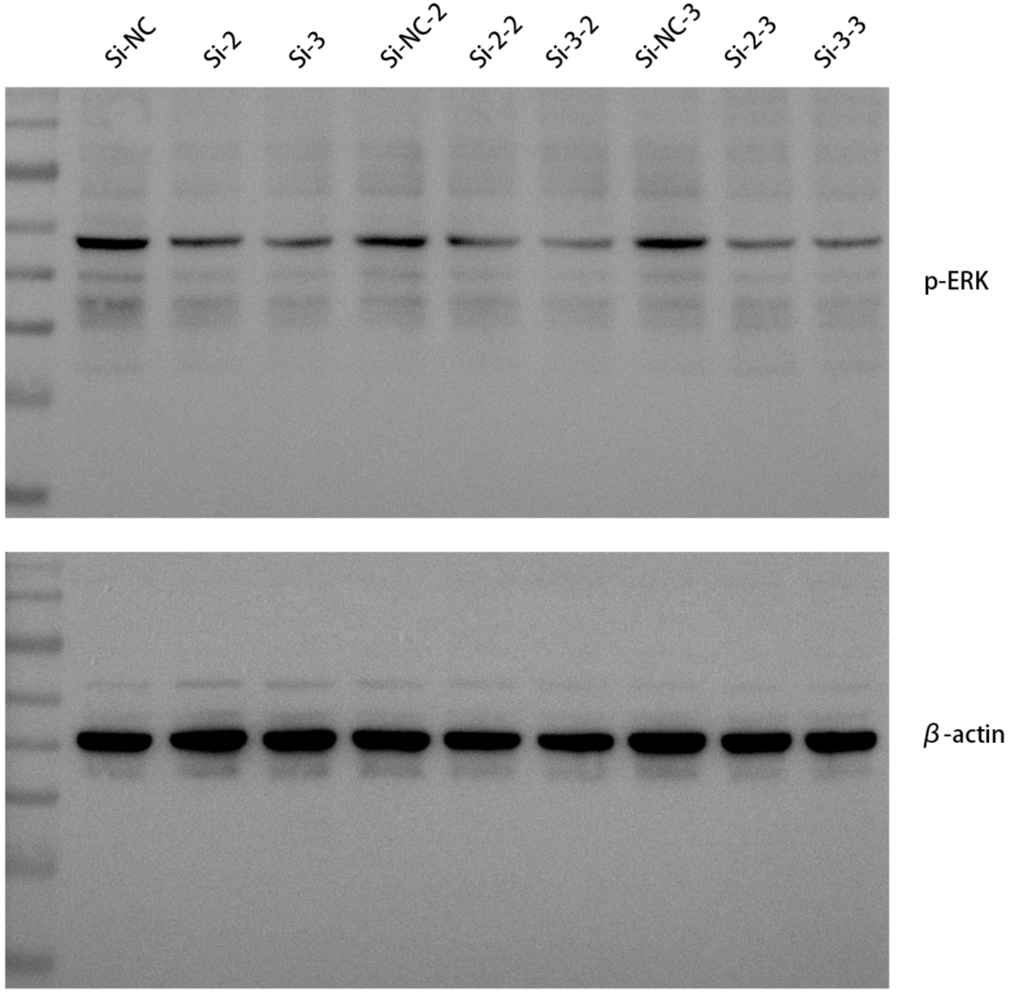

FIG7_A_tumor

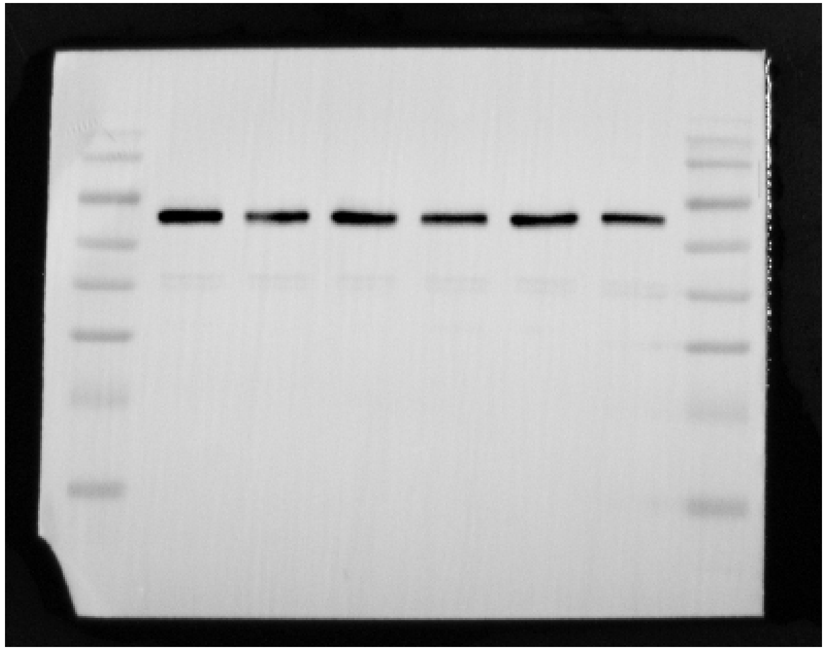

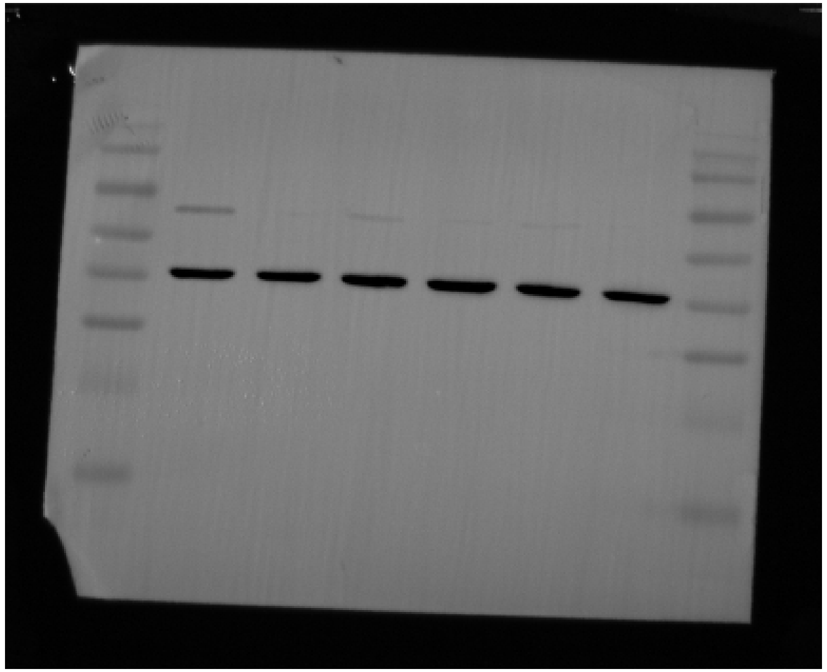

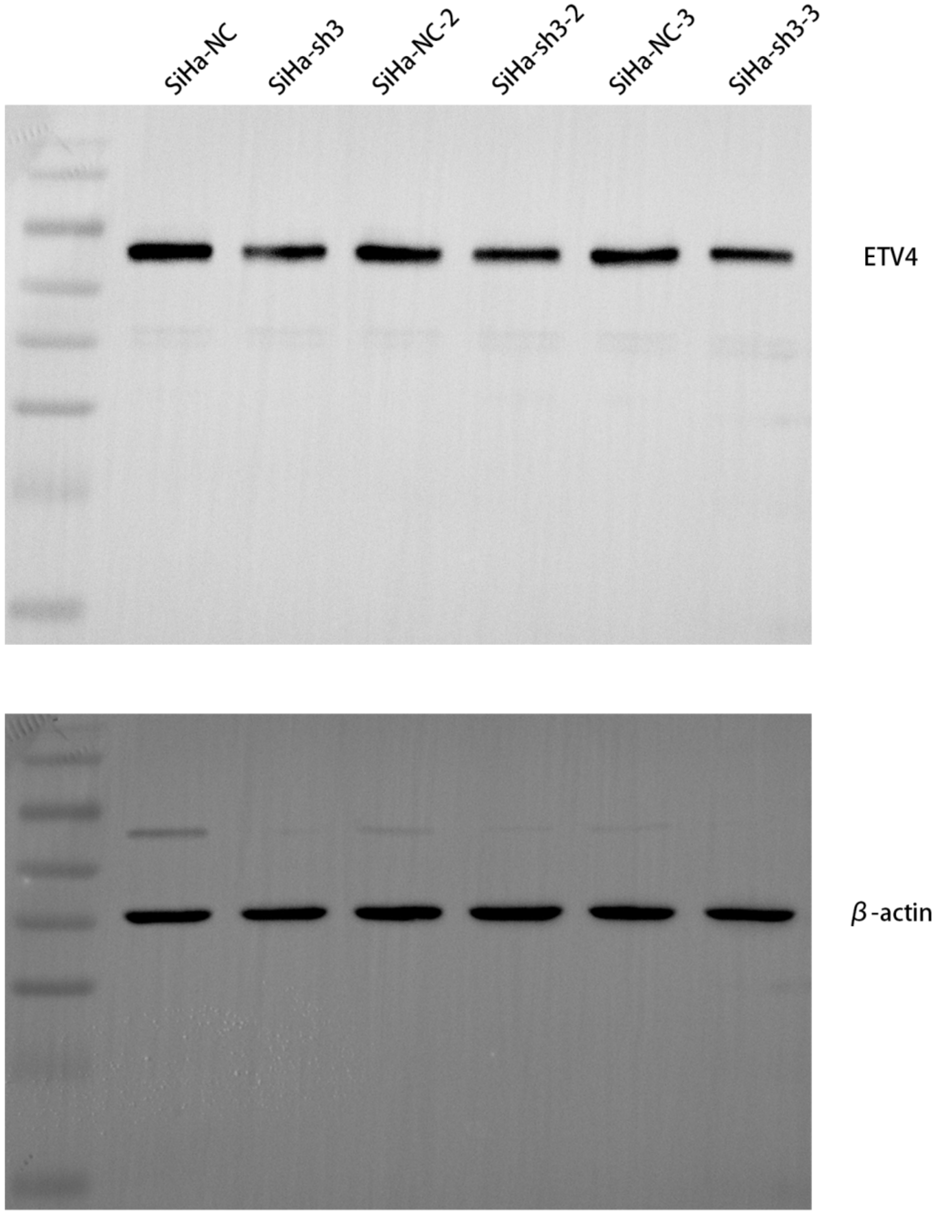
SiHa-NC
SiHa-sh3
SiHa-NC-2
SiHa-sh3-2
SiHa-NC-3
SiHa-sh3-3
ETV4
β-actin

FIG7_D_Knockdown of ETV4_HeLa

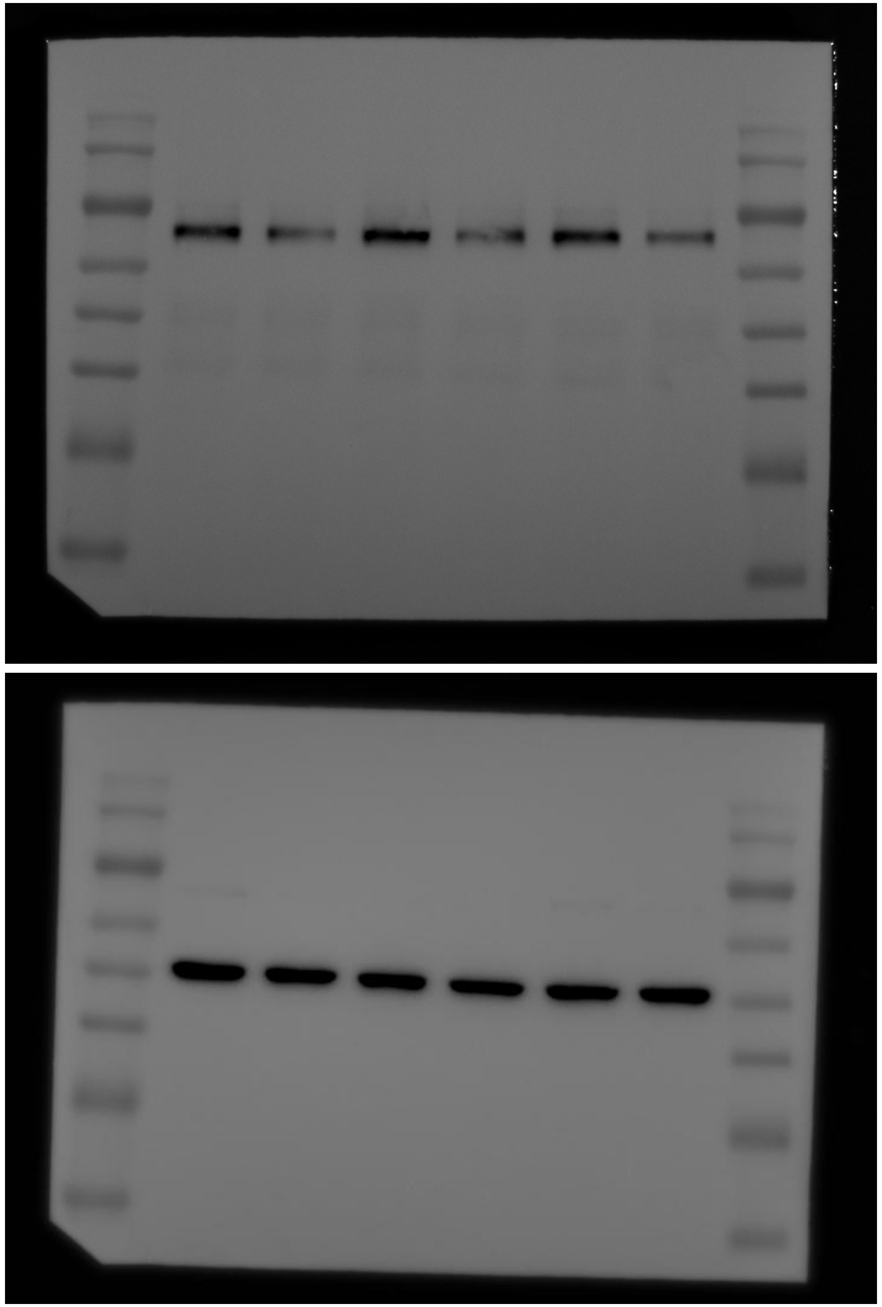

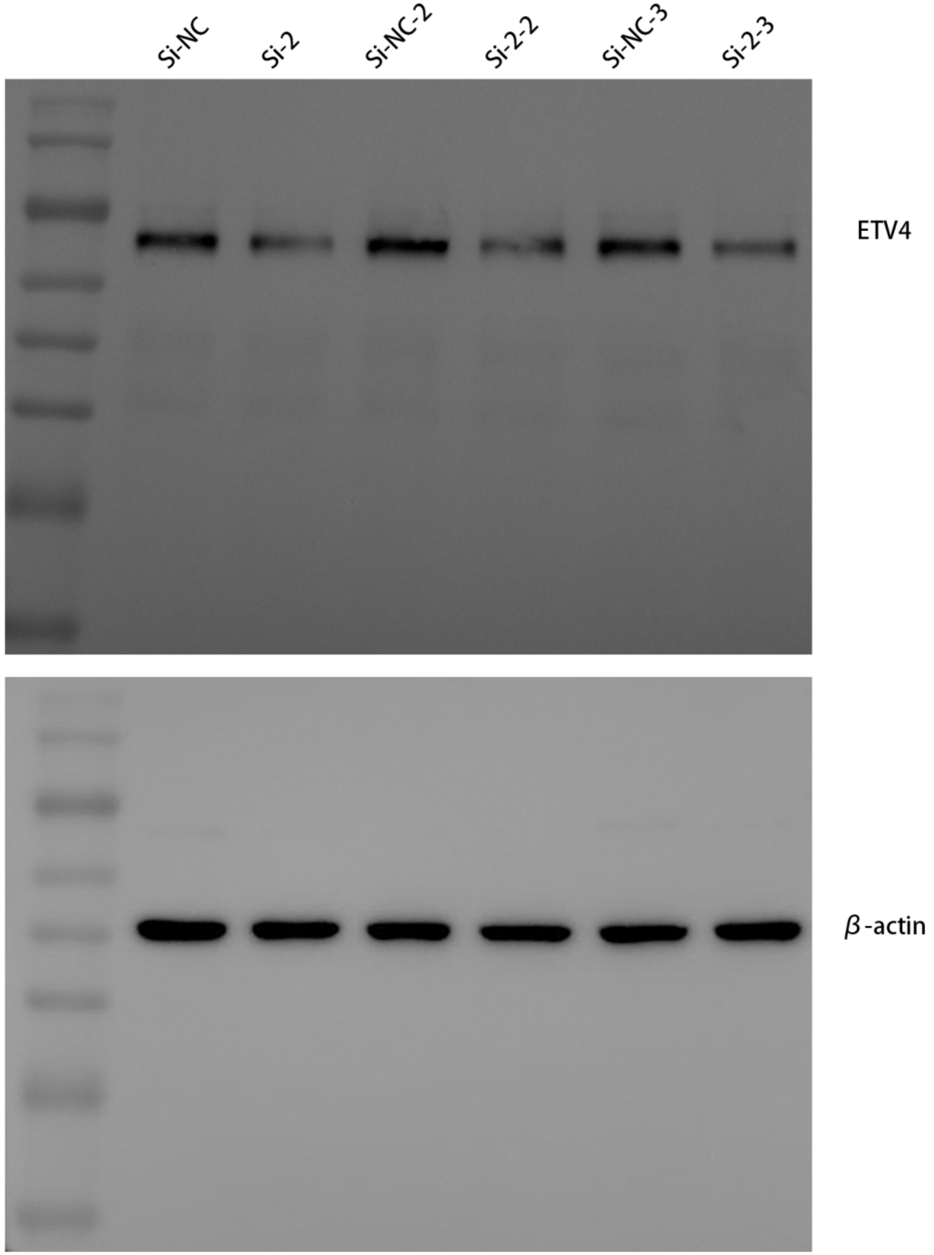

FIG7_D_Knockdown of ETV4_SiHa

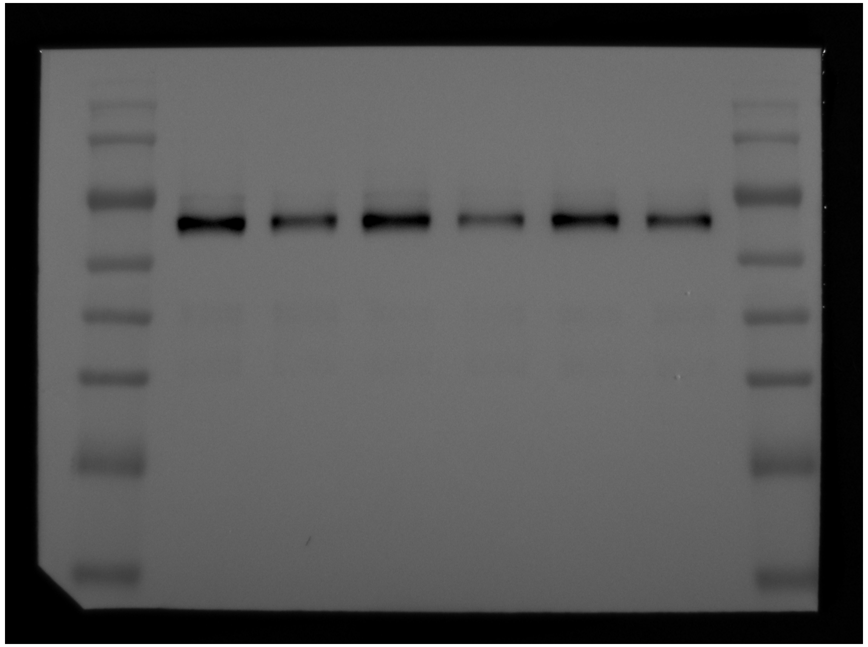

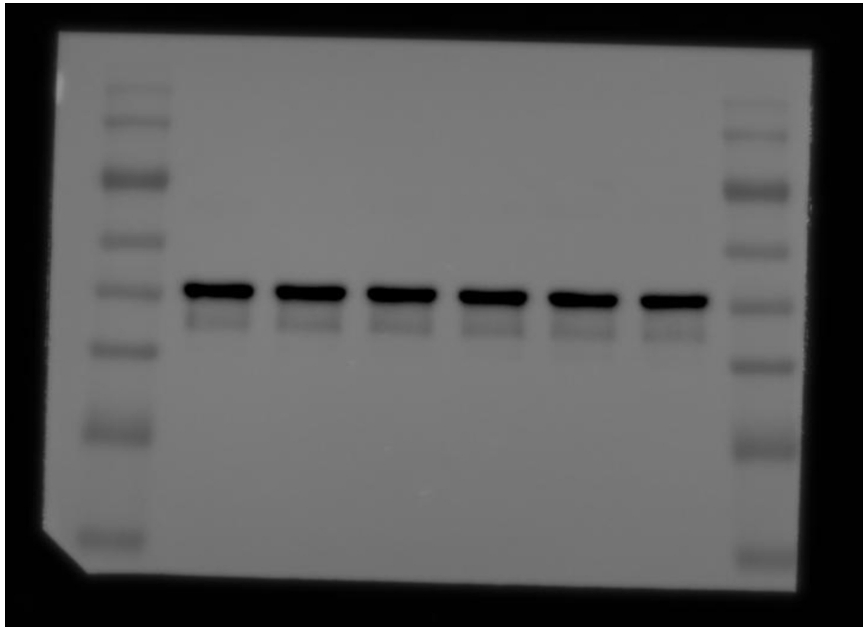

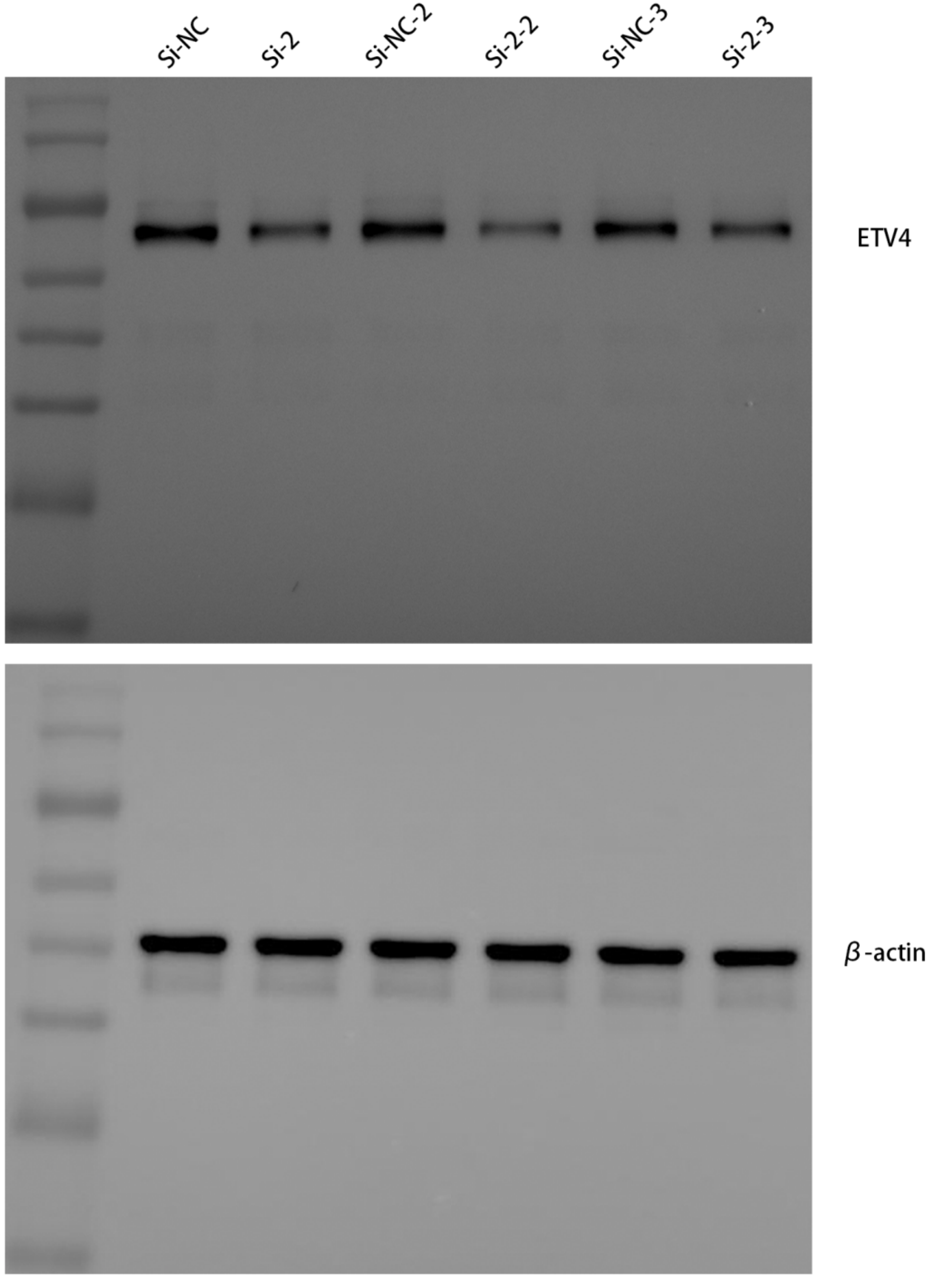

FIG7_G_ETV4

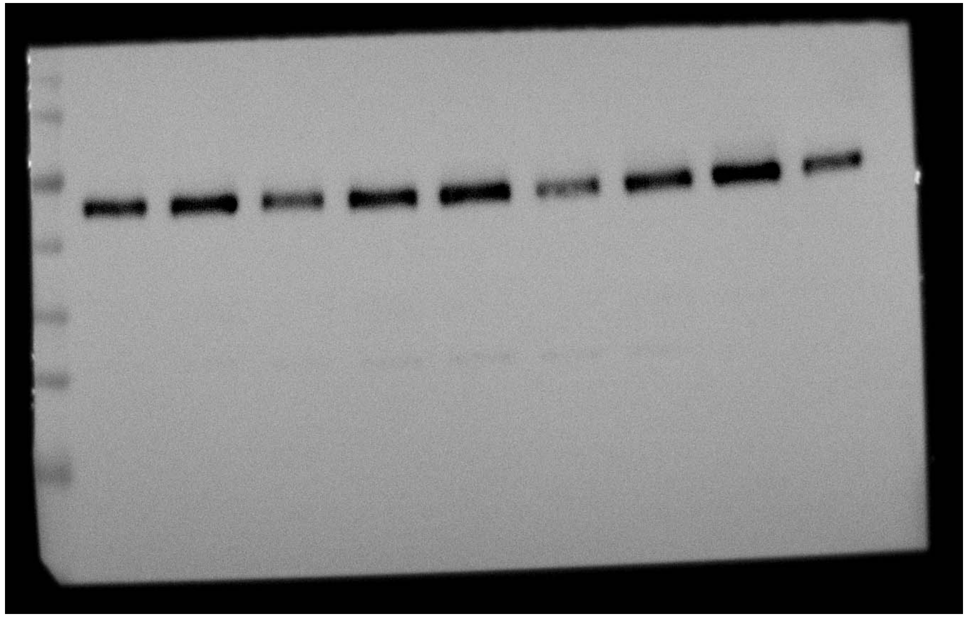

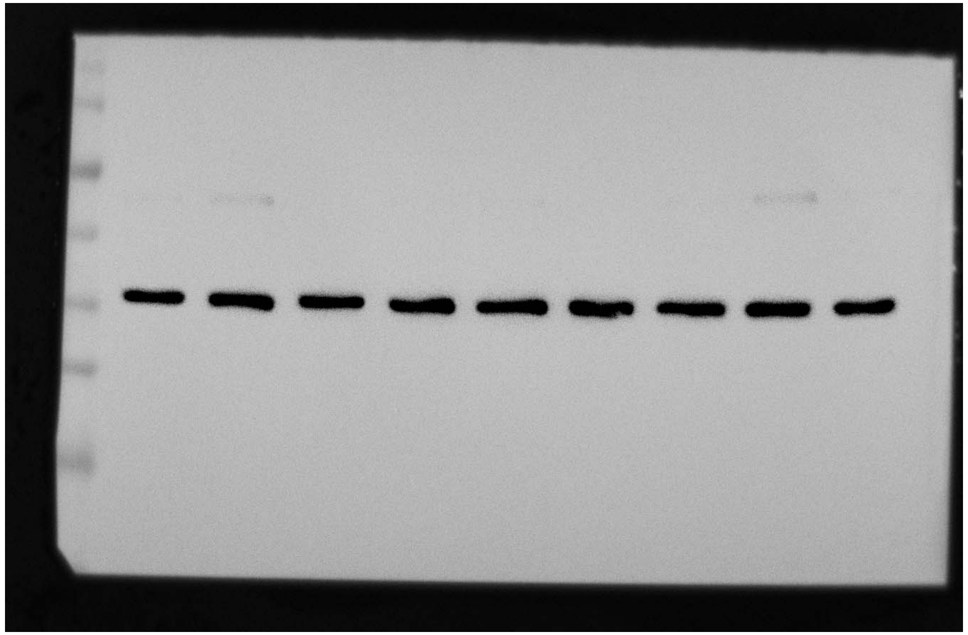

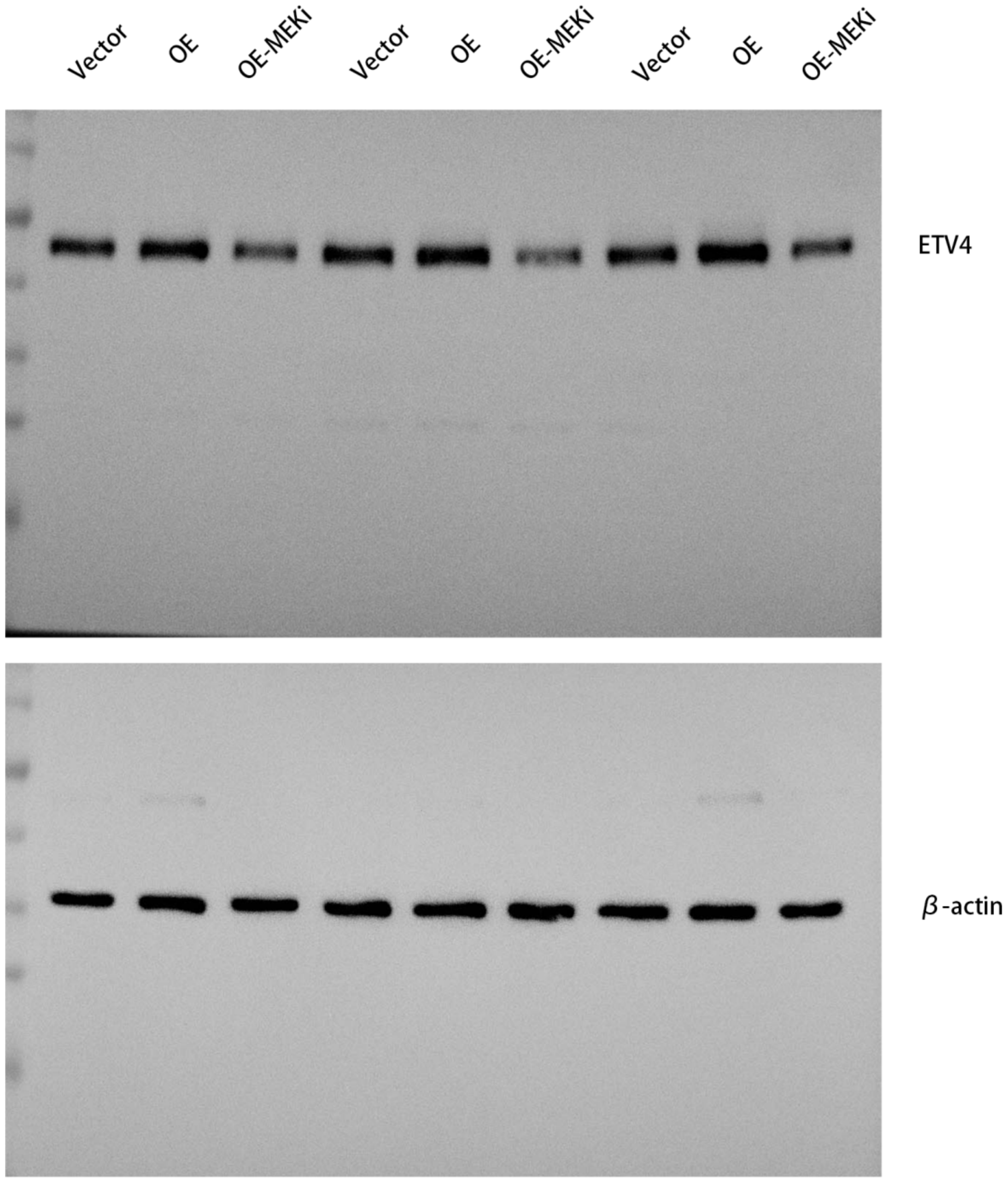
Vector
OE
OE-MEKi
Vector
OE
OE-MEKi
Vector
OE
OE-MEKi
ETV4
β-actin

FIG7_G_ERK

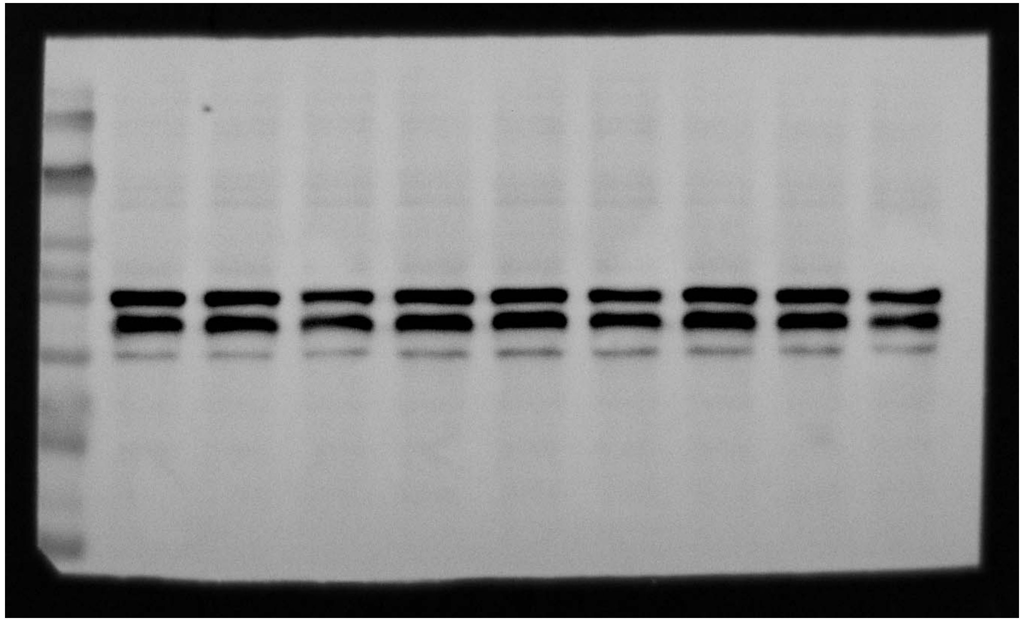

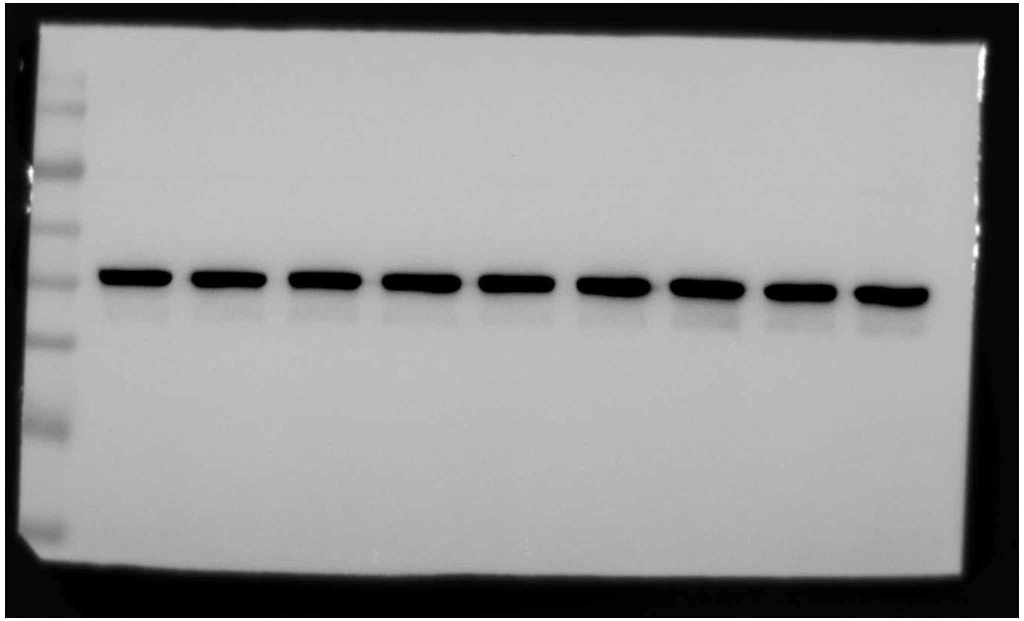

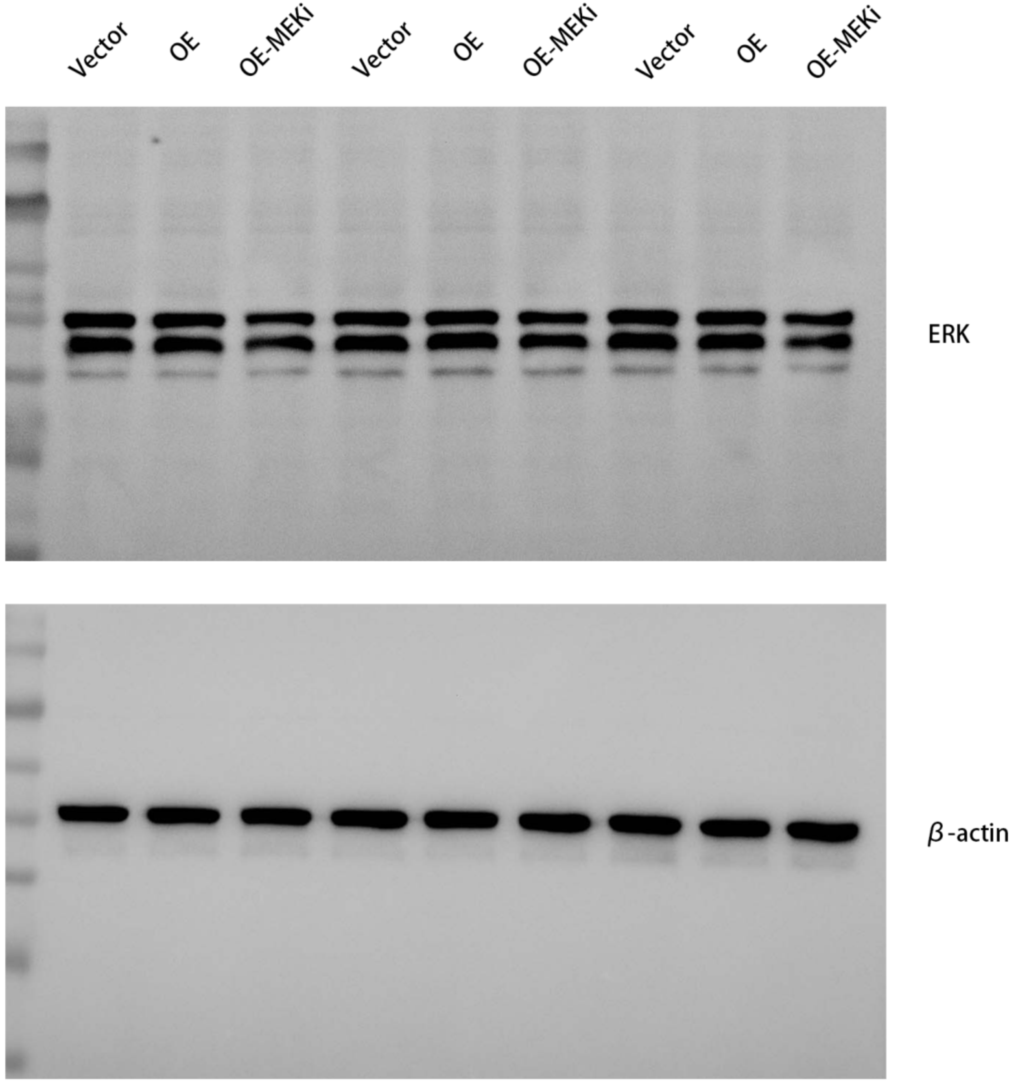
Vector
OE
OE-MEKi
Vector
OE
OE-MEKi
Vector
OE
OE-MEKi
ERK
β-actin

FIG7_G_p-ERK

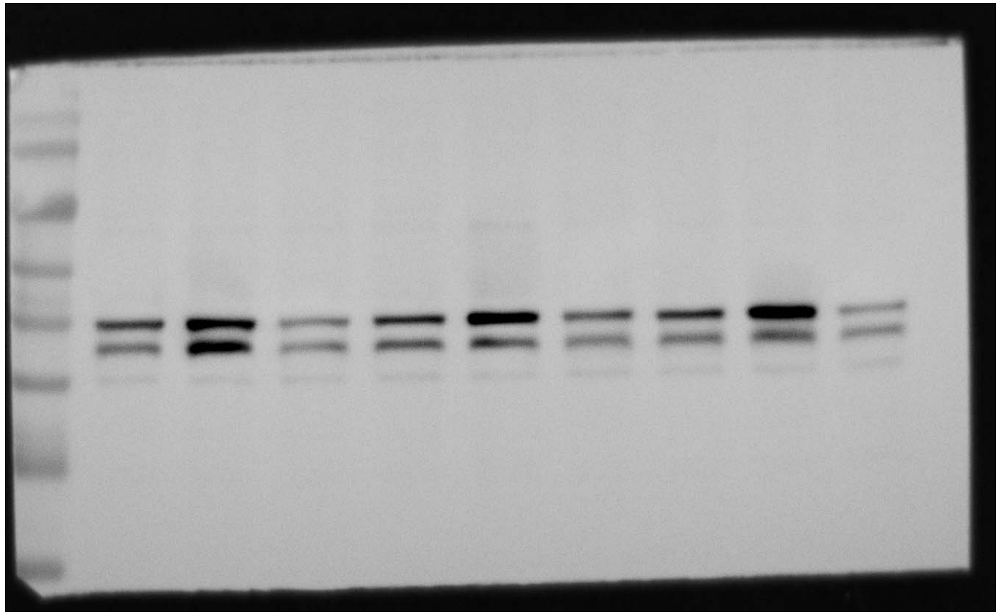

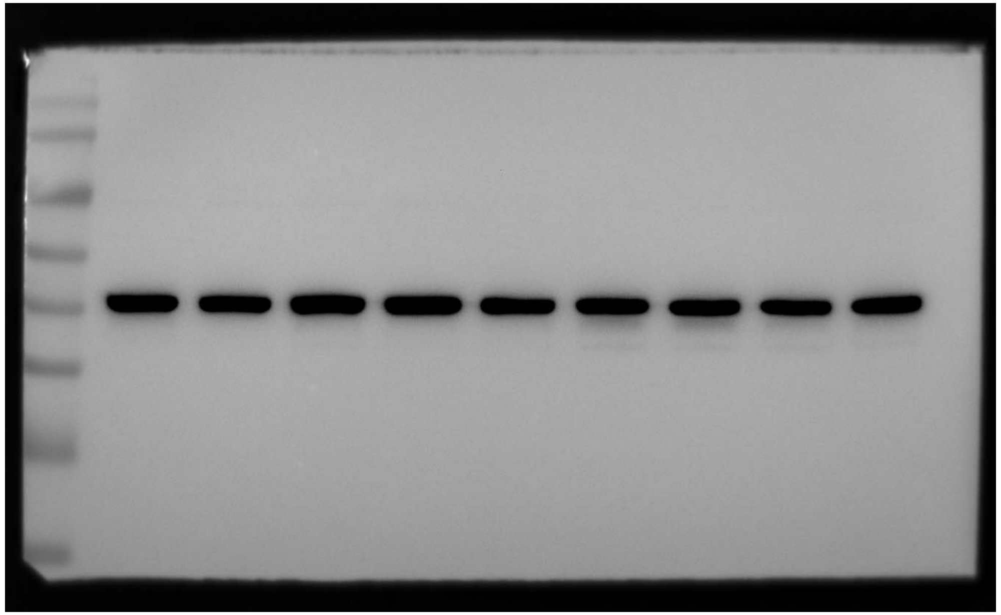

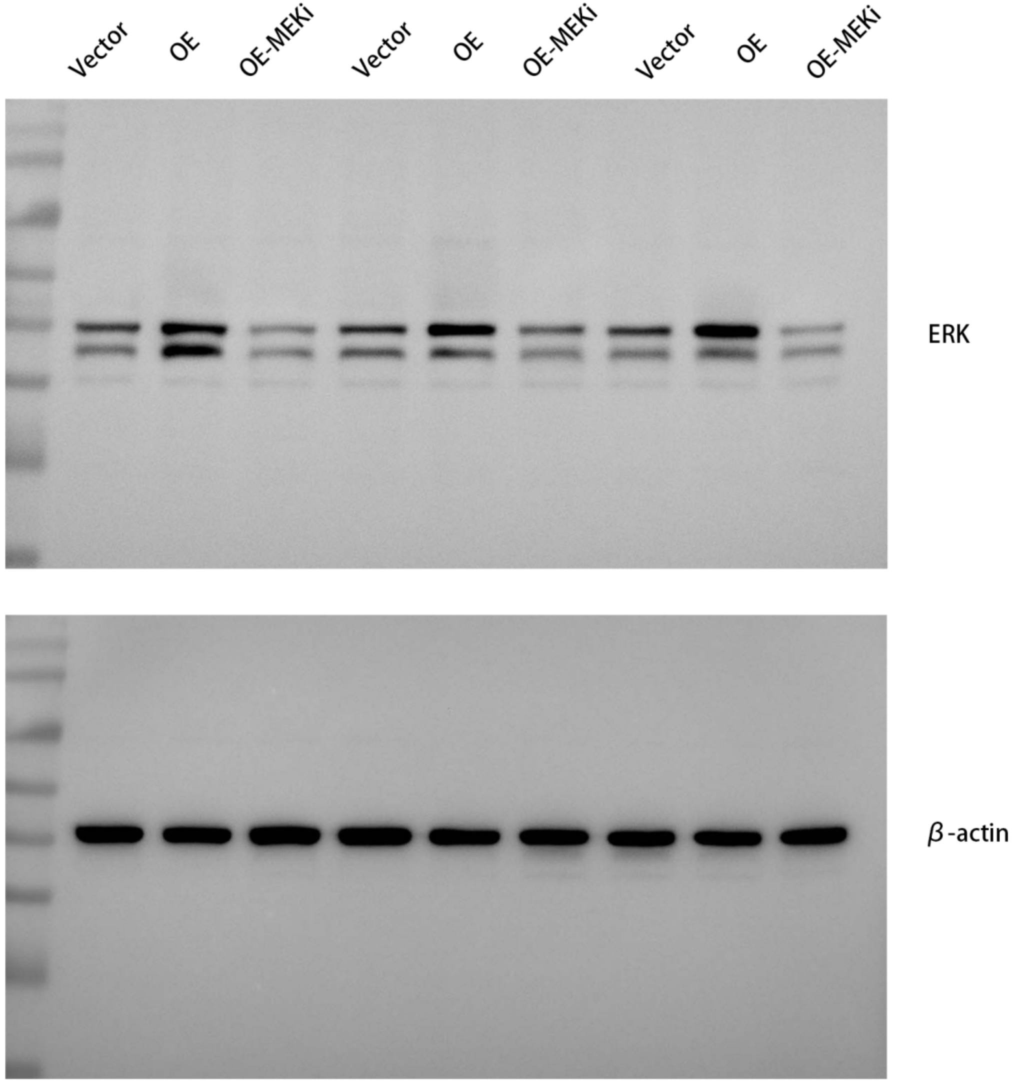
Vector
OE
OE-MEKi
Vector
OE
OE-MEKi
Vector
OE
OE-MEKi
ERK
β-actin
